# Improvement of Analysis and Transferability in Peptide Purification: From HPLC to FPLC and Back Again

**DOI:** 10.1002/psc.70090

**Published:** 2026-02-10

**Authors:** Alessandro Streuli, Vanessa Erckes, Brunello Nardone, Vincent Bedard, François Beland, Christian Steuer

**Affiliations:** ^1^ Institute of Pharmaceutical Sciences, Laboratory of Pharmaceutical Analytics ETH Zurich Zurich Switzerland; ^2^ Zeochem Silica Materials Quebec Quebec Canada

## Abstract

Optimal purification, high purity and robust purity control for synthetic peptides are critical, as even minor impurities can alter biological activity, distort analytical results and compromise downstream applications. Reliable translation of analytical HPLC results to preparative FPLC conditions remains challenging due to system‐specific differences in column geometry, gradient formation and mobile‐phase chemistry. This study addresses these limitations through a systematic evaluation of chromatographic parameters influencing peptide selectivity, resolution and transferability. Using a defined peptide impurity library, the effects of gradient steepness, flow rate, temperature and mobile‐phase modifier were quantified, revealing that flow‐rate optimization and modifier choice have the greatest impact on separation quality. A correction equation was developed to compensate for system‐dependent deviations, reducing transfer errors in elution percentage from approximately 17% to less than 5%. The optimized workflow enabled initial preparative purifications with purities above 90% and yields exceeding 30%. Additionally, substitution of trifluoroacetic acid with formic acid was explored as a greener modifier, providing selective improvements in separation performance. The approach establishes a practical and sustainable workflow for the transfer of HPLC‐to‐FPLC methods for peptide purification.

## Graphical abstract

A predictive workflow enables accurate transfer from analytical HPLC to preparative FPLC for peptide purification. Systematic evaluation of key chromatographic parameters identified particle size, flow optimization and modifier choice as critical for selectivity and resolution. A correction model minimized elution transfer errors from −17% to less than −5%, achieving first‐pass purifications with purities higher than > 90%.

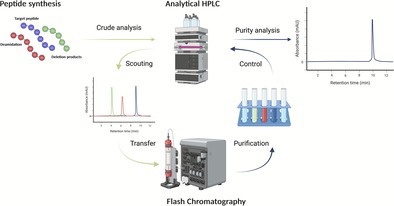

## 1 Introduction

Synthetic peptides have emerged as an important class of therapeutic agents owing to their ability to modulate biological processes with high potency and specificity, particularly by targeting protein–protein interactions that are often inaccessible to conventional small molecules [1, 2]. Over the past decade, several peptide‐based drugs such as the GLP‐1 analogues Semaglutide (Ozempic, Wegovy) and Tirzepatide (Mounjaro) [3, 4], Bremelanotide (Vyleesi) for hypoactive sexual desire disorder [5] and Setmelanotide (Imcivree) for obesity‐related genetic disorders [6] have reached clinical approval, whereas many others are in advanced clinical trials [7, 8, 9]. Most of these compounds are at least partially produced via Fmoc solid‐phase peptide synthesis (SPPS), a robust and versatile technique using orthogonal chemistry enabling the rapid assembly of complex peptide sequences with precise control over composition and length [10, 11, 12]. As SPPS relies on repeated cycles of coupling, intermediate Fmoc deprotection and final global deprotection and cleavage, with each step being able to introduce side reactions and by‐products, a variety of closely related impurities may form, including deamidated and isoaspartate‐containing variants, chain truncations or deletions and amino acid misincorporations. As SPPS is an iterative synthesis process, such impurities accumulate along the synthesis [13, 14, 15, 16, 17]. Despite these modifications being mostly minor, they can significantly impact peptide function and characteristics and, if not adequately controlled, may adversely affect the quality and safety of the peptide [18, 19, 20, 21]. Due to their subtle mass and physicochemical differences, they can exhibit chromatographic behaviour similar to the parent compound and are therefore notoriously difficult to separate and detect, posing significant analytical and preparative challenges [15, 22]. This challenge is further amplified by the fact that various impurities, such as isoaspartate variants or deamidated species, share identical or near‐identical masses with the target peptide, rendering mass spectrometry insufficient for purity assessment in the absence of adequate chromatographic resolution. Accurate impurity profiling and reliable purification strategies are therefore critical to ensure both product quality and process efficiency [23]. High‐performance liquid chromatography (HPLC) is the method of choice, and the most frequently described analytical technique, for the separation and purity determination of synthetic peptides. Although alternative chromatographic modes such as hydrophilic interaction liquid chromatography (HILIC) [24, 25, 26, 27, 28] and supercritical fluid chromatography (SFC) [29, 30, 31, 32, 33] have been explored, reversed‐phase liquid chromatography (RPLC) using C4, C8 or C18 stationary phases remains the most widely applied technique for peptide analysis due to its excellent resolving power, instrumental simplicity, robustness and versatility [23, 27, 34, 35]. Numerous studies have focused on optimizing analytical peptide separations by investigating the effects of gradient design, stationary‐phase chemistry, mobile‐phase composition and ion‐pairing modifiers such as trifluoroacetic acid (TFA) [23, 34, 35, 36, 37, 38, 39, 40]. However, despite the abundance of published work, methods applied to peptide analysis vary widely in their experimental conditions and parameters, both between laboratories and across studies, even for structurally similar peptides. This variability hampers reliable purity determination, direct comparison and systematic evaluation of reported methods and results [15, 20, 21]. Whereas other fluorinated ion‐pairing reagents such as pentafluoropropionic acid and heptafluorobutyric acid were investigated for their use in peptide analysis and purification [23, 41], TFA is generally regarded as the most effective additive for achieving optimal resolution in RP‐HPLC [23, 42]. However, growing environmental concerns and evidence of unwanted in vitro and in vivo effects have encouraged the use of greener alternatives such as formic acid (FA) or acetic acid (AA) [43, 44, 45, 46, 47, 48, 49]. The impact of such substitutions on chromatographic selectivity, resolution and preparative recovery remains insufficiently explored. In peptide production workflows, preparative purification is frequently carried out using RP‐flash liquid chromatography (FPLC) systems [50, 51, 52]. These platforms are widely employed due to their operational simplicity, scalability and compatibility with aqueous mobile phases, which make them well suited for routine peptide purification and recovery. Whereas preparative HPLC offers superior separation efficiency, FPLC provides advantages in terms of operational simplicity, cost‐effectiveness and high sample‐loading capacity, making it well suited for routine and early‐stage peptide purification. These practical advantages come at the expense of lower intrinsic resolving power, which increases the importance of robust analytical‐to‐preparative method transfer strategies when closely related peptide species must be separated. However, despite conceptual similarities to analytical HPLC, the transfer of chromatographic information between these systems remains challenging. Differences in column geometry, stationary‐phase particle size, system dead volume, gradient delay and overall system dispersion can significantly alter retention behaviour and elution times [53, 54, 55]. As a result, elution conditions predicted from analytical HPLC often fail to reproduce equivalent selectivity or resolution on preparative FPLC systems [56]. These discrepancies typically necessitate additional optimization experiments, increasing solvent consumption, process time and material loss. Therefore, to enable more reliable scale translation, improved understanding of how RP‐HPLC system parameters affect transferability to RP‐FPLC systems is required.

**FIGURE 1:**
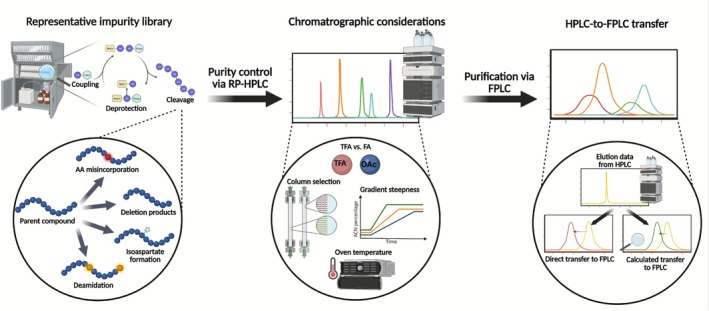
Schematic overview of the study design. Left: generation of a representative peptide impurity library reflecting common SPPS by‐products, including amino acid misincorporation, deletion sequences, deamidation and isoaspartate formation. Middle: chromatographic considerations investigated in this work, including column selection, gradient steepness, flow rate, oven temperature and the choice of ion‐pairing modifier (TFA vs. FA). Right: conceptual workflow for improving HPLC‐to‐FPLC transfer, comparing direct transfer of elution data with the proposed correction model enabling more accurate prediction of preparative elution behaviour. The figure was created in BioRender. Steuer, C. (2026) https://BioRender.com/atpe52s.

The present study addresses analytical challenges in peptide‐driven drug discovery projects by systematically evaluating chromatographic parameters that influence peptide separation and purification using a comprehensive impurity library. As illustrated in Figure 1, we investigated the effects of method shortening, column geometry, gradient steepness, flow rate, temperature and mobile‐phase modifier on the separation efficiency and selectivity using a peptide library comprising more than 20 synthetic species. The library includes distinct groups of structurally related peptides specifically designed to represent common impurity types encountered in solid‐phase peptide synthesis, such as deamidation, isoaspartate formation, leucine/isoleucine isomers and deletion products. A central focus of this work is the comparison of columns with a 10‐μm particle size, motivated by our hypothesis that larger particle sizes might more closely resemble FPLC columns and therefore may facilitate analytical‐to‐FPLC method transfer while still providing sufficient resolution for reliable purity determination. Furthermore, we propose and evaluate a correction equation aimed at improving the accuracy of analytical‐to‐preparative method transfer. Our correction equation enables direct translation of analytical chromatographic data into FPLC conditions, facilitating initial preparative purification (i.e., the first purification step performed without iterative reoptimization) and contributing to improved efficiency, reproducibility and sustainability of peptide purification.

## 2 Materials and Methods

### 2.1 Library Generation and Impurity Class Definition

**FIGURE 2:**
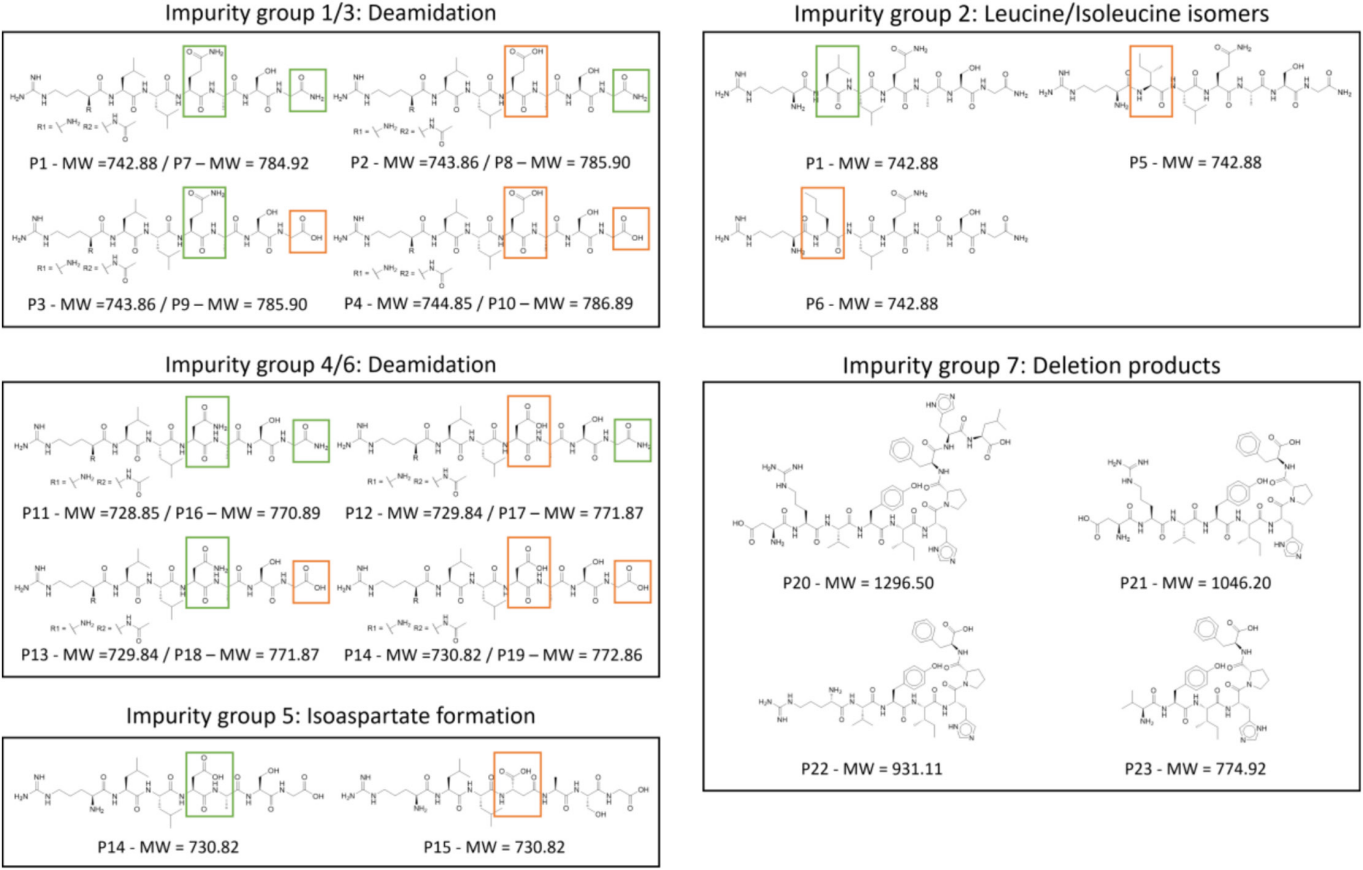
Graphical overview of Impurity Groups 1–7 included in the peptide impurity library. For each peptide, the original structural element is highlighted in green, whereas deviations responsible for the impurity are marked in orange. Impurity Groups 1/3 and 4/6 correspond to deamidation variants, Group 2 represents leucine/isoleucine isomers, Group 5 illustrates isoaspartate formation and Group 7 contains deletion products. Groups 1 and 3 as well as 4 and 6 were combined, as they differ only by N‐terminal acetylation. The nonacetylated (Groups 1 and 4) and acetylated (Groups 3 and 6) forms are denoted as *R*_1_ and *R*_2_, respectively. Molecular weights (MW) of each species are indicated below the structures.

All peptides listed in Table 1 were individually synthesized via standard Fmoc solid‐phase peptide synthesis (SPPS) using a PurePep Chorus peptide synthesizer (Gyros Protein Technologies, Warren, NJ, United States). The resulting library comprised 23 peptides, including five target peptides (P1, P7, P11, P16 and P20) and a set of deliberately synthesized, structurally related peptides designed to represent plausible synthesis‐related impurities, which were classified into seven impurity groups reflecting common impurity patterns (Table 1). All peptides served as nonbioactive model scaffolds selected solely for their suitability to represent typical synthesis‐related impurity profiles. For each peptide, the calculated LogP and topological polar surface area (TPSA) are also reported to illustrate the high physicochemical similarity within and across impurity groups. A schematic overview of all defined impurity groups and their corresponding structural modifications is shown in Figure 2.

**TABLE 1:** Overview of the synthesized peptide library consisting of 23 peptides. Modifications on the N‐ and C‐termini, molecular weight (MW), LogP, TPSA and the corresponding impurity groups are indicated. Deamidation products bearing acidic modifications are highlighted in bold, whereas impurities arising from isomeric variants are shown in italic. MWs, LogPs and TPSAs were calculated using PICKAPEP [57].

Label	Impurity groups	Sequence	N term	C term	MW (Da)	LogP	TPSA
P1	1,2	RLLQASG	‐NH_2_	‐CONH_2_	742.88	−5.03	368.93
P2	1	RLL**E**ASG	‐NH_2_	‐CONH_2_	743.86	−4.43	363.14
P3		RLLQASG	‐NH_2_	**COOH**	743.86	−4.43	363.14
P4		RLL**E**ASG	‐NH_2_	**COOH**	744.85	−3.83	357.35
P5	2	R*I*LQASG	‐NH_2_	‐CONH_2_	742.88	−5.03	368.93
P6		R*Nle*LQASG	‐NH_2_	‐CONH_2_	742.88	−4.88	368.93
P7	3	RLLQASG	Ac‐NH—	‐CONH_2_	784.92	−4.85	372.01
P8		RLL**E**ASG	Ac‐NH—	‐CONH_2_	785.90	−4.25	366.22
P9		RLLQASG	Ac‐NH—	**COOH**	785.90	−4.25	366.22
P10		RLL**E**ASG	Ac‐NH—	**COOH**	786.89	−3.65	360.43
P11	4	RLLNASG	‐NH_2_	‐CONH_2_	728.85	−5.42	368.93
P12		RLL**D**ASG	‐NH_2_	‐CONH_2_	729.84	−4.82	363.14
P13		RLLNASG	‐NH_2_	**COOH**	729.84	−4.82	363.14
P14	4,5	RLL**D**ASG	‐NH_2_	**COOH**	730.82	−4.22	357.35
P15	5	RLL*Diso*ASG	‐NH_2_	**COOH**	730.82	−4.22	357.35
P16	6	RLLNASG	Ac‐NH—	‐CONH_2_	770.89	−5.24	372.01
P17		RLL**D**ASG	Ac‐NH—	‐CONH_2_	771.87	−4.64	366.22
P18		RLLNASG	Ac‐NH—	**COOH**	771.87	−4.64	366.22
P19		RLL**D**ASG	Ac‐NH—	**COOH**	772.86	−4.04	360.43
P20	7	DRVYIHPFHL	‐NH_2_	**COOH**	1296.5	−1.12	493.22
P21		DRVYIHPF	‐NH_2_	**COOH**	1046.2	−1.11	406.34
P22		RVYIHPF	‐NH_2_	**COOH**	931.11	−0.06	339.94
P23		VYIHPF	‐NH_2_	**COOH**	774.92	1.19	248.94

Unless otherwise stated, peptides were purified using a puriFlash XS520Plus flash chromatography system (FPLC) in combination with the SiliaSep PREMIUM flash cartridges (C18, 12 g, 25 μm, 90 Å, Zeochem Silica Materials, Quebec, Canada) to a purity of > 80%. Detailed procedures are provided in the Supporting Information. Peptide identities were confirmed by LC‐MS analysis using a dedicated LC‐MS method described in the Supporting Information. The LC‐MS method was employed exclusively for identity confirmation. Initial peptide purities were determined by LC‐UV using the following reference method, which served as the basis for subsequent method optimization. Purity analysis was performed using a VWR ELITE Lachrom Series LC system equipped with a UV detector (VWR International, Dietikon, Switzerland) using individual peptide solutions at a concentration of 0.1 mg/mL. Chromatographic separations were performed on a Sunfire C18 column (3.5 μm, 3.0 × 150 mm; Waters Corporation, Milford, MA, United States). The mobile phases consisted of 0.1% TFA in acetonitrile (ACN, HPLC gradient grade, ≥ 99.9%, Sigma Aldrich; Eluent A) and 0.1% trifluoroacetic acid (TFA) in water (Eluent B). The gradient used, with a flow rate of 1.0 mL min^−1^, was as follows: 0.0–5.0 min at 5% A, 5.0–50.0 min to 95% A, 50.0–55.0 min at 95% A, 55.0–56.0 min to 5% A, and 56.0–65.0 min re‐equilibration with 5.0% A. The column temperature was maintained at 30°C, and the injection volume was 10 μL. UV detection was carried out at 214 nm, with a sampling frequency of 200 ms and a response time of 1 s. Data acquisition and processing were performed using OpenLab software (Version A.04.08; Agilent Technologies, Santa Clara, CA, United States).

### 2.2 Identifying Critical Parameters via HPLC‐UV

To identify the chromatographic parameters most critical for reliable purity determination, a series of column and method scouting experiments was conducted. Three scouting columns, the SiliCycle ResiPure Advanced C18 (10 μm, 4.6 × 250 mm, 150 Å; Zeochem Silica Materials, Quebec, Canada), SiliaChrom Plus HPLC C18 (10 μm, 4.6 × 250 mm, 100 Å; Zeochem Silica Materials, Quebec, Canada) and InnoPeptide C18 (10 μm, 4.6 × 250 mm, 100 Å; Zeochem Silica Materials, Quebec, Canada) were evaluated against two analytical reference columns, a Sunfire C18 (3.5 μm, 3.0 × 150 mm; Waters Corporation, Milford, MA, United States) and a Discovery C18 (5 μm, 4.6 × 250 mm; Supelco Analytical, Bellefonte, PA, United States). The Sunfire C18 represents the smallest particle analytical column compatible with the pressure limits of the HPLC system used (400 bar) and was therefore selected as an upper‐bound reference for analytical separation efficiency, rather than for strict geometrical equivalence. The chromatographic method described in Section 2.1 was applied with a cropped gradient (0.0–5.0 min at 5% A, 5.0–25.0 min to 45% A, 25.0–26.0 min to 95% A, 26.0–30.0 min at 95% A, 30.0–31.0 min to 5% A and 31.0–36.0 min re‐equilibration with 5% A) for column comparison. To evaluate potential retention shifts caused by the cropped gradient, all 23 peptides were first analysed individually using the original gradient and compared to their retention times obtained under the cropped gradient on the Sunfire C18. For impurity‐group experiments, peptides were additionally analysed as defined mixtures according to Table 1, and resolution was evaluated within each impurity group. All other chromatographic conditions were identical to those described in Section 2.1. Gradient steepness, flow rate, column temperature and mobile‐phase modifier were subsequently investigated as critical variables. The gradient steepness was increased relative to the original 2% A/min method to 5% (0.0–5.0 min at 5% A, 5.0–23.0 min to 95% A, 23.0–28.0 min at 95% A, 28.0–29.0 min to 5% A and 29.0–38.0 min re‐equilibration with 5% A) and 10% (0.0–5.0 min at 5% A, 5.0–14.0 min to 95% A, 14.0–19.0 min at 95% A, 19.0–20.0 min to 5% A and 20.0–29.0 min re‐equilibration with 5% A). All other additional experiments were performed using the cropped gradient while increasing the oven temperature to 40°C and 50°C and raising the flow rate to 1.5 and 2.0 mL/min, provided that the backpressure remained within column specifications. The influence of the mobile‐phase modifier was examined by replacing 0.1% TFA with 0.1% formic acid (FA) in both eluents. Chromatographic performance with respect to separation efficiency and selectivity was evaluated based on (i) complete detectability of all peptide species within a given impurity group as individual chromatographic peaks, (ii) the number of theoretical plates (*N*) and (iii) chromatographic resolution (*R*_s_).

### 2.3 Transferring HPLC Results to FPLC

To correct for system‐specific differences during HPLC‐to‐FPLC method transfer, the effective ACN percentage for the FPLC elution point was recalculated by correcting the observed retention time for the combined system delay time (column and dwell volume) using the following correction equation:

(1) $$\mathrm{ACN}\;\left(\%\right)=\left(R_{t}-C_{t}-D_{t}\right)\times \text{Gradient}-A{\mathrm{CN}}_{\text{Start}}\left(\%\right)$$

where *Rₜ* is the retention time (min), *Gradient* is the gradient slope (% ACN per min), *ACN*_*Start*_ is the ACN concentration in the isocratic holding time, *Cₜ* is the column volume divided by the flow rate (min) and *Dₜ* is the dwell volume of the HPLC system divided by the flow rate (min). For the reported HPLC system, the dwell volume was determined as 1.77 mL, the column volume for the columns with the dimensions of 4.6 × 250 mm as 5.92 mL and for the column with the dimension of 3.0 × 150 mm as 2.83 mL. To validate the described correction equation, six peptides (P8–P10 and P17–P19) were repurified on the above described FPLC system. For this, approximately 50 mg of crude peptide was dissolved in a minimal volume of DMSO (100–200 μL), and the entire solution was loaded onto the FPLC column. Gradients were started and ended 10% below and above the calculated ACN percentage. Fractions were collected in a targeted manner based on the UV signal at 214 nm, using a fraction volume of 15 mL. Water and acetonitrile containing 0.2% TFA were used as solvents [51]. The gradient slope was set to a change of 0.67% ACN/column volume (CV). The resulting solvent composition for the elution on the FPLC was compared with the HPLC elution composition and with the corrected values. Fractions were checked for their purity using the SiliCycle ResiPure Advanced C18 as described above. To determine the accuracy of the described correction equation, the deviation between the measured and predicted or calculated values was determined at each flow rate for each column.

### 2.4 Spiked Impurity Groups and Evaluation of Purification Performance

In the final part of the study, selected impurity groups were recreated by spiking defined peptide mixtures representing the impurity groups to evaluate the applicability of the optimized separation parameters and the transferability to FPLC. Impurity Groups 1–4 and 6 were prepared according to the compositions listed in Table 1. Slight variations were introduced for Groups 1, 3, 4 and 6 by removing one deamidation species (P2 for Group 1, P9 for Group 3, P13 for Group 4 and P18 for Group 6) in all groups. Spiked groups were subjected to purification using the FPLC conditions described in Section 2.3. To increase recovery, the gradient was changed to 0.44% ACN/CV, and the fraction volume was set to 5 mL. Recovery was determined from the collected fraction weight, and the purity of both the fractions and the lyophilized peptides was analysed as described in Section 2.3. Recovery was calculated gravimetrically after lyophilization as the ratio of the mass of the collected fraction to the initial mass of peptide material loaded onto the cartridge. Purity was determined by LC‐UV area normalization.

## 3 Results and Discussion

### 3.1 Effect of Column Type, Gradient Steepness and Method Shortening on Peptide Separation

**FIGURE 3:**
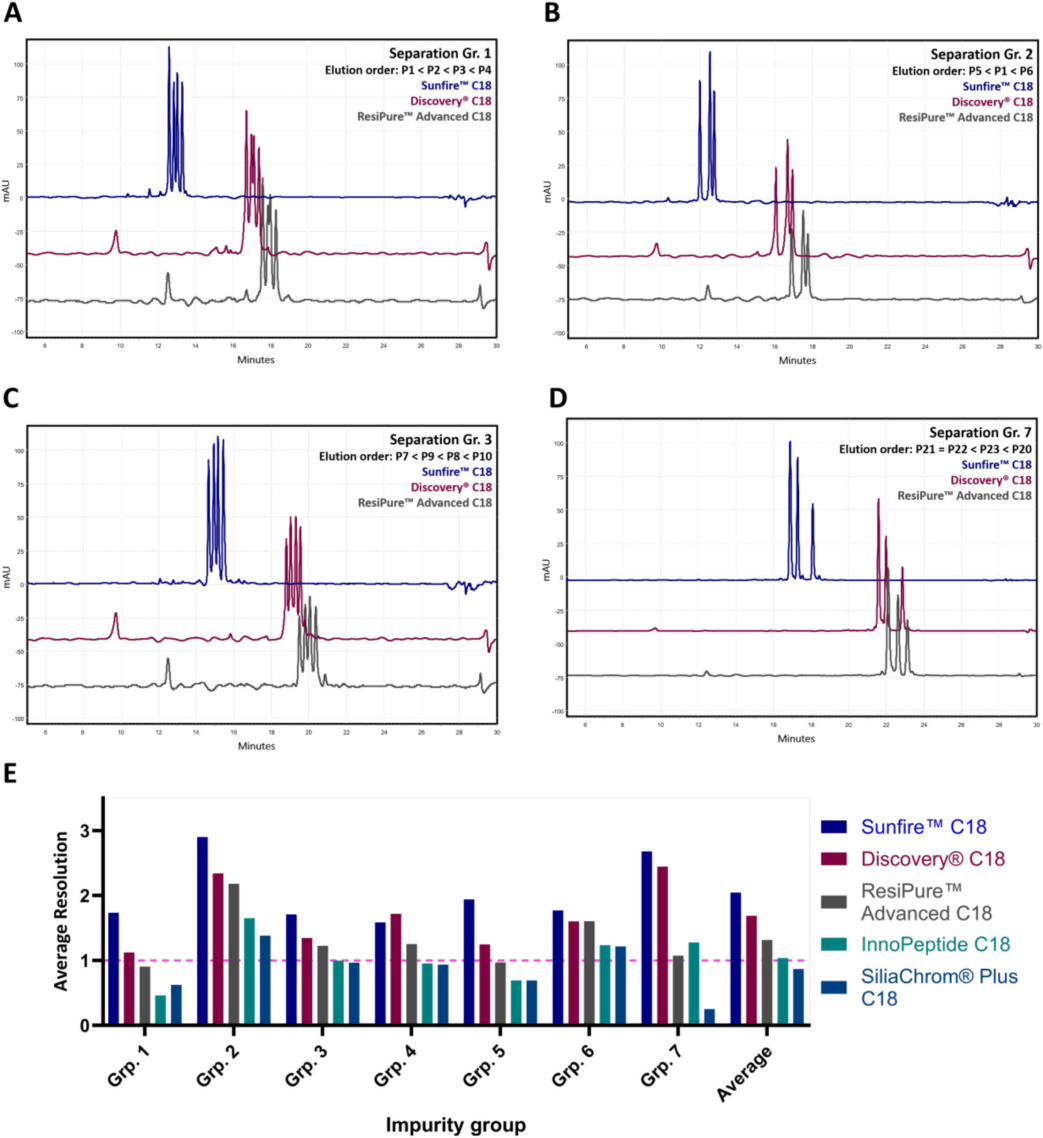
Representation of the impact of column selection on separation efficacy of synthetic peptides. (A–D) Overlaid chromatograms of Impurity Groups 1, 2, 3 and 7, measured on the Sunfire C18 (blue), Discovery C18 (red) and ResiPure Advanced C18 (grey) columns. (E) Average resolution values for the separations obtained with each column for all impurity groups as well as the overall mean resolution. The dashed pink line marks *R_s_* = 1.0, a commonly accepted threshold at which two analytes show a peak overlap of 2.3% and are therefore reliably distinguishable and can be quantified without mutual interference, even if full baseline separation is not achieved.

Shortening run times while maintaining resolution and separation efficacy is essential to meet the growing demand for cost‐effective but also greener analytical methods for high‐throughput laboratories. To compensate for the shortened methods, a clear trend towards a reduction of particle size and increasing pore size to enhance chromatographic separation efficiency can be observed. In our current study on peptide separation and their purity determination, we systematically compared the performance of 10‐μm columns with that of columns containing smaller particles (3.5 and 5 μm). As expected, the smaller particle analytical columns generally provided higher resolving power; however, the 10‐μm ResiPure Advanced C18 delivered surprisingly comparable separation across Impurity Groups 1–7 and afforded sufficient resolution for reliable quality and purity assessment of synthetic peptides. Applying the cropped method, we found that as expected, among all columns, the Sunfire C18 (3.5 μm) showed the highest overall performance with an average *R*_*s*_ = 2.05 and one impurity group, Group 7, in which one pair was not resolved. The Discovery C18 (5 μm) followed with a mean *R*_*s*_ of 1.69 as well as Impurity Group 7 in which two species were not completely resolved. The ResiPure Advanced C18, despite its 10‐μm particle size, showed comparable selectivity (average *R*_*s*_ = 1.32), with Groups 4 and 7 exhibiting two unresolved pairs each. Representative chromatograms for the three columns of Groups 1–3 and 7 are shown in Figure 3A–D, with summarized resolution data for all columns in Figure 3E (chromatograms can be found in Supporting Information S2.1–S2.5). Both previous generation scouting columns, the InnoPeptide C18 and SiliaChrom Plus, showed less separation power with mean *R*_*s*_ values of 1.04 and 0.87 respectively. These columns were therefore excluded from further experiments.

**FIGURE 4:**
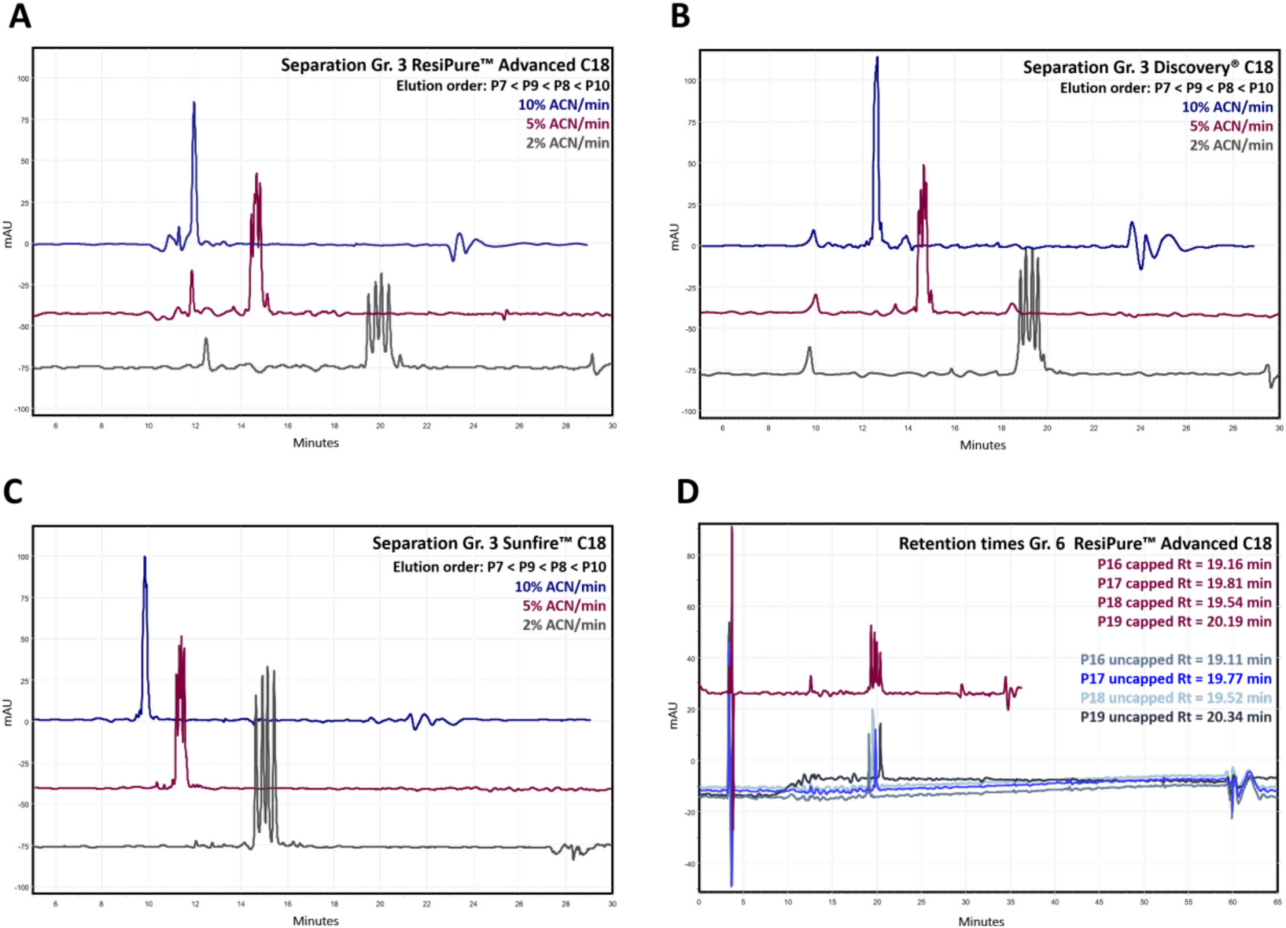
(A–C) Effect of gradient steepness on peptide separation across different C18 columns. Across all columns, increasing gradient steepness reduced resolution and led to coelution of critical peptide pairs, with the strongest effects observed at 10% ACN/min. Overlaid chromatograms of Impurity Group 3 measured on the ResiPure Advanced C18 column using gradient slopes of 2%, 5% and 10% ACN/min. (D) Representation of the impact of method cropping on retention time and separation efficacy of synthetic peptides. Overlaid chromatogram of impurity group 6 (red) alongside single injections of the individual peptides P16–P19 (blue shades) measured on the ResiPure Advanced C18.

Across all columns, increasing gradient steepness to 5% or 10% ACN/min substantially reduced separation quality and efficiency resulting in unsatisfactory purity determination. For the ResiPure Advanced C18, the number of fully resolved groups dropped from 5 to 2 (5% ACN/min) and to none completely resolved groups (10% ACN/min), with mean *R*_*s*_ decreasing from 1.32 to 0.53 and 0.10, respectively. Similar behaviour was observed for the analytical columns: For the Discovery C18, resolved groups decreased from 6 to 3 (5% ACN/min) and to none (10% ACN/min), with mean *R*_*s*_ decreasing from 1.69 to 0.68 and 0.13. For the Sunfire C18, resolved groups decreased from 6 to 4 (5% ACN/min) and none (10% ACN/min), with mean *R*_*s*_ dropping from 2.04 to 0.90 and 0.21. Our findings demonstrate the importance of a sufficiently flat gradient for a reliable purity control of synthetic peptides. Exemplary chromatograms of Impurity Group 3 increasing gradient steepness are shown in Figure 4A–C (chromatograms can be found in Supporting Information S3.1–S3.3). Although gradient steepness is a crucial factor for separating, cropping of the chromatographic method showed negligible influence on retention time for both scouting and analytical columns, provided that re‐equilibration time remained constant (Figure 4D). Method shortening therefore reduced analysis time and solvent use without compromising separation efficiency and represents a more practical method for reducing analysis times than increasing the gradient steepness.

### 3.2 Effect of Flow Rate, Column Temperature and Modifier on Separation Performance

**FIGURE 5:**
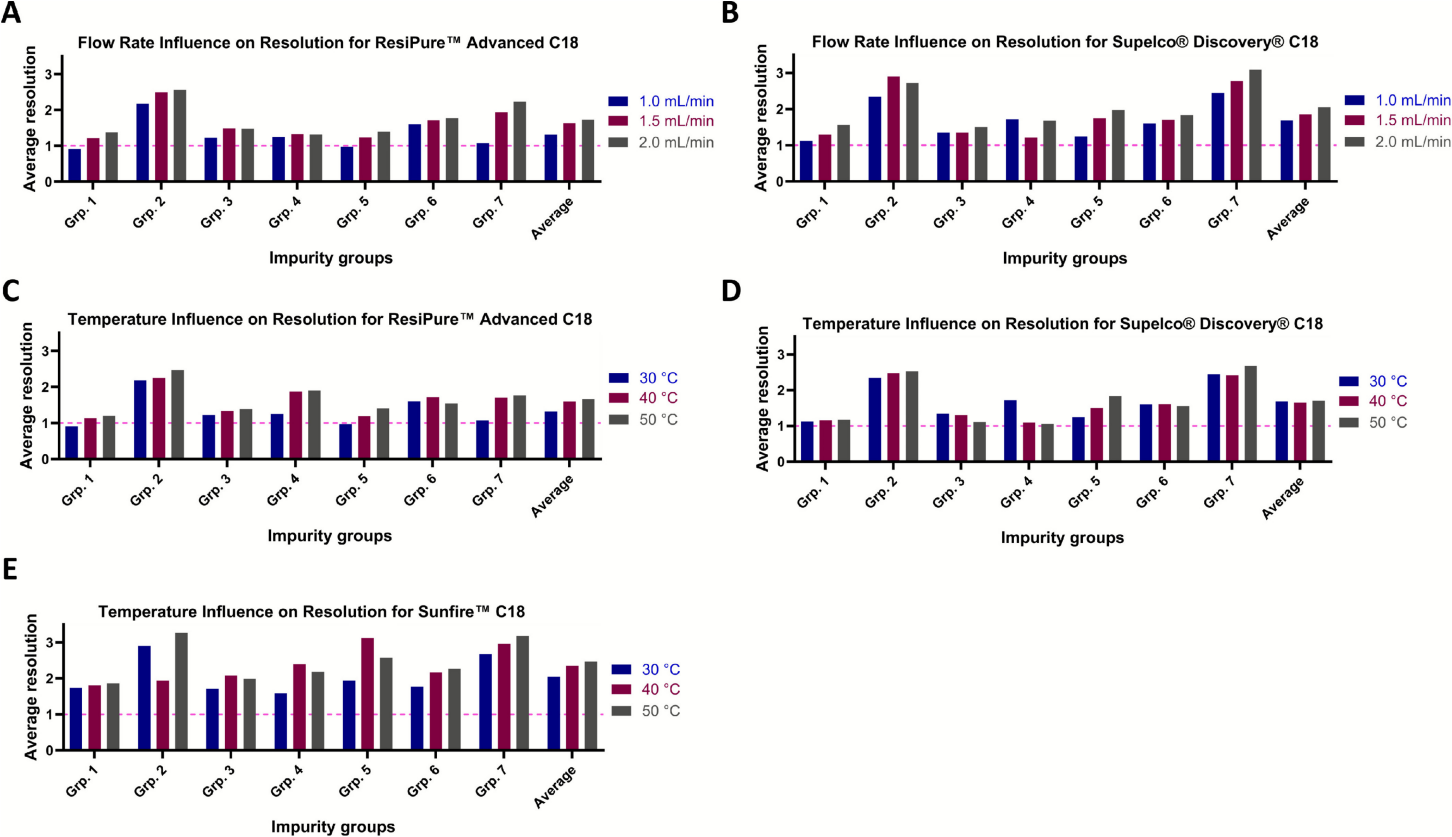
Effect of flow rate and column temperature on the resolution of peptide impurity groups across the three investigated C18 columns. Each bar represents the mean resolution for a single impurity group; ‘average’ denotes the mean resolution across all seven impurity groups. Resolution of 1.0 and therefore a peak overlap of 2.3% are indicated with the dashed pink line. (A, B) Average resolution values for impurity groups measured on the Discovery C18 and ResiPure Advanced C18 columns at flow rates of 1.0, 1.5 and 2.0 mL/min. (C–E) Average resolution values for the same columns as well as the Sunfire C18 at column oven temperatures of 30°C, 40°C and 50°C.

Although the comparison of column type and gradient steepness showed that columns with greater particle sizes like the ResiPure Advanced C18 column can achieve the performance of smaller particle analytical columns, it remained unclear whether this similarity persists when additional chromatographic variables are altered. We therefore next examined the influence of flow rate, column temperature and mobile‐phase modifier to determine whether the separation of synthetic peptides with columns of different particle sizes responds comparably to these key kinetic and selectivity‐driven parameters. For the ResiPure Advanced C18, increasing the flow rate from 1.0 to 1.5 mL/min improved peak shape and resolution, although the number of theoretical plates did not increase. The mean *R*_*s*_ increased from 1.31 to 1.63, although Groups 4 and 7 remained unresolved. This improvement is likely linked to the marked increase in backpressure (48–73 bar), which might shift the column towards a more favourable hydrodynamic regime and reduce mass‐transfer limitations commonly observed at low linear velocities on large‐particle materials [58, 59]. Increasing the flow to 2.0 mL/min (97 bar) further improved separation (mean *R*_*s*_ = 1.73), although the same two groups remained unseparated. In contrast to the resolution trend, theoretical plate numbers did not show a clear flow‐dependent increase. As the number of theoretical plates was used exclusively for comparative assessment of column performance under varying conditions, and conclusions were based on relative trends rather than absolute values, plate numbers are discussed qualitatively and not reported separately. For the Discovery C18, a similar improvement in resolution was observed (mean *R*_*s*_ = 1.69, 1.86 and 2.05 at 1.0, 1.5 and 2.0 mL/min), whereas plate numbers decreased slightly (143,400–134,912). Flow‐dependent resolution profiles are shown in Figure 5A,B (chromatograms can be found in Supporting Information S4.1 and S4.2). For the Sunfire C18, operation above 1.0 mL/min was not feasible due to excessive backpressure. Overall, for our test system and library, the increase of the column oven temperature to 40°C or 50°C showed similar increase in selectivity and resolution as previously reported [23, 41]. For the ResiPure Advanced C18, the mean *R*_*s*_ increased from 1.31 (30°C) to 1.60 (40°C) and 1.67 (50°C), accompanied by a notable rise in mean theoretical plate numbers (113,002, 132,628 and 142,119). The Sunfire C18 displayed comparable behaviour, with mean *R*_*s*_ improving from 2.05 to 2.35 and 2.47, and mean *N* increasing from 159,409 to 177,697 and 180,301 at 30°C, 40°C and 50°C, respectively. The Discovery C18 showed minimal temperature dependence, with *R*_*s*_ remaining effectively constant (*R*_*s*_ = 1.69–1.71) across the tested range. These results may indicate that temperature primarily influences mass‐transfer kinetics for certain stationary phases, whereas others, such as the Discovery C18, are dominated by inherent selectivity. Despite the partially significant increases in resolution, unresolved groups remained identical for each column. As expected, retention times decreased slightly at elevated temperatures. Temperature‐dependent resolution profiles are shown in Figure 5C–E (chromatograms can be found in Supporting Information S5.1–S5.3).

**FIGURE 6:**
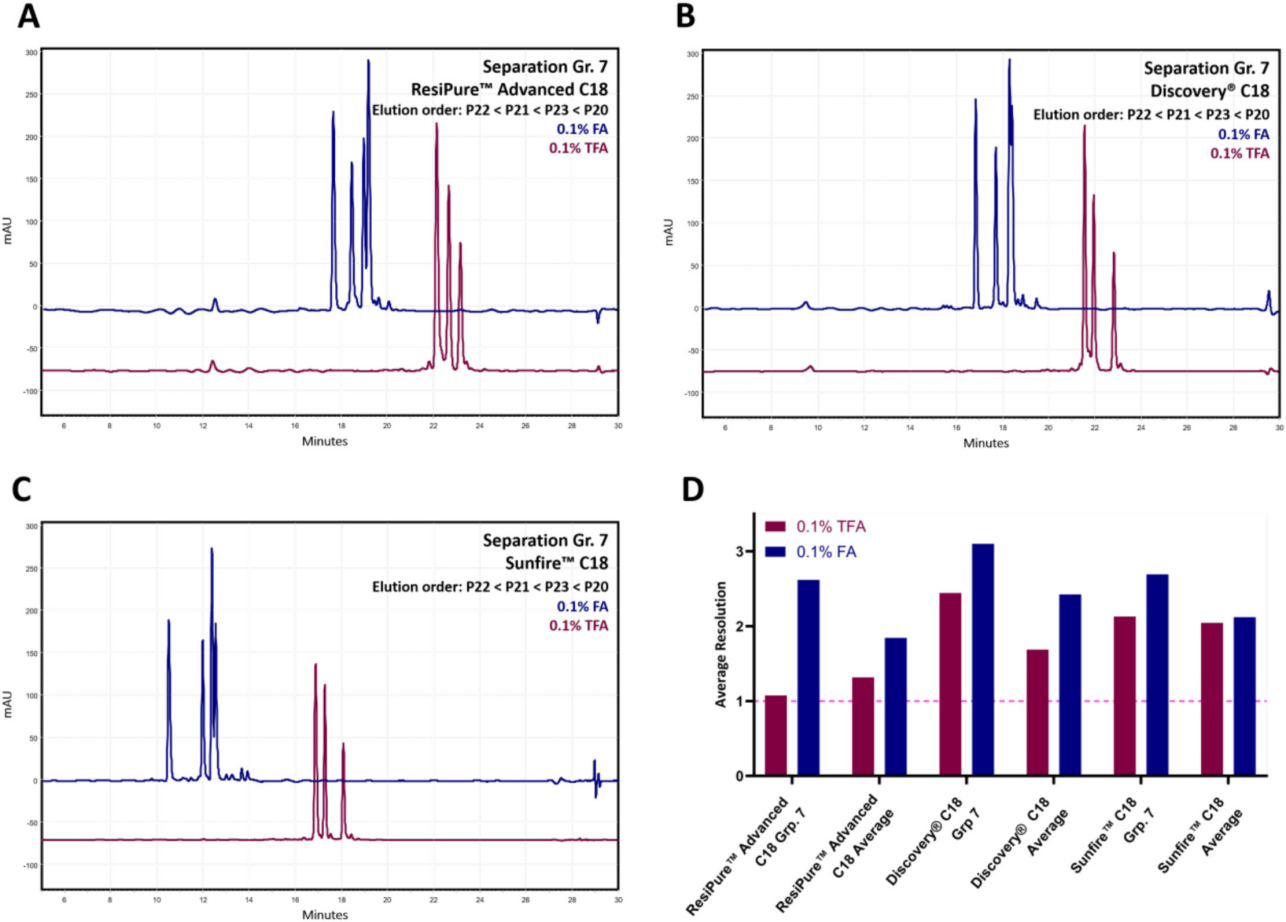
Effect of mobile‐phase modifier on peptide selectivity and resolution. (A–C) Overlaid chromatograms of Impurity Group 7 measured with 0.1% TFA (red) and 0.1% FA (blue) as ion‐pairing modifiers on all columns. Using 0.1% FA the resolution of the complete impurity group was possible. (D) Comparison of average resolution values for the separations obtained with TFA and FA for Impurity Group 7 as well the average across the investigated impurity groups. Resolution of 1.0 and therefore a peak overlap of 2.3% are indicated with the dashed pink line.

Beyond kinetic parameters, we next examined the influence of the mobile‐phase modifier, as ion‐pairing strength and charge interactions are known to exert a far more pronounced effect on peptide selectivity [23, 60, 61]. Replacing TFA with FA in the mobile phase produced column‐dependent changes. For the ResiPure Advanced C18, the mean *R*_*s*_ increased from 1.31 (TFA) to 1.84 (FA) allowing the complete separation of Impurity Group 7. For the Discovery C18, changing the modifier also improved the separation (mean *R*_*s*_ = 1.69 [TFA] to 2.42) with one unresolved group in both cases (Group 7 for TFA and Group 4 for FA). In contrast, the Sunfire C18 showed a strongly polarity‐dependent behaviour, characterized by acetylated peptides which were better resolved under FA, whereas all nonacetylated groups (1, 2, 4 and 5) coeluted with the injection peak. Nevertheless, FA enabled complete resolution of the angiotensin‐derived peptide group (Group 7) for all three columns, which was not achievable with TFA. Representative chromatograms are shown in Figure 6A–C, and the resolution summary is shown in Figure 6D (chromatograms can be found in Figures S6.1–S6.3).

Taken together, these results show that flow rate is the most effective kinetic parameter for enhancing resolution, particularly for columns with larger particle sizes. In contrast, temperature adjustments exert only modest and strongly column‐chemistry‐dependent effects on selectivity. The choice of mobile‐phase modifier remains the most decisive factor, as switching from TFA to FA can markedly improve separation for certain peptide classes while simultaneously causing severe retention losses for hydrophilic, nonacetylated sequences. Overall, these findings highlight that column‐ and sequence‐specific optimization of kinetic parameters and mobile‐phase additives is essential for robust and reliable peptide separations, especially when impurity groups differ only subtly in structure.

### 3.3 Improving Transferability From HPLC to FPLC

To evaluate the transferability of analytical HPLC data to preparative FPLC conditions, P8–P10 and P17–P19 were resynthesized and purified using the scouting‐column ACN elution values as direct predictors. The deviation between predicted and actual elution ranged from −15.3% to −18.1% (mean −17.1%), effectively preventing meaningful purification in the first run and carrying the risk that the target compound coelutes with the injection peak, leading to either incomplete separation or complete product loss. Incorporating system parameters via the developed correction Formula (1) markedly improved accuracy, reducing the mean deviation by 11.8% to a mean of −5.23%, demonstrating a substantial improvement over uncorrected prediction. As the ResiPure Advanced C18 tolerated higher flow rates, we assessed whether higher flow rates yield elution values more comparable to preparative systems. Indeed, using retention data from 1.5 mL/min reduced the average deviation to −3.75%, and 2.0 mL/min further improved it to an average deviation of −3.00%. These values demonstrate that matching flow rates between analytical and preparative modes as much as possible enhances transfer accuracy. Retention times, predicted and experimental ACN percentages, and calculated deviations are summarized in Table 2. Comparable improvements were observed with both analytical HPLC columns (Sunfire C18 and Discovery C18) confirming the general applicability of the developed approach (Supporting Information S7.1 and S7.2). Applying the optimized workflow for the repurification of Impurity Groups 1–4 and 6 yielded > 80% purity for all peptides, with an average purity of 92.9% and mean isolated yield of 32.0%. These results confirm the proof of concept that initial preparative purification of closely related species such as P1, P5 and P6, which differ, for example, only in leucine isomer configuration is achievable using the proposed correction equation. Acetylated peptide groups (3 and 6) showed higher yields (41.4%) and purities (95.5%) than nonacetylated groups (25.3% yield, 91.1% purity), consistent with their higher analytical resolution under TFA. Together, these observations support that analytical HPLC resolution is a reliable predictor of preparative purifiability.

**TABLE 2:** Comparison of predicted, calculated and experimentally determined ACN elution percentages for Peptides P8–P10 and P17–P19 during HPLC‐to‐FPLC method transfer. Retention times were measured on the SiliaChrom ResiPure Advanced C18 at 1.0, 1.5 and 2.0 mL/min. ‘Pred.’ refers to the direct transfer based solely on analytical elution percentages, and ‘calc.’ refers to the corrected values obtained using Formula (1). ‘Det.’ indicates the experimentally observed FPLC elution percentage. ΔACN values denote the deviation between predicted or calculated percentages and the observed ACN at elution. Deviations for the direct transfer (Δ ACN pred. [%]) are indicated in orange; deviations for the transfer with calculated values are indicated in green (Δ ACN calc. [%]). The data illustrate the reduction of systematic transfer error achieved through application of the correction equation.

Flash transfer from HPLC data of SiliaChrom ResiPure Advanced C18 (4.6 × 250 mm, 10 μm)																
	HPLC Rt [min]			FPLC ACN perc. det. [%]	FPLC ACN perc. pred. [%]			Δ ACN pred. [%]			FPLC ACN perc. calc. [%]			Δ ACN calc. [%]		
Sample	1.0 mL/min	1.5 mL/min	2.0 mL/min	30.0 mL/min	1.0 mL/min	1.5 mL/min	2.0 mL/min	1.0 mL/min	1.5 mL/min	2.0 mL/min	1.0 mL/min	1.5 mL/min	2.0 mL/min	1.0 mL/min	1.5 mL/min	2.0 mL/min
P8	20.03	17.39	16.00	17.0	35.1	29.8	27.0	−18.1	−12.8	−10.0	23.2	21.9	21.1	−6.2	−4.9	−4.1
P9	19.76	17.15	15.76	19.2	34.5	29.3	26.5	−15.3	−10.1	−7.3	22.7	21.4	20.6	−3.5	−2.2	−1.4
P10	20.31	17.70	16.29	19.2	35.6	30.4	27.6	−16.4	−11.2	−8.4	23.8	22.5	21.7	−4.6	−3.3	−2.5
P17	19.77	17.16	15.74	17.6	34.5	29.3	26.5	−16.9	−11.7	−8.9	22.7	21.4	20.6	−5.1	−3.8	−3.0
P18	19.52	16.90	15.49	16.3	34.0	28.8	26.0	−17.7	−12.5	−9.7	22.2	20.9	20.1	−5.9	−4.6	−3.8
P19	20.34	17.53	16.09	19.1	35.7	30.1	27.2	−16.6	−11.0	−8.1	23.8	22.2	21.3	−4.7	−3.1	−2.2

## 4 Conclusion and Outlook

Our study systematically investigated key chromatographic parameters influencing peptide separation and their implications for method transfer from analytical HPLC to preparative FPLC. Despite its larger particle size (10 μm), the ResiPure Advanced C18 column demonstrated performance comparable to columns with smaller particles, supporting its suitability for rapid method scouting, purity determination of synthetic peptides and scale‐up. Increasing the flow rate from 1.0 to 1.5 mL/min enhanced efficiency and resolution, whereas a further increase to 2.0 mL/min provided only minor improvement. Increasing the column oven temperature exerted a comparable improvement in separation efficiency to that observed upon increasing the flow rate; however, this effect was strongly dependent on stationary‐phase chemistry. Substitution of TFA with FA induced pronounced, column‐dependent changes in selectivity and resolution, enabling the separation of previously unresolved species and highlighting the potential for greener workflows. The developed correction equation substantially improved the accuracy of HPLC‐to‐FPLC transfer, reducing deviations in elution percentage from approximately 17% to less than 5%. This improvement enabled initial preparative purification of closely related peptide mixtures without iterative reoptimization, achieving average purities exceeding 90% and confirming the practical feasibility of direct analytical‐to‐FPLC transfer. The proposed methodology minimizes solvent and material consumption while providing an adaptable basis for developing green, transferable workflows in peptide manufacturing and analytical quality control.

## Author Contributions

A.S. designed the study, developed the correction equation, carried out proof‐of‐concept experiments, synthesized and purified peptides, performed the HPLC and FPLC experiments, evaluated the data, prepared the figures and wrote the manuscript. V.E. developed the impurity library and synthesized and purified peptides. B.N., V.B. and F.B. helped with data interpretation and provided chemical knowledge about column chemistry. C.S. provided resources and supervision and wrote the manuscript. All authors read, reviewed and approved the final version of the manuscript.

## Conflicts of Interest

B.N., V.B. and F.B. are employed by Zeochem Silica Materials/SilliCycle Inc., which have developed and sell SiliCycle columns. The other authors declare no conflicts of interest.

## Supporting information


**Figure S1:1** Peptide purity after synthesis and purification determined by HPLC‐UV.
**Figure S1:2** RP‐LC chromatograms and mass spectra of P1‐P23.
**Figure S2:1** RP‐LC chromatograms and mass spectra of impurity groups 1–7 measured with the Sunfire C18.
**Figure S2:2** RP‐LC chromatograms and mass spectra of impurity groups 1–7 measured with the Supelco Discovery C18.
**Figure S2:3** RP‐LC chromatograms and mass spectra of impurity groups 1–7 measured with the ResiPure Advanced C18.
**Figure S2:4** RP‐LC chromatograms and mass spectra of impurity groups 1–7 measured with the InnoPeptide C18.
**Figure S2:5** RP‐LC chromatograms and mass spectra of impurity groups 1–7 measured with the SiliaChrom Plus HPLC C18.
**Figure S3:1** RP‐HPLC‐UV chromatograms of groups 1–7 with varying gradient steepness measured on the ResiPure Advanced C18.
**Figure S3:2** RP‐HPLC‐UV chromatograms of groups 1–7 with varying gradient steepness measured on the Supelco Discovery C18.
**Figure S3:3** RP‐HPLC‐UV chromatograms of groups 1–7 with varying gradient steepness measured on the Sunfire C18.
**Figure S4:1** RP‐HPLC‐UV chromatograms of groups 1–7 with varying flow rates measured on the ResiPure Advanced C18.
**Figure S4:2** RP‐HPLC‐UV chromatograms of groups 1–7 with varying flow rates measured on the Supelco Discovery C18.
**Figure S5:1** RP‐HPLC‐UV chromatograms of groups 1–7 with varying temperatures measured on the ResiPure Advanced C18.
**Figure S5:2** RP‐HPLC‐UV chromatograms of groups 1–7 with varying temperatures measured on the Supelco Discovery C18.
**Figure S5:3** RP‐HPLC‐UV chromatograms of groups 1–7 with varying temperatures measured on the Sunfire C18.
**Figure S6:1** RP‐HPLC‐UV chromatograms of groups 1–7 with TFA and FA as modifiers measured on the ResiPure Advanced C18.
**Figure S6:2** RP‐HPLC‐UV chromatograms of groups 1–7 with TFA and FA as modifiers measured on the Supelco Discovery C18.
**Figure S6:3** RP‐HPLC‐UV chromatograms of groups 1–7 with TFA and FA as modifiers measured on the Sunfire C18.
**Table S7:1** Comparison of predicted, calculated, and experimentally determined ACN elution percentages for peptides P8, P9, P10, P17, P18 and P19 during HPLC‐to‐FPLC method transfer for the Supelco Discovery C18. Retention times were measured on the Supelco Discovery C18 at 1.0, 1.5, and 2.0 mL/min. “Pred.” refers to the direct transfer based solely on analytical elution percentages, and “Calc.” refers to the corrected values obtained using Formula 1. “Det.” indicates the experimentally observed FPLC elution percentage. ΔACN values denote the deviation between predicted or calculated percentages and the observed ACN at elution. Deviations for the direct transfer (Δ ACN Pred. [%]) is indicated in orange, deviations for the transfer with calculated values are indicated in green (Δ ACN Calc. [%]). The data illustrate the reduction of systematic transfer error achieved through application of the correction model.
**Table S7:2** Comparison of predicted, calculated, and experimentally determined ACN elution percentages for peptides P8, P9, P10, P17, P18 and P19 during HPLC‐to‐FPLC method transfer for the Supelco Discovery C18. Retention times were measured on the Sunfire C18 at 1.0 mL/min. “Pred.” refers to the direct transfer based solely on analytical elution percentages, and “Calc.” refers to the corrected values obtained using Formula 1. “Det.” indicates the experimentally observed FPLC elution percentage. ΔACN values denote the deviation between predicted or calculated percentages and the observed ACN at elution. Deviations for the direct transfer (Δ ACN Pred. [%]) is indicated in orange, deviations for the transfer with calculated values are indicated in green (Δ ACN Calc. [%]). The data illustrate the reduction of systematic transfer error achieved through application of the correction model.

## Data Availability

Exemplary data for all experiments are available in the publication or the Supporting Information. Raw data are available from the authors upon request.

## References

[1] K. Fosgerau and T. Hoffmann , “Peptide Therapeutics: Current Status and Future Directions,” Drug Discovery Today 20, no. 1 (2015): 122–128. 25450771 10.1016/j.drudis.2014.10.003

[2] J. L. Lau and M. K. Dunn , “Therapeutic Peptides: Historical Perspectives, Current Development Trends, and Future Directions,” Bioorganic & Medicinal Chemistry 26, no. 10 (2018): 2700–2707. 28720325 10.1016/j.bmc.2017.06.052

[3] S. Dhillon , “Semaglutide: First Global Approval,” Drugs 78, no. 2 (2018): 275–284. 29363040 10.1007/s40265-018-0871-0

[4] Y. Y. Syed , “Tirzepatide: First Approval,” Drugs 82, no. 11 (2022): 1213–1220. 35830001 10.1007/s40265-022-01746-8

[5] S. Dhillon and S. J. Keam , “Bremelanotide: First Approval,” Drugs 79, no. 14 (2019): 1599–1606. 31429064 10.1007/s40265-019-01187-w

[6] A. Markham , “Setmelanotide: First Approval,” Drugs 81, no. 3 (2021): 397–403. 33638809 10.1007/s40265-021-01470-9

[7] V. D'Aloisio , P. Dognini , G. A. Hutcheon , and C. R. Coxon , “PepTherDia: Database and Structural Composition Analysis of Approved Peptide Therapeutics and Diagnostics,” Drug Discovery Today 26, no. 6 (2021): 1409–1419. 33647438 10.1016/j.drudis.2021.02.019

[8] A. A. Kaspar and J. M. Reichert , “Future Directions for Peptide Therapeutics Development,” Drug Discovery Today 18, no. 17–18 (2013): 807–817. 23726889 10.1016/j.drudis.2013.05.011

[9] D. J. Craik , D. P. Fairlie , S. Liras , and D. Price , “The Future of Peptide‐Based Drugs,” Chemical Biology & Drug Design 81, no. 1 (2013): 136–147. 23253135 10.1111/cbdd.12055

[10] J. M. Palomo , “Solid‐Phase Peptide Synthesis: An Overview Focused on the Preparation of Biologically Relevant Peptides,” RSC Advances 4, no. 62 (2014): 32658–32672.

[11] R. B. Merrifield , “Solid Phase Peptide Synthesis. I. The Synthesis of a Tetrapeptide,” Journal of the American Chemical Society 85, no. 14 (1963): 2149–2154.

[12] L. A. Carpino and G. Y. Han , “9‐Fluorenylmethoxycarbonyl Function, a New Base‐Sensitive Amino‐Protecting Group,” Journal of the American Chemical Society 92, no. 19 (1970): 5748–5749.

[13] T. Geiger and S. Clarke , “Deamidation, Isomerization, and Racemization at Asparaginyl and Aspartyl Residues in Peptides. Succinimide‐Linked Reactions That Contribute to Protein Degradation,” Journal of Biological Chemistry 262, no. 2 (1987): 785–794. 3805008

[14] H. Yang and R. A. Zubarev , “Mass Spectrometric Analysis of Asparagine Deamidation and Aspartate Isomerization in Polypeptides,” Electrophoresis 31, no. 11 (2010): 1764–1772. 20446295 10.1002/elps.201000027 PMC3104603

[15] M. D'Hondt , N. Bracke , L. Taevernier , et al., “Related Impurities in Peptide Medicines,” Journal of Pharmaceutical and Biomedical Analysis 101 (2014): 2–30, 10.1016/j.jpba.2014.06.012. 25044089

[16] K. Kato , T. Nakayoshi , E. Kurimoto , and A. Oda , “Mechanisms of Deamidation of Asparagine Residues and Effects of Main‐Chain Conformation on Activation Energy,” International Journal of Molecular Sciences 21, no. 19 (2020): 7035–7048, 10.3390/ijms21197035. 32987875 PMC7582646

[17] N. P. Sargaeva , A. A. Goloborodko , P. B. O'Connor , E. Moskovets , and M. V. Gorshkov , “Sequence‐Specific Predictive Chromatography to Assist Mass Spectrometric Analysis of Asparagine Deamidation and Aspartate Isomerization in Peptides,” Electrophoresis 32, no. 15 (2011): 1962–1969, 10.1002/elps.201000507. 21557257

[18] M. R. Nilsson , M. Driscoll , and D. P. Raleigh , “Low Levels of Asparagine Deamidation Can Have a Dramatic Effect on Aggregation of Amyloidogenic Peptides: Implications for the Study of Amyloid Formation,” Protein Science 11, no. 2 (2002): 342–349. 11790844 10.1110/ps.48702 PMC2373442

[19] J. W. de Beukelaar , J. W. Gratama , P. A. S. Smitt , et al., “The Impact of Impurities in Synthetic Peptides on the Outcome of T‐Cell Stimulation Assays,” Rapid Communications in Mass Spectrometry 21, no. 7 (2007): 1282–1288. 17340558 10.1002/rcm.2958

[20] D. J. Adams , T. G. Nemkov , J. P. Mayer , W. M. Old , and M. H. B. Stowell , “Identification of the Primary Peptide Contaminant That Inhibits Fibrillation and Toxicity in Synthetic Amyloid‐β42,” PLoS ONE 12, no. 8 (2017): e0182804, 10.1371/journal.pone.0182804. 28792968 PMC5549942

[21] J. R. Currier , L. M. Galley , H. Wenschuh , et al., “Peptide Impurities in Commercial Synthetic Peptides and Their Implications for Vaccine Trial Assessment,” Clinical and Vaccine Immunology 15, no. 2 (2008): 267–276, 10.1128/CVI.00284-07. 18077621 PMC2238048

[22] C. de Luca , G. Lievore , D. Bozza , et al., “Downstream Processing of Therapeutic Peptides by Means of Preparative Liquid Chromatography,” Molecules 26, no. 15 (2021): 4688–4707, 10.3390/molecules26154688. 34361839 PMC8348516

[23] C. T. Mant , Y. Chen , Z. Yan , et al., “HPLC Analysis and Purification of Peptides,” Methods in Molecular Biology 386 (2007): 3–55, 10.1007/978-1-59745-430-8_1. 18604941 PMC7119934

[24] Y. Yang , R. I. Boysen , and M. T. Hearn , “Hydrophilic Interaction Chromatography Coupled to Electrospray Mass Spectrometry for the Separation of Peptides and Protein Digests,” Journal of Chromatography A 1216, no. 29 (2009): 5518–5524. 19535084 10.1016/j.chroma.2009.05.085

[25] A. J. Alpert , “Hydrophilic‐Interaction Chromatography for the Separation of Peptides, Nucleic Acids and Other Polar Compounds,” Journal of Chromatography A 499 (1990): 177–196. 10.1016/s0021-9673(00)96972-3 2324207

[26] S. Janvier , E. de Sutter , E. Wynendaele , B. de Spiegeleer , C. Vanhee , and E. Deconinck , “Analysis of Illegal Peptide Drugs via HILIC‐DAD‐MS,” Talanta 174 (2017): 562–571. 28738623 10.1016/j.talanta.2017.06.034

[27] R. Simon , Q. Enjalbert , J. Biarc , J. Lemoine , and A. Salvador , “Evaluation of Hydrophilic Interaction Chromatography (HILIC) Versus C18 Reversed‐Phase Chromatography for Targeted Quantification of Peptides by Mass Spectrometry,” Journal of Chromatography A 1264 (2012): 31–39. 23073287 10.1016/j.chroma.2012.09.059

[28] A. Periat , I. S. Krull , and D. Guillarme , “Applications of Hydrophilic Interaction Chromatography to Amino Acids, Peptides, and Proteins,” Journal of Separation Science 38, no. 3 (2015): 357–367, 10.1002/jssc.201400969. 25413716

[29] J. Zheng , J. Pinkston , P. Zoutendam , and L. Taylor , “Feasibility of Supercritical Fluid Chromatography/Mass Spectrometry of Polypeptides With up to 40‐Mers,” Analytical Chemistry 78, no. 5 (2006): 1535–1545. 16503605 10.1021/ac052025s

[30] D. Tognarelli , A. Tsukamoto , J. Caldwell , and W. Caldwell , “Rapid Peptide Separation by Supercritical Fluid Chromatography,” Bioanalysis 2, no. 1 (2010): 5–7. 21083112 10.4155/bio.09.165

[31] J. Neumann , S. Schmidtsdorff , A. H. Schmidt , and M. K. Parr , “Retention Modeling of Therapeutic Peptides in Sub‐/Supercritical Fluid Chromatography,” Separation Science Plus 7, no. 5 (2024): 2300239. 10.1002/jssc.202201007 36601991

[32] J. Neumann , S. Schmidtsdorff , A. H. Schmidt , and M. K. Parr , “Controlling the Elution Order of Insulin and Its Analogs in Sub‐/Supercritical Fluid Chromatography Using Methanesulfonic Acid and 18‐Crown‐6 as Mobile Phase Additives,” Journal of Separation Science 46, no. 22 (2023): 2300520. 10.1002/jssc.202300520 37775313

[33] K. Kupnik , Ž. Knez , M. Primožič , and M. Leitgeb , “Separation of Amino Acids and Peptides With Supercritical Fluids Chromatography,” Separation and Purification Reviews 52, no. 1 (2023): 58–74.

[34] V. Erckes and C. Steuer , “A Story of Peptides, Lipophilicity and Chromatography–Back and Forth in Time,” RSC Medicinal Chemistry 13, no. 6 (2022): 676–687. 35800203 10.1039/d2md00027j PMC9215158

[35] J. Field , J. B. Bruce , S. Buckenmaier , et al., “Method Development for Reversed‐Phase Separations of Peptides: A Rational Screening Strategy for Column and Mobile Phase Combinations With Complementary Selectivity,” LCGC Europe 35 (2022): 440–449.

[36] M. C. Garcia , “The Effect of the Mobile Phase Additives on Sensitivity in the Analysis of Peptides and Proteins by High‐Performance Liquid Chromatography‐Electrospray Mass Spectrometry,” Journal of Chromatography. B, Analytical Technologies in the Biomedical and Life Sciences 825, no. 2 (2005): 111–123, 10.1016/j.jchromb.2005.03.041. 16213445

[37] S. Fekete , J. L. Veuthey , and D. Guillarme , “New Trends in Reversed‐Phase Liquid Chromatographic Separations of Therapeutic Peptides and Proteins: Theory and Applications,” Journal of Pharmaceutical and Biomedical Analysis 69 (2012): 9–27, 10.1016/j.jpba.2012.03.024. 22475515

[38] M. Y. Cheung , J. Bruce , M. R. Euerby , J. K. Field , and P. Petersson , “Investigation Into Reversed‐Phase Chromatography Peptide Separation Systems Part V: Establishment of a Screening Strategy for Development of Methods for Assessment of Pharmaceutical Peptides' Purity,” Journal of Chromatography A 1668 (2022): 462888. 35231862 10.1016/j.chroma.2022.462888

[39] N. M. González‐López , D. S. Insuasty‐Cepeda , K. A. Huertas‐Ortiz , et al., “Gradient Retention Factor Concept Applied to Method Development for Peptide Analysis by Means of RP‐HPLC,” ACS Omega 7, no. 49 (2022): 44817–44824. 36530233 10.1021/acsomega.2c04907 PMC9753532

[40] V. D'Aloisio , A. Schofield , D. A. Kendall , G. A. Hutcheon , and C. R. Coxon , “The Development and Optimisation of an HPLC‐Based In Vitro Serum Stability Assay for a Calcitonin Gene‐Related Peptide Receptor Antagonist Peptide,” Journal of Peptide Science 30, no. 2 (2024): e3539, 10.1002/psc.3539. 37605343

[41] O. Krokhin , “Peptide Retention Prediction in Reversed‐Phase Chromatography: Proteomic Applications,” Expert Review of Proteomics 9, no. 1 (2012): 1–4. 22292816 10.1586/epr.11.79

[42] K. Sikora , M. Jaśkiewicz , D. Neubauer , D. Migoń , and W. Kamysz , “The Role of Counter‐Ions in Peptides—An Overview,” Pharmaceuticals 13, no. 12 (2020): 442. 33287352 10.3390/ph13120442 PMC7761850

[43] J. Cornish , K. Callon , C.‐X. Lin , et al., “Trifluoroacetate, A Contaminant in Purified Proteins, Inhibits Proliferation of Osteoblasts and Chondrocytes,” American Journal of Physiology—Endocrinology and Metabolism 277, no. 5 (1999): E779–E783. 10.1152/ajpendo.1999.277.5.E779 10567002

[44] K. Sikora , M. Jaśkiewicz , D. Neubauer , et al., “Counter‐Ion Effect on Antistaphylococcal Activity and Cytotoxicity of Selected Antimicrobial Peptides,” Amino Acids 50, no. 5 (2018): 609–619. 29307075 10.1007/s00726-017-2536-9 PMC5917001

[45] C. Ardino , F. Sannio , C. Pasero , et al., “The Impact of Counterions in Biological Activity: Case Study of Antibacterial Alkylguanidino Ureas,” Molecular Diversity 27, no. 3 (2023): 1489–1499. 36036302 10.1007/s11030-022-10505-6 PMC9421121

[46] H. P. Arp , A. Gredelj , J. Gluge , M. Scheringer , and I. T. Cousins , “The Global Threat From the Irreversible Accumulation of Trifluoroacetic Acid (TFA),” Environmental Science & Technology 58, no. 45 (2024): 19925–19935. 39475534 10.1021/acs.est.4c06189 PMC11562725

[47] K. R. Solomon , G. J. Velders , S. R. Wilson , et al., “Sources, Fates, Toxicity, and Risks of Trifluoroacetic Acid and Its Salts: Relevance to Substances Regulated Under the Montreal and Kyoto Protocols,” Journal of Toxicology and Environmental Health, Part B: Critical Reviews 19, no. 7 (2016): 289–304. 27351319 10.1080/10937404.2016.1175981

[48] M. d. l. A. Garavagno , R. Holland , M. A. H. Khan , A. J. Orr‐Ewing , and D. E. Shallcross , “Trifluoroacetic Acid: Toxicity, Sources, Sinks and Future Prospects,” Sustainability 16, no. 6 (2024): 2382.

[49] V. Erckes , A. Streuli , L. Chamera Rendueles , S. D. Krämer , and C. Steuer , “Towards a Consensus for the Analysis and Exchange of TFA as a Counterion in Synthetic Peptides and Its Influence on Membrane Permeation,” Pharmaceuticals 18, no. 8 (2025): 1163. 40872554 10.3390/ph18081163 PMC12389442

[50] K. K. Sorensen , N. K. Mishra , M. P. Paprocki , A. Mehrotra , and K. J. Jensen , “High‐Performance Reversed‐Phase Flash Chromatography Purification of Peptides and Chemically Modified Insulins,” ChemBioChem 22, no. 10 (2021): 1818–1822, 10.1002/cbic.202000826. 33443297

[51] E. Denton and A. Mehrotra , “Tips and Tricks in Reversed‐Phase Flash Chromatography for Peptide Purification,” Methods in Molecular Biology 2931 (2025): 187–216. 40531458 10.1007/978-1-0716-4562-8_15

[52] L. A. Lawton , J. McElhiney , and C. Edwards , “Purification of Closely Eluting Hydrophobic Microcystins (Peptide Cyanotoxins) by Normal‐Phase and Reversed‐Phase Flash Chromatography,” Journal of Chromatography A 848, no. 1–2 (1999): 515–522.

[53] F. Gritti and G. Guiochon , “On the Extra‐Column Band‐Broadening Contributions of Modern, Very High Pressure Liquid Chromatographs Using 2.1 mm ID Columns Packed With Sub‐2 μm Particles,” Journal of Chromatography A 1217, no. 49 (2010): 7677–7689. 21044782 10.1016/j.chroma.2010.10.016

[54] T. Fornstedt , P. Forssén , and D. Westerlund , “Basic HPLC Theory and Definitions: Retention, Thermodynamics, Selectivity, Zone Spreading, Kinetics, and Resolution,” Analytical Separation Science Set 5, no. Part 2 (2015): 1–22.

[55] L. R. Snyder , J. J. Kirkland , and J. W. Dolan , Introduction to Modern Liquid Chromatography (John Wiley & Sons, 2011).

[56] M. J. Santos , J. A. Teixeira , and L. R. Rodrigues , “Fractionation of the Major Whey Proteins and Isolation of β‐Lactoglobulin Variants by Anion Exchange Chromatography,” Separation and Purification Technology 90 (2012): 133–139.

[57] V. Erckes , M. Hilleke , C. Isert , and C. Steuer , “PICKAPEP: An Application for Parameter Calculation and Visualization of Cyclized and Modified Peptidomimetics,” Journal of Peptide Science 30, no. 12 (2024): e3646. 39085168 10.1002/psc.3646

[58] J. van Deemter , F. Zuiderweg , and A. v. Klinkenberg , “Longitudinal Diffusion and Resistance to Mass Transfer as Causes of Nonideality in Chromatography,” Chemical Engineering Science 50, no. 24 (1995): 3869–3882.

[59] G. Guiochon , D. G. Shirazi , and A. Felinger , Fundamentals of Preparative and Nonlinear Chromatography (Academic Press, 2006).

[60] M. Shibue , C. Mant , and R. Hodges , “Effect of Anionic Ion‐Pairing Reagent Hydrophobicity on Selectivity of Peptide Separations by Reversed‐Phase Liquid Chromatography,” Journal of Chromatography A 1080, no. 1 (2005): 68–75. 16013616 10.1016/j.chroma.2005.03.035 PMC2744697

[61] D. Gussakovsky , G. Anderson , V. Spicer , and O. V. Krokhin , “Peptide Separation Selectivity in Proteomics LC‐MS Experiments: Comparison of Formic and Mixed Formic/Heptafluorobutyric Acids Ion‐Pairing Modifiers,” Journal of Separation Science 43, no. 20 (2020): 3830–3839. 32818315 10.1002/jssc.202000578

