# Supplementary material for: Improvement of Analysis and Transferability in Peptide Purification: From HPLC to FPLC and Back Again
Source: J Pept Sci. 2026 Feb 10;32(3):e70090. doi: 10.1002/psc.70090 (PMC12890753; doi:10.1002/psc.70090)
Supplement: Supplementary file 1 — Figure S1:1 Peptide purity after synthesis and purification determined by HPLC‐UV. Figure S1:2 RP‐LC chromatograms and mass spectra of P1‐P23. Figure S2:1 RP‐LC chromatograms and mass spectra of impurity groups 1–7 measured with the Sunfire C18. Figure S2:2 RP‐LC chromatograms and mass spectra of impurity groups 1–7 measured with the Supelco Discovery C18. Figure S2:3 RP‐LC chromatograms and mass spectra of impurity groups 1–7 measured with the ResiPure Advanced C18. Figure S2:4 RP‐LC chromatograms and mass spectra of impurity groups 1–7 measured with the InnoPeptide C18. Figure S2:5 RP‐LC chromatograms and mass spectra of impurity groups 1–7 measured with the SiliaChrom Plus HPLC C18. Figure S3:1 RP‐HPLC‐UV chromatograms of groups 1–7 with varying gradient steepness measured on the ResiPure Advanced C18. Figure S3:2 RP‐HPLC‐UV chromatograms of groups 1–7 with varying gradient steepness measured on the Supelco Discovery C18. Figure S3:3 RP‐HPLC‐UV chromatograms of groups 1–7 with varying gradient steepness measured on the Sunfire C18. Figure S4:1 RP‐HPLC‐UV chromatograms of groups 1–7 with varying flow rates measured on the ResiPure Advanced C18. Figure S4:2 RP‐HPLC‐UV chromatograms of groups 1–7 with varying flow rates measured on the Supelco Discovery C18. Figure S5:1 RP‐HPLC‐UV chromatograms of groups 1–7 with varying temperatures measured on the ResiPure Advanced C18. Figure S5:2 RP‐HPLC‐UV chromatograms of groups 1–7 with varying temperatures measured on the Supelco Discovery C18. Figure S5:3 RP‐HPLC‐UV chromatograms of groups 1–7 with varying temperatures measured on the Sunfire C18. Figure S6:1 RP‐HPLC‐UV chromatograms of groups 1–7 with TFA and FA as modifiers measured on the ResiPure Advanced C18. Figure S6:2 RP‐HPLC‐UV chromatograms of groups 1–7 with TFA and FA as modifiers measured on the Supelco Discovery C18. Figure S6:3 RP‐HPLC‐UV chromatograms of groups 1–7 with TFA and FA as modifiers measured on the Sunfire C18. Table S7:1 Comparison of predicted, [file PSC-32-e70090-s001.docx]

**Supporting Information**

**Larger particle size for analysis and transferability in peptide purification: From HPLC to FPLC and back again**

Alessandro Streuli.^1^; Vanessa Erckes^1^; Brunello Nardone^2^; Vincent Bedard^2^; François Beland^2^; Christian Steuer*^1^

^1^ ETH Zurich, Institute of Pharmaceutical Sciences, Laboratory of Pharmaceutical Analytics, Zurich, Switzerland

^2^ Zeochem Silica Materials, 2500, Boul. du Parc-Technologique, Quebec (Quebec) G1P 4S6, Canada

* Corresponding author: [christian.steuer@pharma.ethz.ch](mailto:christian.steuer@pharma.ethz.ch)

**ORCID**

AS:0000-0002-0025-8023

VE: 0000-0002-9650-4160

CS:0000-0002-6102-3367

**Table of Contents**

Contents

[SI-1: Peptide Synthesis, Purification and Characterization 3](#_Toc215603800)

[SI-2: Individual chromatograms to test the effect of the column choice on the separation efficacy 10](#_Toc215603801)

[SI-3: Individual chromatograms to test the effect of the gradient steepness on the separation efficacy 15](#_Toc215603802)

[SI-4: Individual chromatograms to test the effect of the flow rate on the separation efficacy 26](#_Toc215603803)

[SI-5: Individual chromatograms to test the effect of the temperature on the separation efficacy 33](#_Toc215603804)

[SI-6: Individual chromatograms to test the effect of the modifier on the separation efficacy 44](#_Toc215603805)

[SI-7: Improving transferability from HPLC to FPLC: Sunfire™ C18 and Discovery® C18 49](#_Toc215603806)

# SI-1: Peptide Synthesis, Purification and Characterization

All peptides were produced via standard Fmoc solid-phase peptide synthesis (SPPS) on a PurePrep Chorus peptide synthesizer (Gyros Protein Technologies, USA), employing either Rink amide MBHA resin or the appropriate preloaded Wang resin. Syntheses followed conventional procedures, using dimethylformamide (DMF; EMPLURA Supelco, Merck) as both solvent and swelling medium. Fmoc removal was carried out with a 20% (v/v) solution of pyrrolidine (99+%, Thermo Scientific) in DMF, and peptide coupling was achieved using 5 equivalents of O-(1H-6-chlorobenzotriazol-1-yl)-1,1,3,3-tetramethyluronium hexafluorophosphate (HCTU; Gyros Protein Technologies) combined with 10 equivalents of 2,6-dimethylmorpholine (NMM; 99+%, Thermo Scientific) and 5 equivalents of Fmoc-protected amino acids (ProteinTechnologies, United Kingdoms). Cleavage and global deprotection were performed using a mixture of 95% TFA (99.5%, Apollo Scientific), 2.5% triisopropylsilane (TIS; Sigma Aldrich), and 2.5% H₂O (v/v/v). Crude peptides were precipitated and washed twice with cold ether (Emsure Supelco, Ph. Eur., Merck).

Peptide identity and mass accuracy were verified by LC–MS using a Waters Acquity™ UPLC instrument (Milford, MA, USA) coupled to an LTQ-XL linear ion trap mass spectrometer equipped with a heated ESI II source (Thermo Scientific, San Jose, CA, USA). The chromatographic separation employed a mobile phase consisting of 0.1% formic acid (FA) in water (eluent A) and 0.1% FA in acetonitrile (ACN; OPTIMA LC–MS grade, Fisher Chemicals) (eluent B). Analyses were conducted on a Zorbax Eclipse Plus C18 reversed-phase column (2.1 × 50 mm, 1.8 µm; Agilent Technologies, Santa Clara, CA, USA) at ambient temperature, with the autosampler maintained at 10 °C and an injection volume of 10 µL. The gradient was used with a 0.5 mL/min flow and consisted of: 0–2 min, 5% B; 2–10 min, linear increase to 70% B; 10–12 min, ramp to 90% B; 12–15 min, hold at 90% B; 15–15.5 min, return to 5% B; 15.5–20 min, re-equilibration at 5% B. The ESI source was operated without additional heating, using sheath and auxiliary gas settings of 34 and 11 arbitrary units, respectively. The spray voltage was 5.00 kV, the ion transfer capillary temperature was maintained at 275 °C, and the capillary and tube-lens voltages were set to 31 V and 80 V. Mass spectrometric detection was performed in positive ion mode with full-scan acquisition over m/z 250–2000 at normal scan speed. Data processing was carried out using Xcalibur software (Thermo Scientific, Version 4.4.16.14).

Peptide purification was performed by C18 reversed-phase flash chromatography on a puriFlash XS520Plus system (Interchim). Crude peptides were dissolved in the smallest feasible volume of DMSO and manually applied to SiliaSep™ PREMIUM C18 flash cartridges (12 g, 25 µm, 90 Å; Zeochem Silica Materials, Quebec, Canada). Water and acetonitrile containing 0.2% TFA served as the mobile phases. The purification gradients were initiated and terminated at ACN concentrations 10% above the predicted analytical elution percentage to ensure complete recovery of the target species. Prior to each run, cartridges were equilibrated for 5 column volumes (CV) at the starting conditions, which were maintained for an additional 5 CV following sample injection. Standard gradients were executed over 30 CV, whereas the separation of defined impurity groups required an extended gradient of 45 CV. Fraction and product collection were performed in a targeted manner based on the UV signal at 214 nm, with collection triggered once the absorbance exceeded a predefined threshold. Upon completion of the main gradient, the ACN content was increased to 100% over 5 CV and held for another 5 CV to flush strongly retained components. Collected fractions were subsequently lyophilized.

For purity control 0.1 - 0.2 mg/mL solutions of the dried peptides was performed using a VWR ELITE Lachrome Series LC with an UV detector. For chromatographic separation, a Sunfire™ C18 (3.5 μm, 3.0 × 150 mm; Waters Corporation, Milford, MA, USA) was used. The mobile phase for gradient elution consisted of 0.1% TFA in H_2_O (eluent A) and 0.1% TFA in ACN (HPLC gradient grade, ≥ 99.9%, Sigma Aldrich) (eluent B). The used gradient with a flow rate of 1.0 mL/min was as followed: 0-5 min at 5% B, 5-50.0 min to 95% B, 50.0-55.0 min at 95% B, 55.0-56.0 min to 5% B, 56.0-65.0 min reequilibration with 5% B. The column oven was set to 25 °C and the injection volume to 10 µL. UV detection was performed at 214 nm wavelength with a sampling period of 200 ms and a response time of 1 s. For data evaluation, the OpenLab software (Version A. 04.08 - Agilent Technologies, Santa Clara, CA, USA) was used.


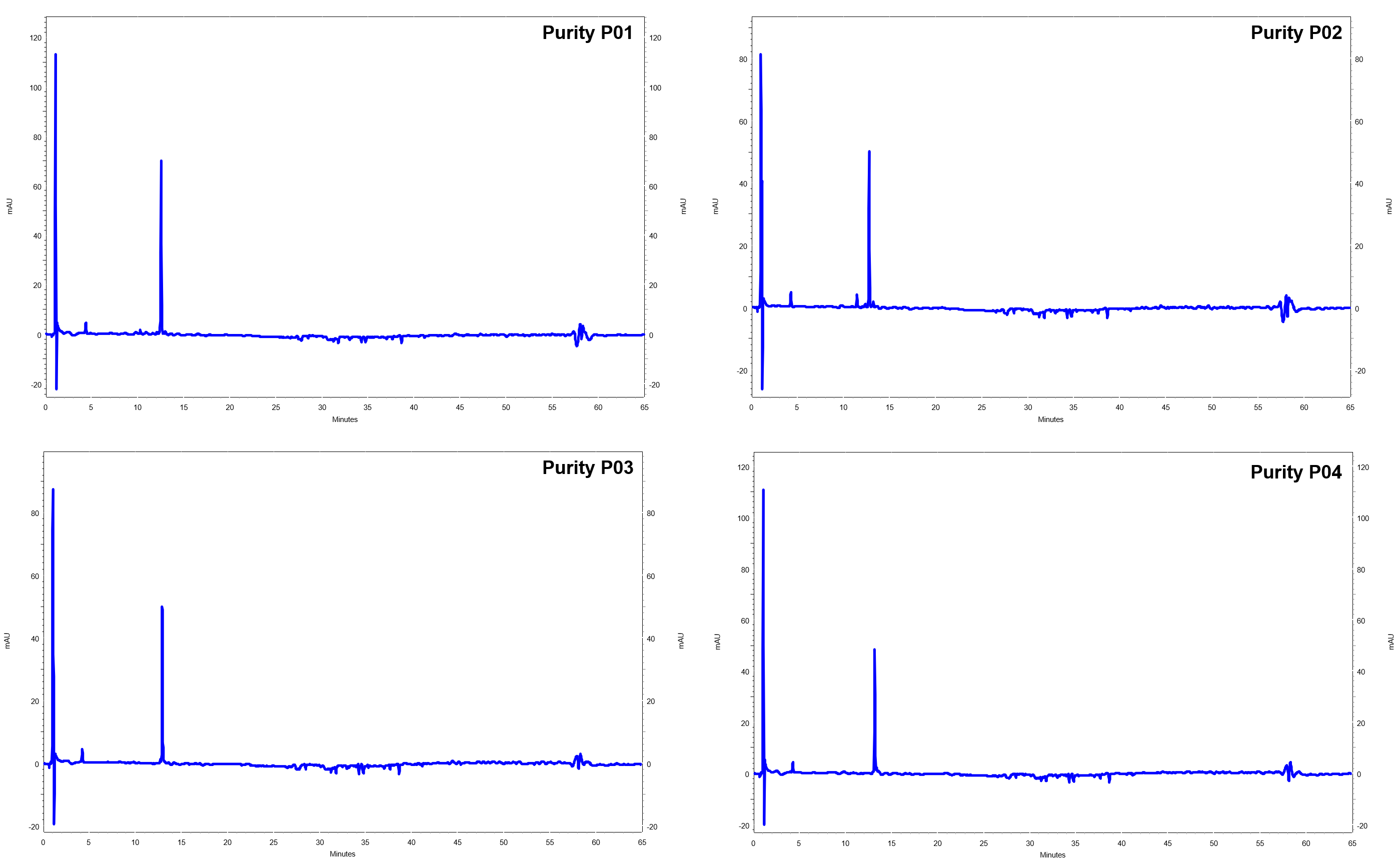


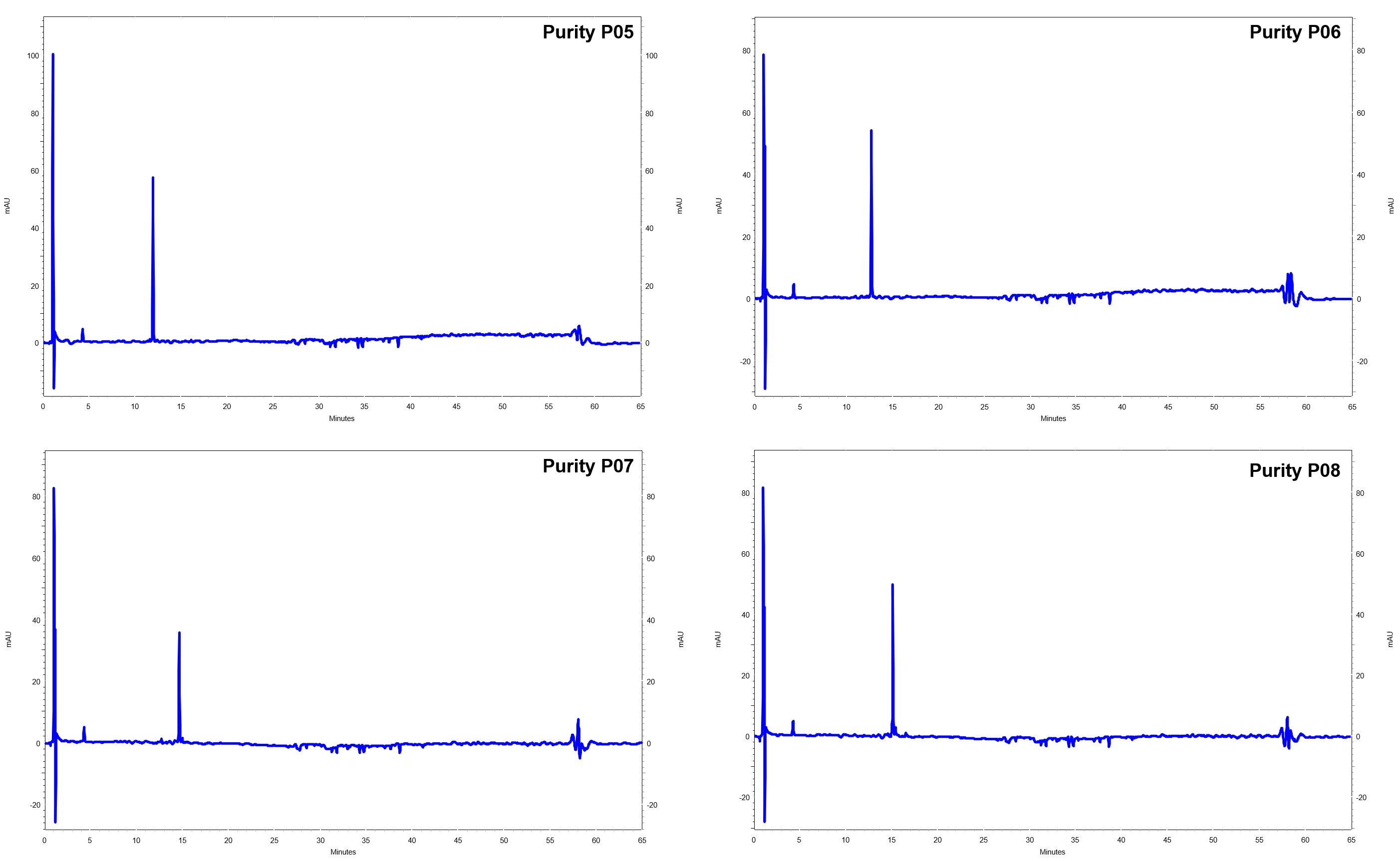


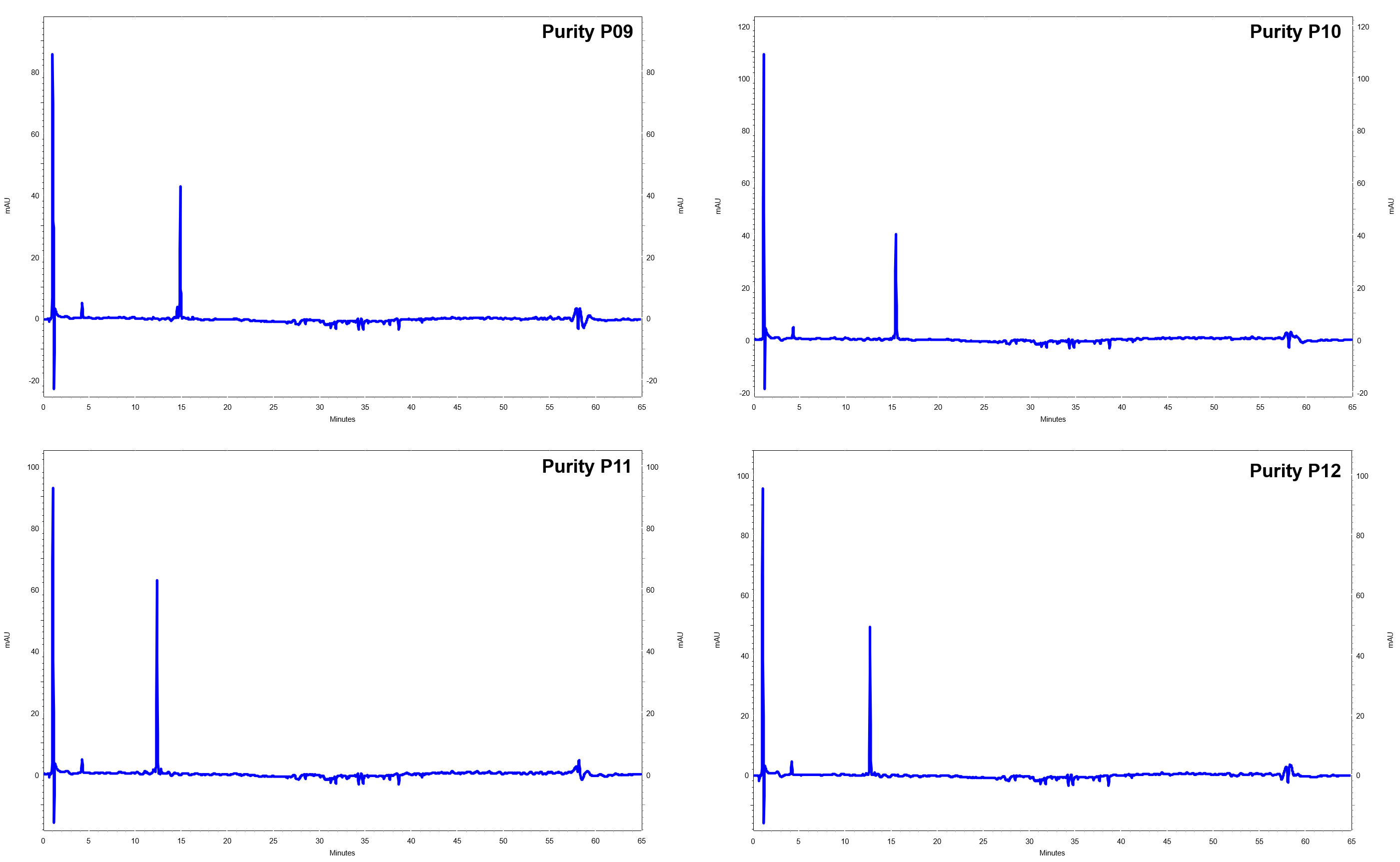


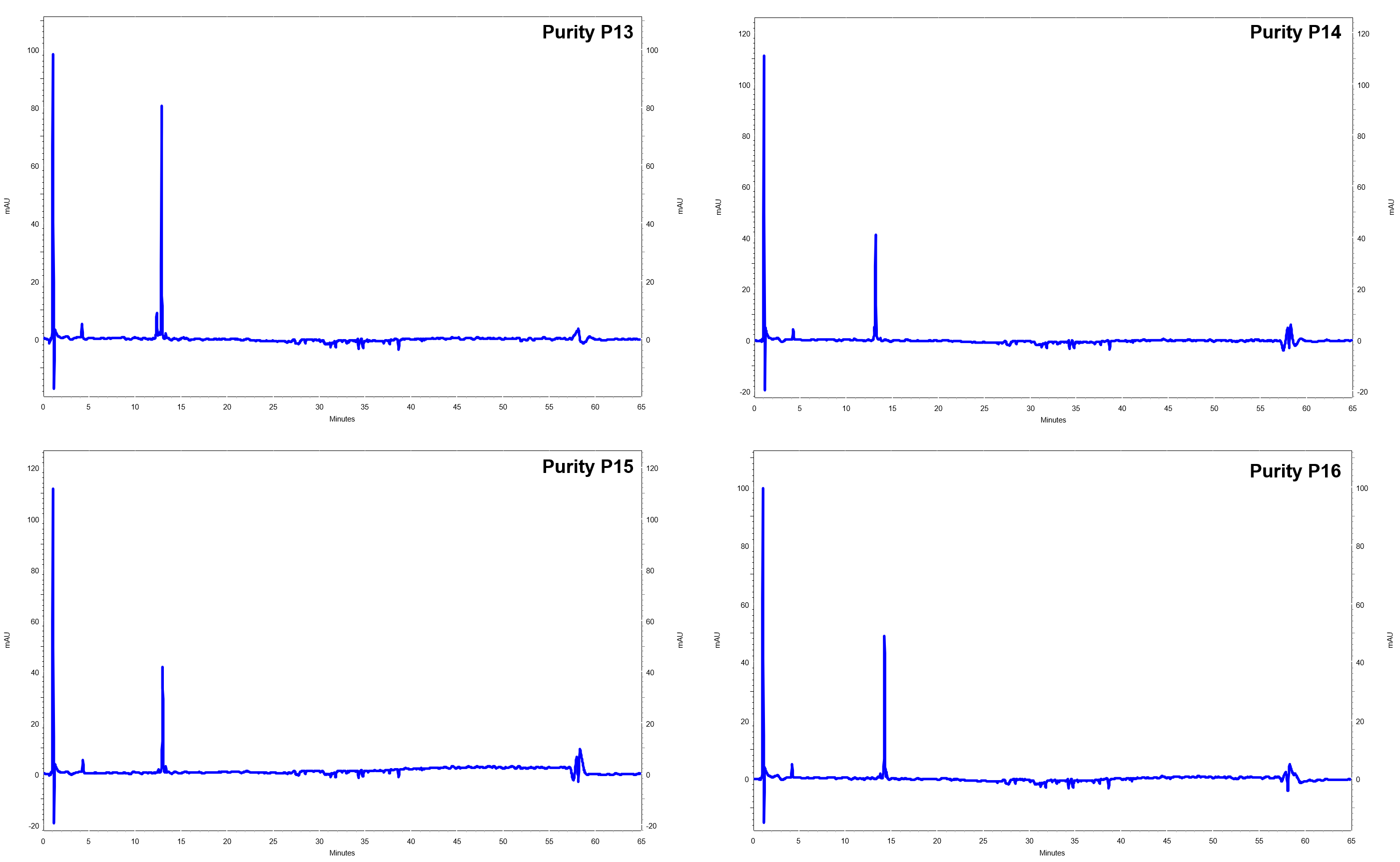


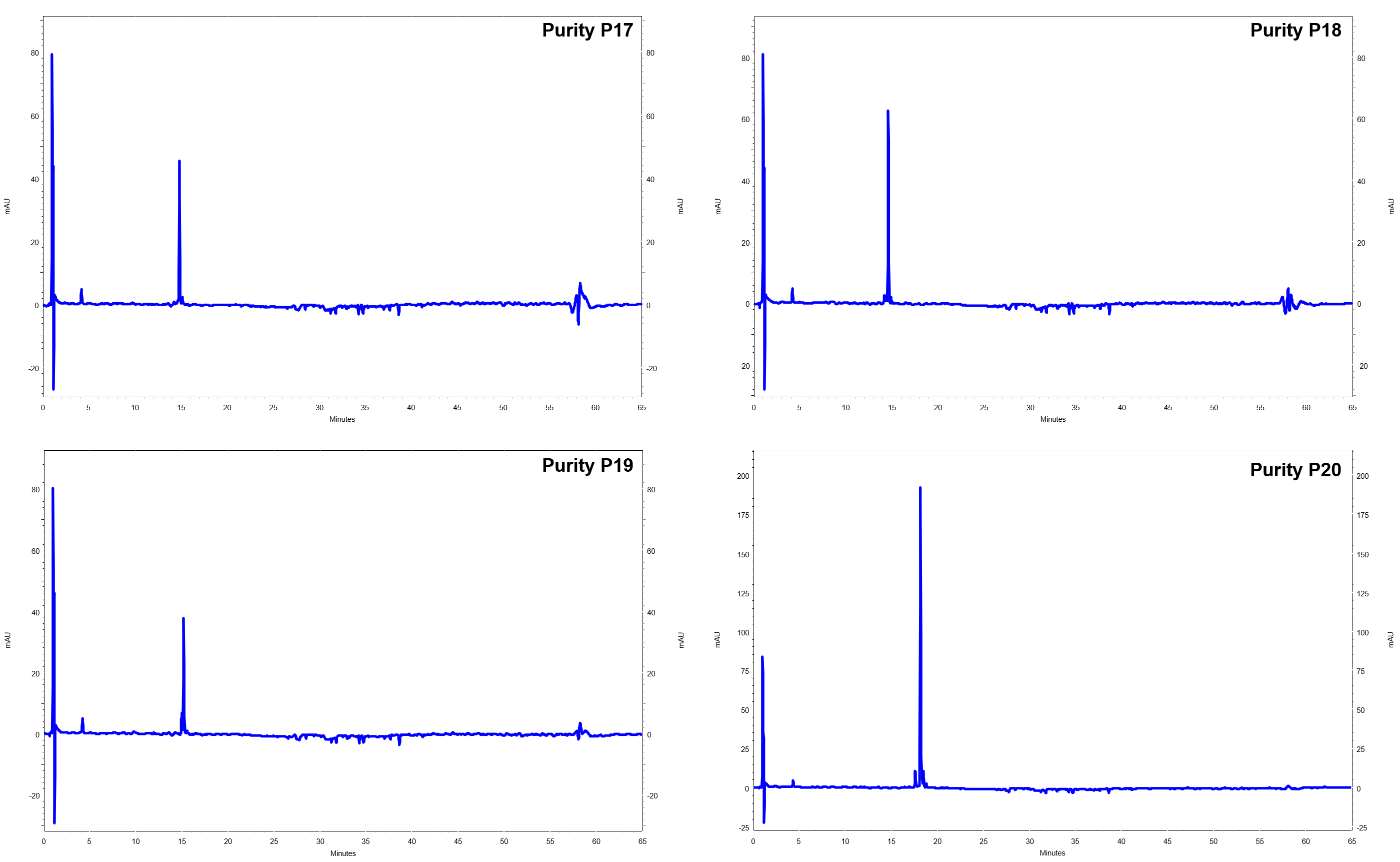


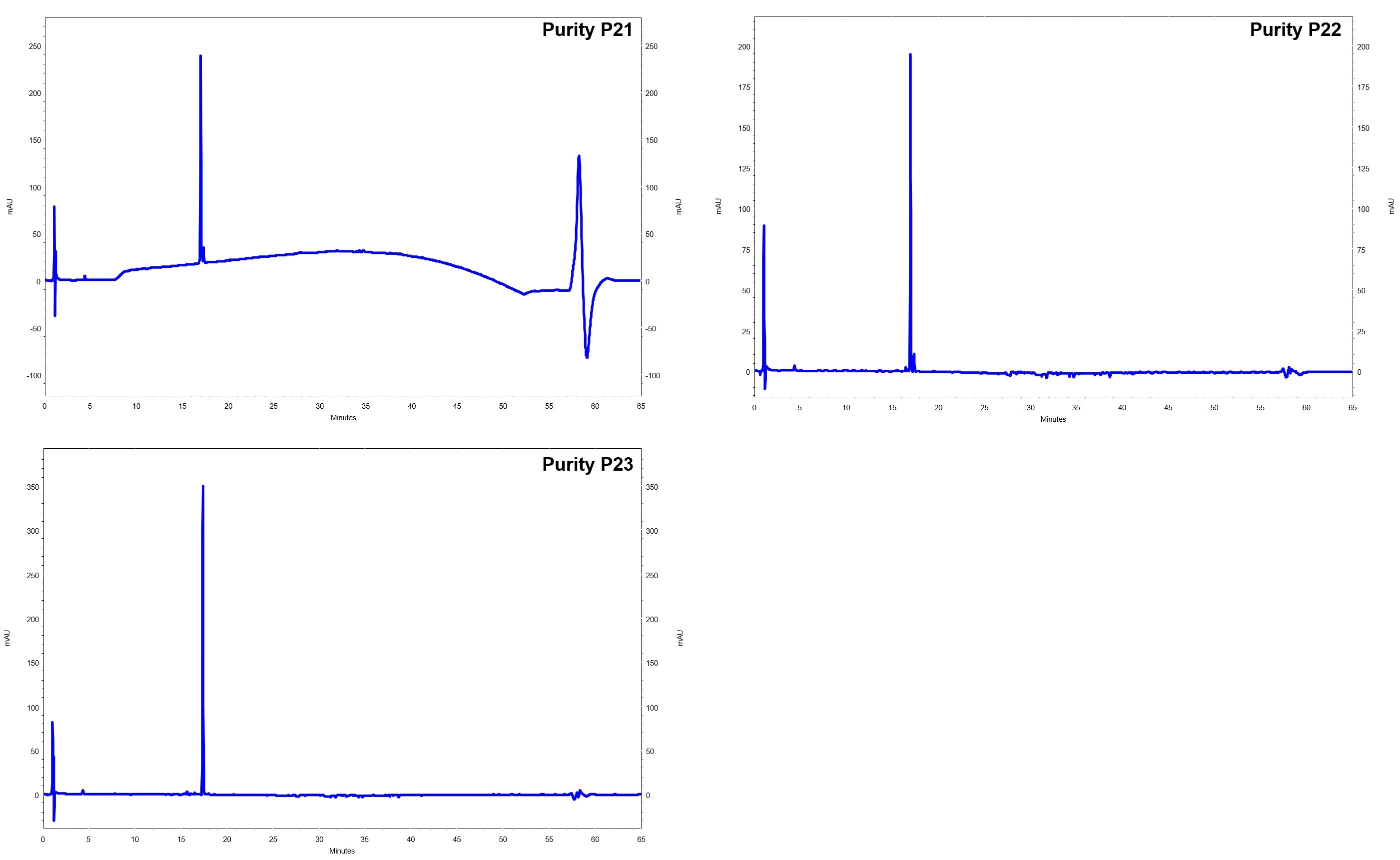


Figure SI-1.1: Peptide purity after synthesis and purification determined by HPLC-UV


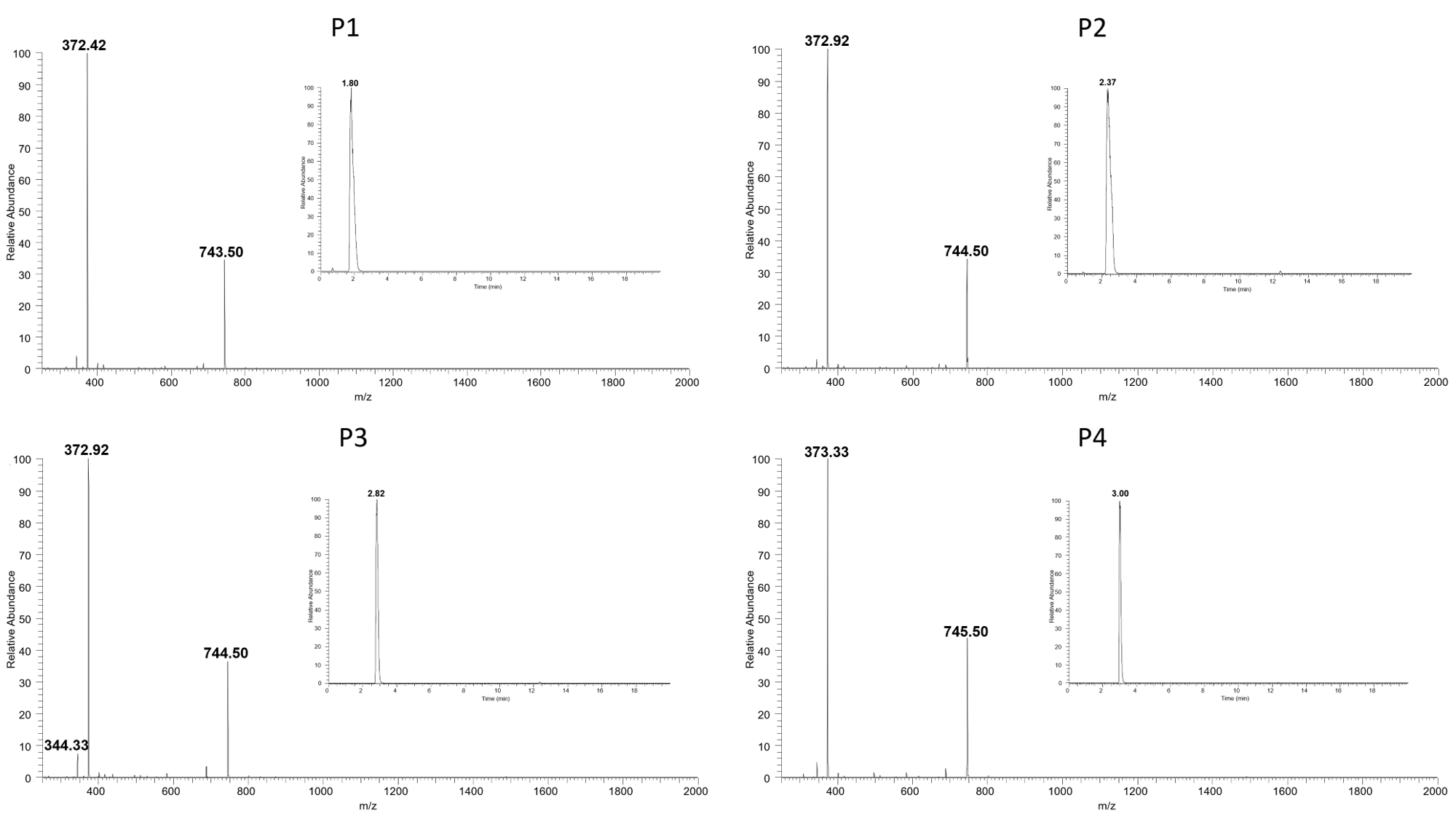


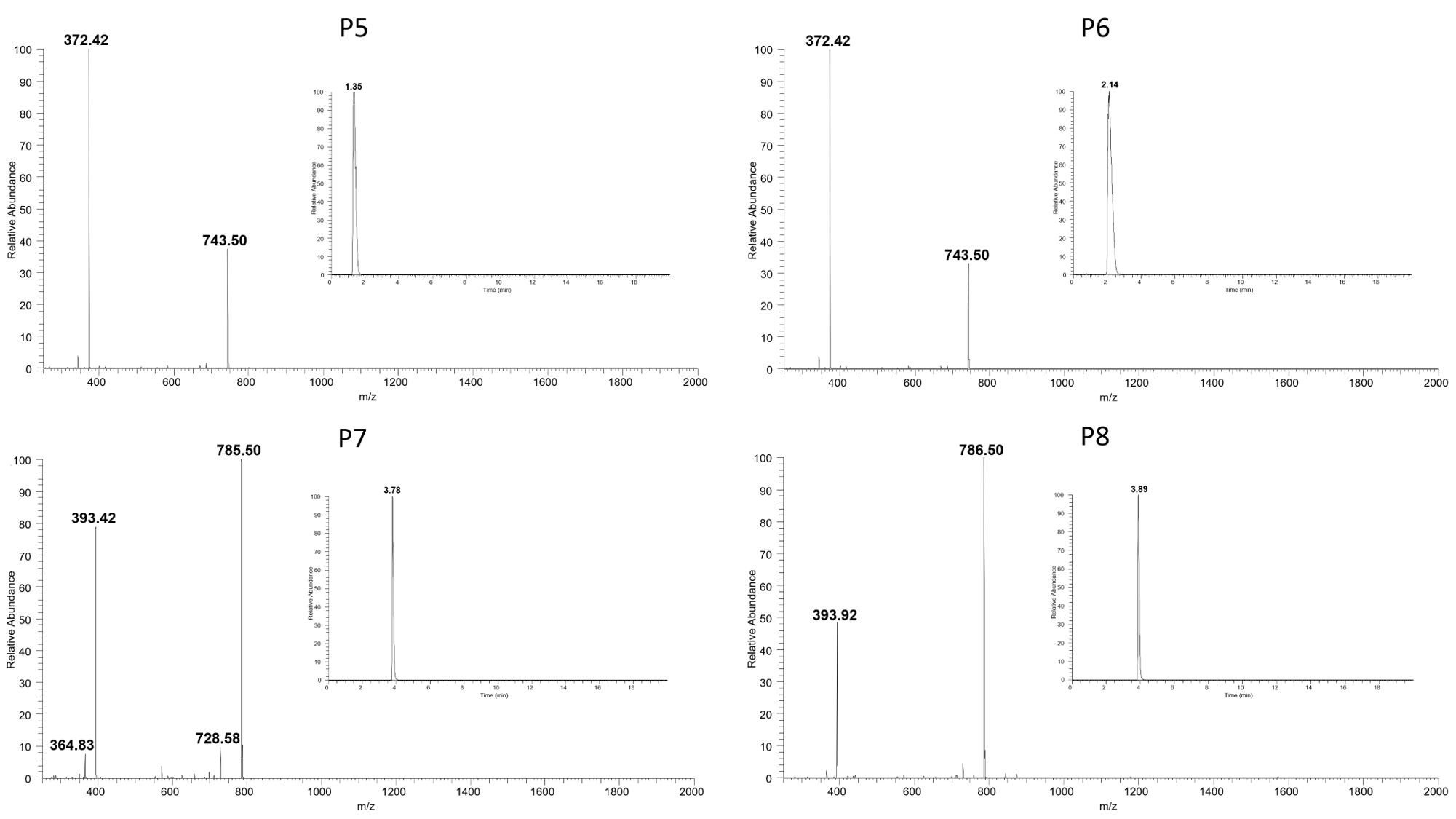


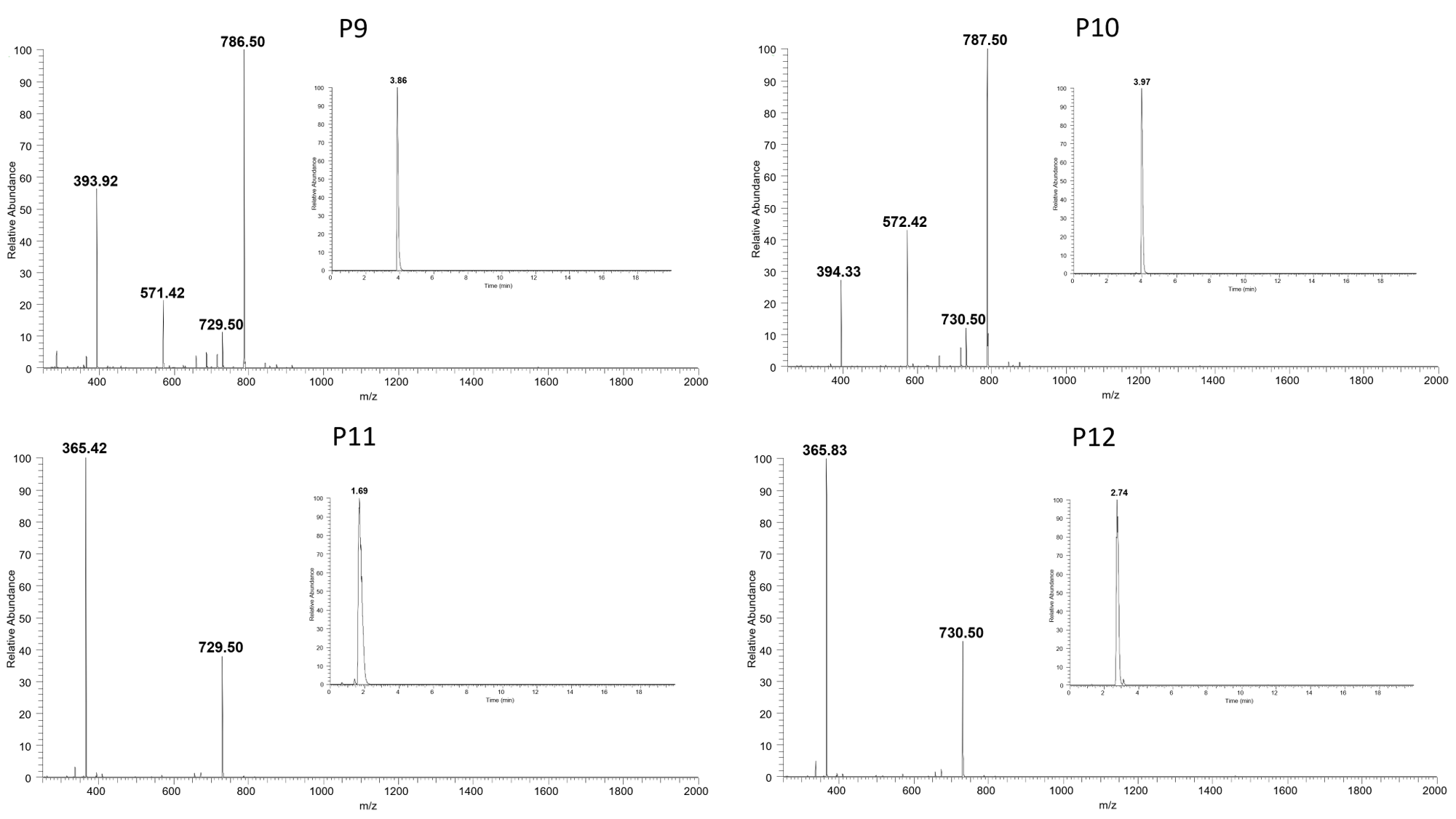


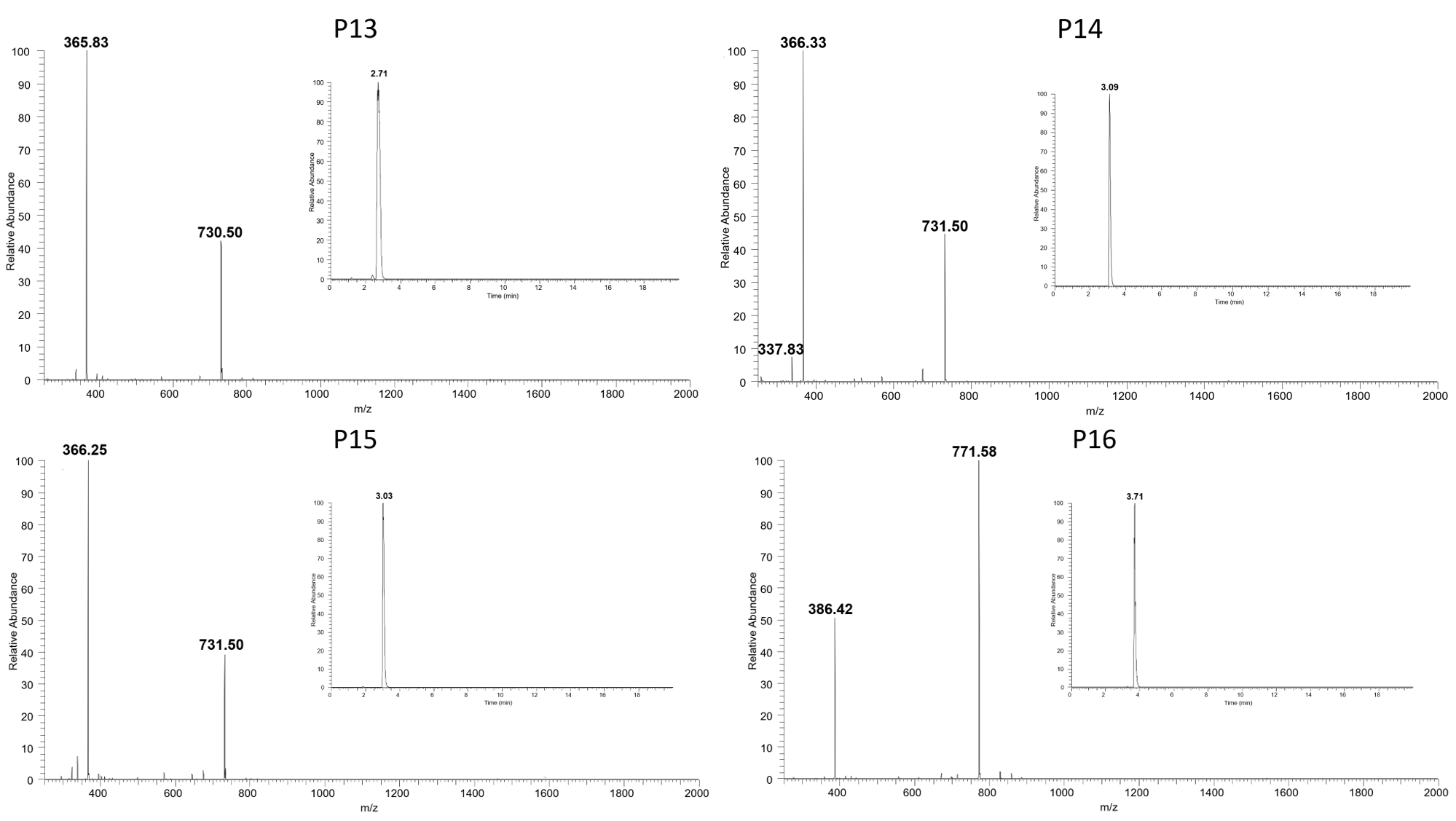


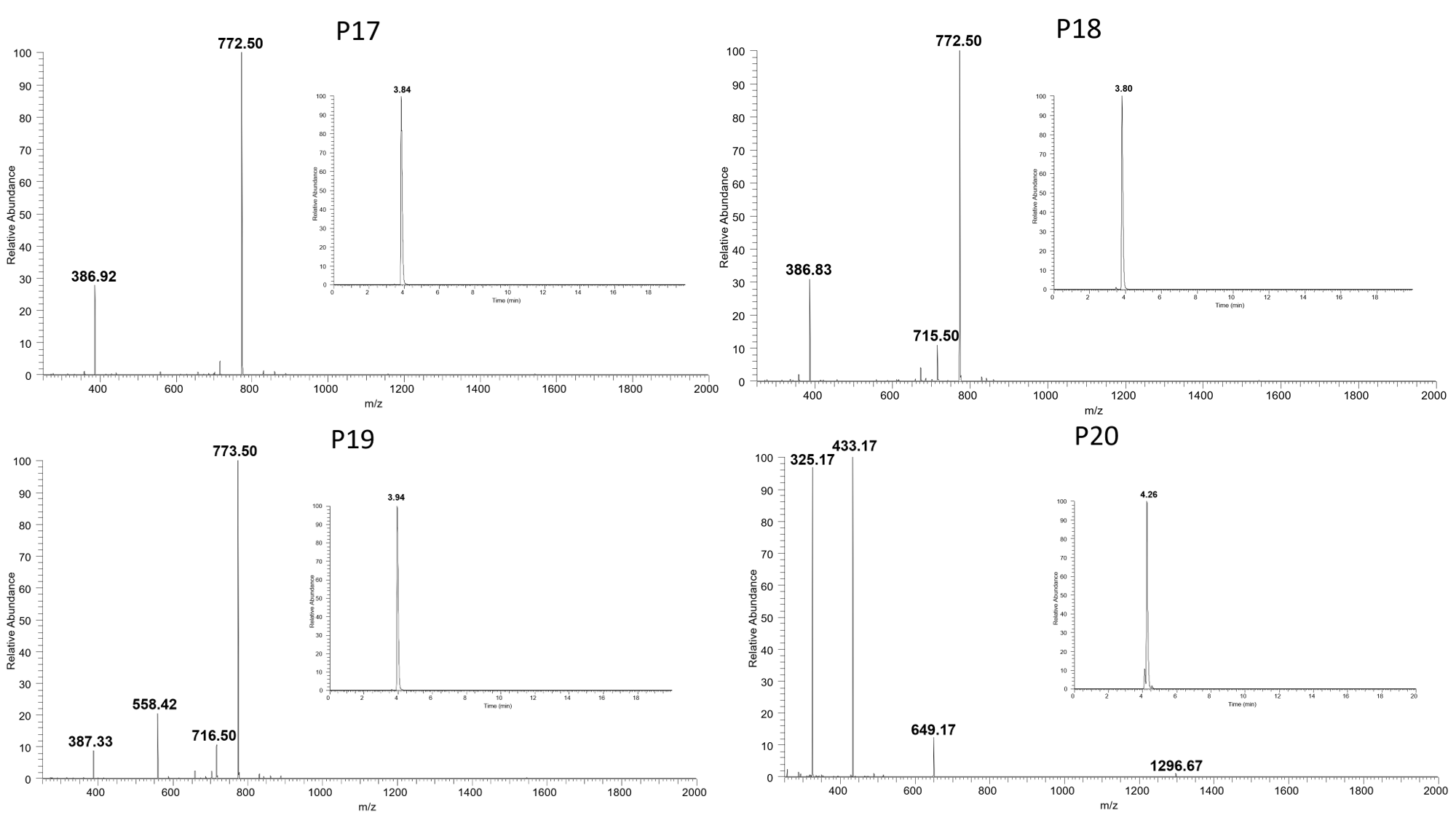


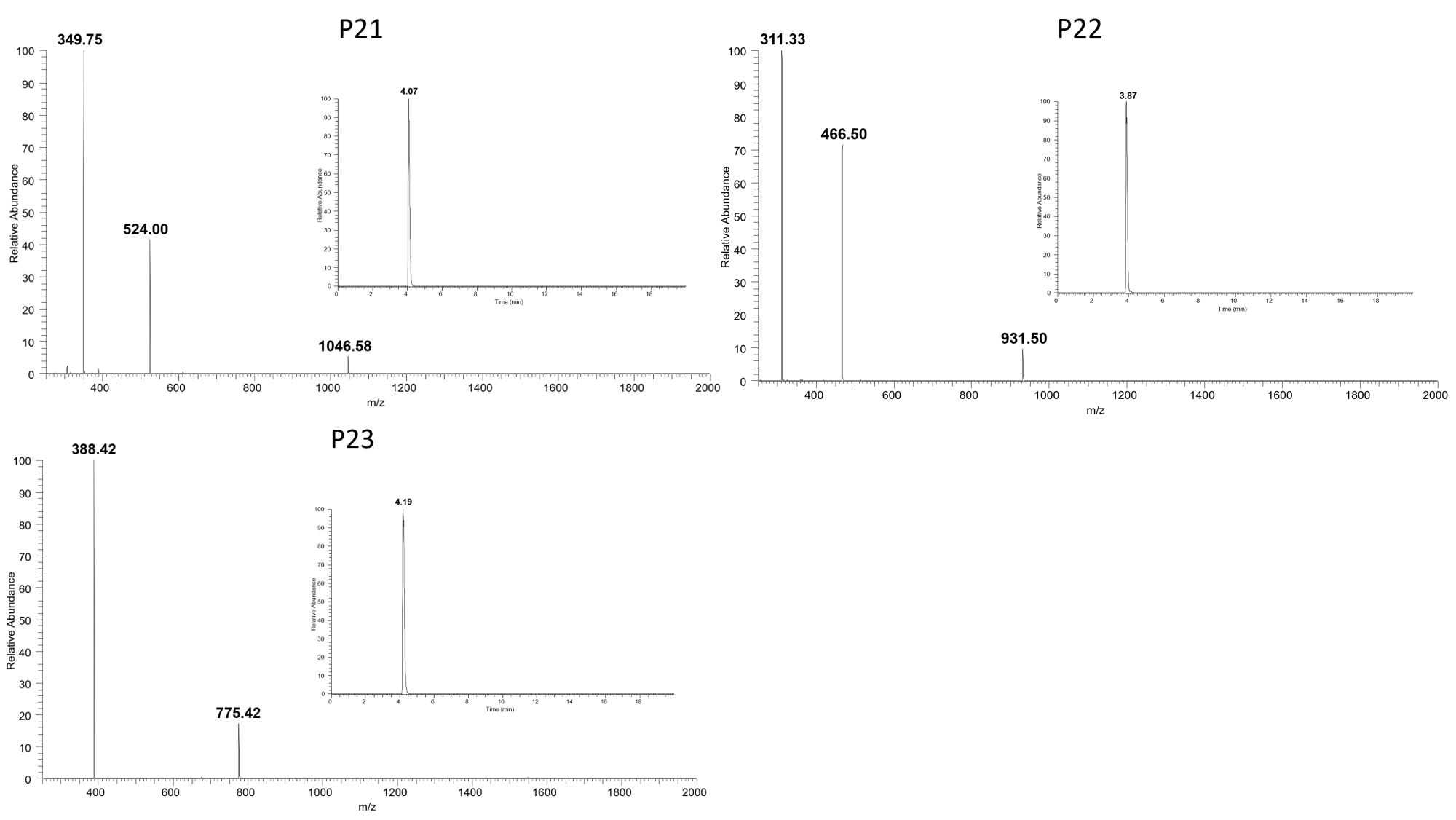


Figure SI-1.2: RP-LC chromatograms and mass spectra of P1-P23

# SI-2: Individual chromatograms to test the effect of the column choice on the separation efficacy


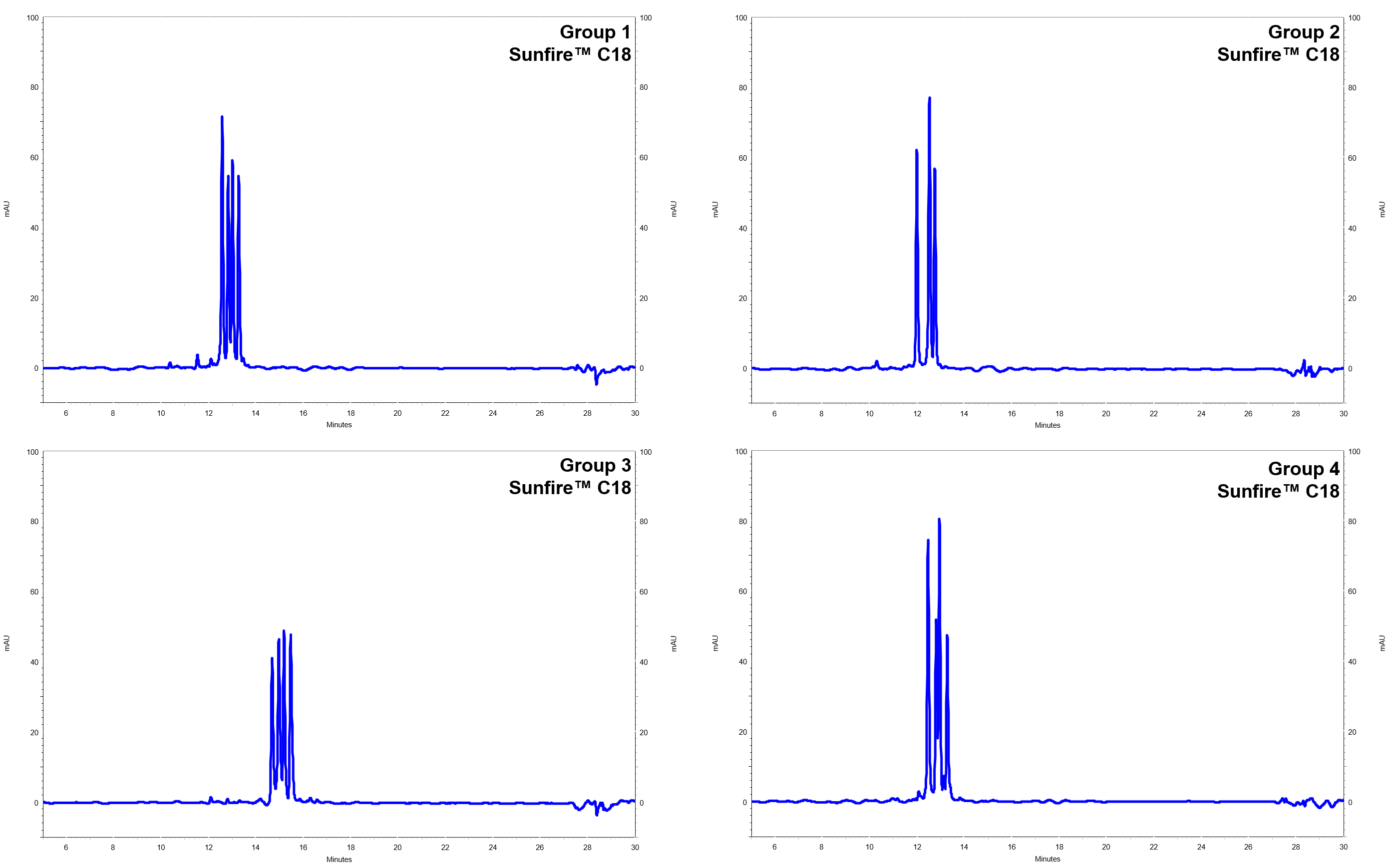


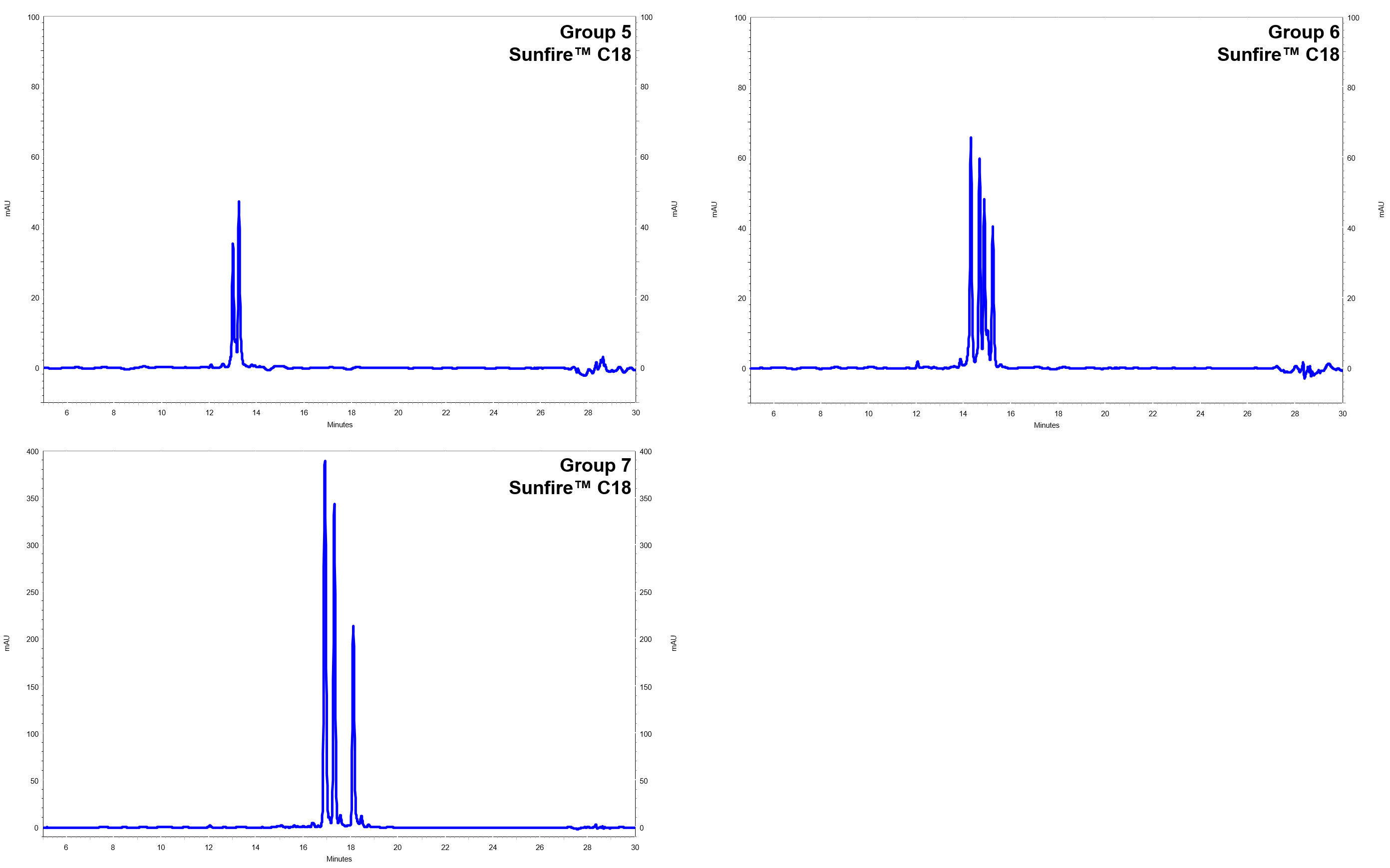


Figure SI-2.1: RP-LC chromatograms and mass spectra of impurity groups 1-7 measured with the Sunfire™ C18.


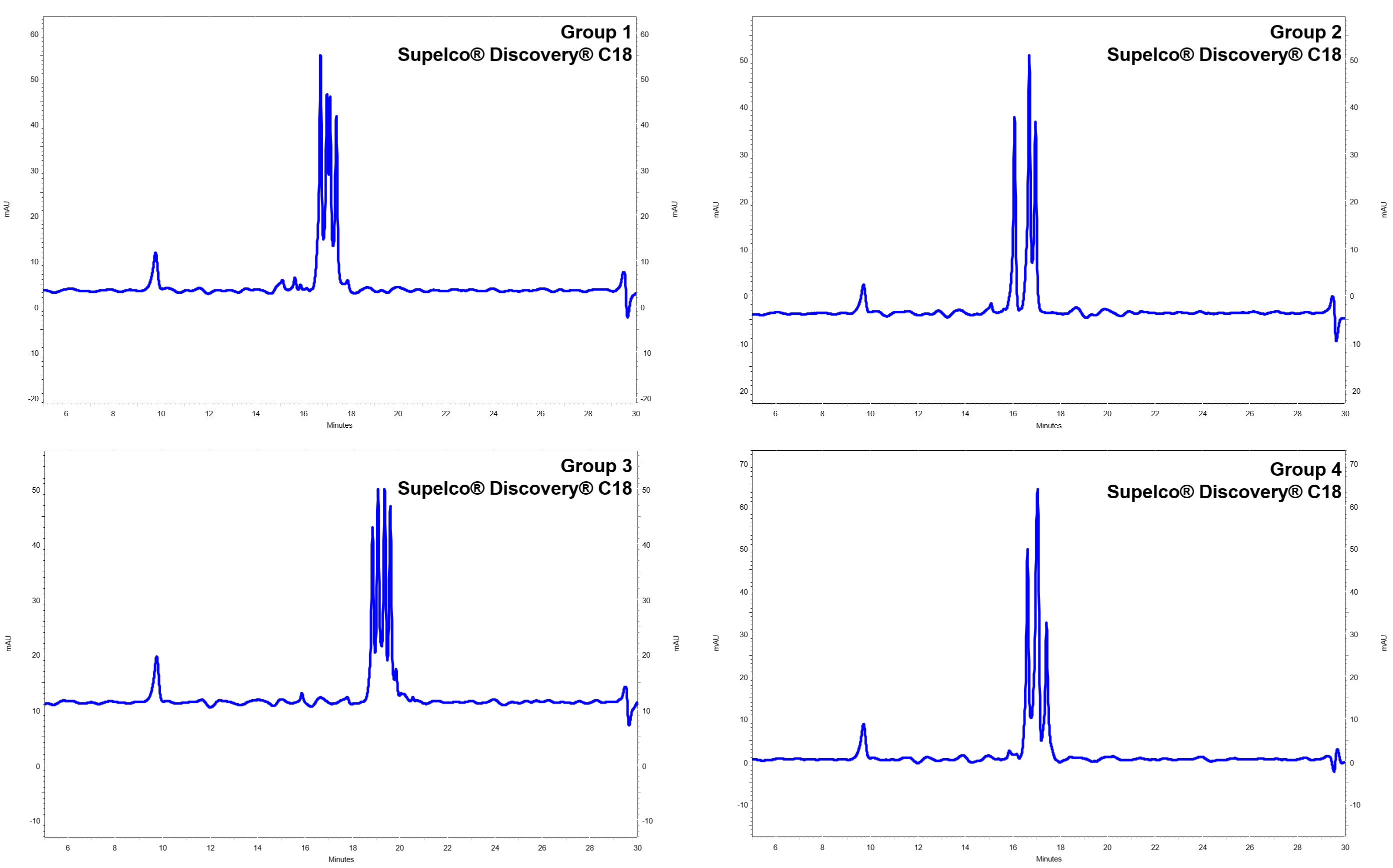


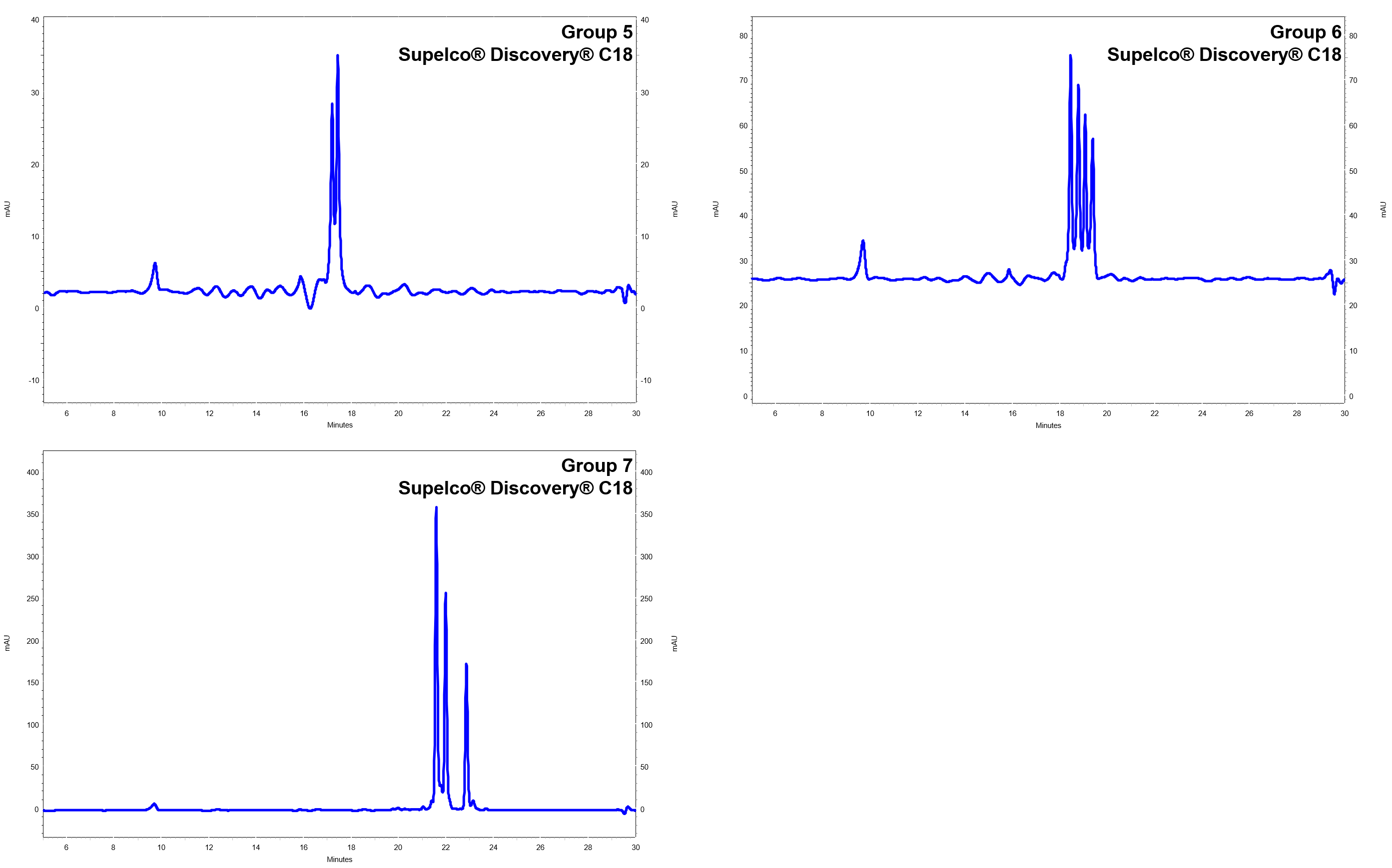


Figure SI-2.2: RP-LC chromatograms and mass spectra of impurity groups 1-7 measured with the Supelco® Discovery® C18.


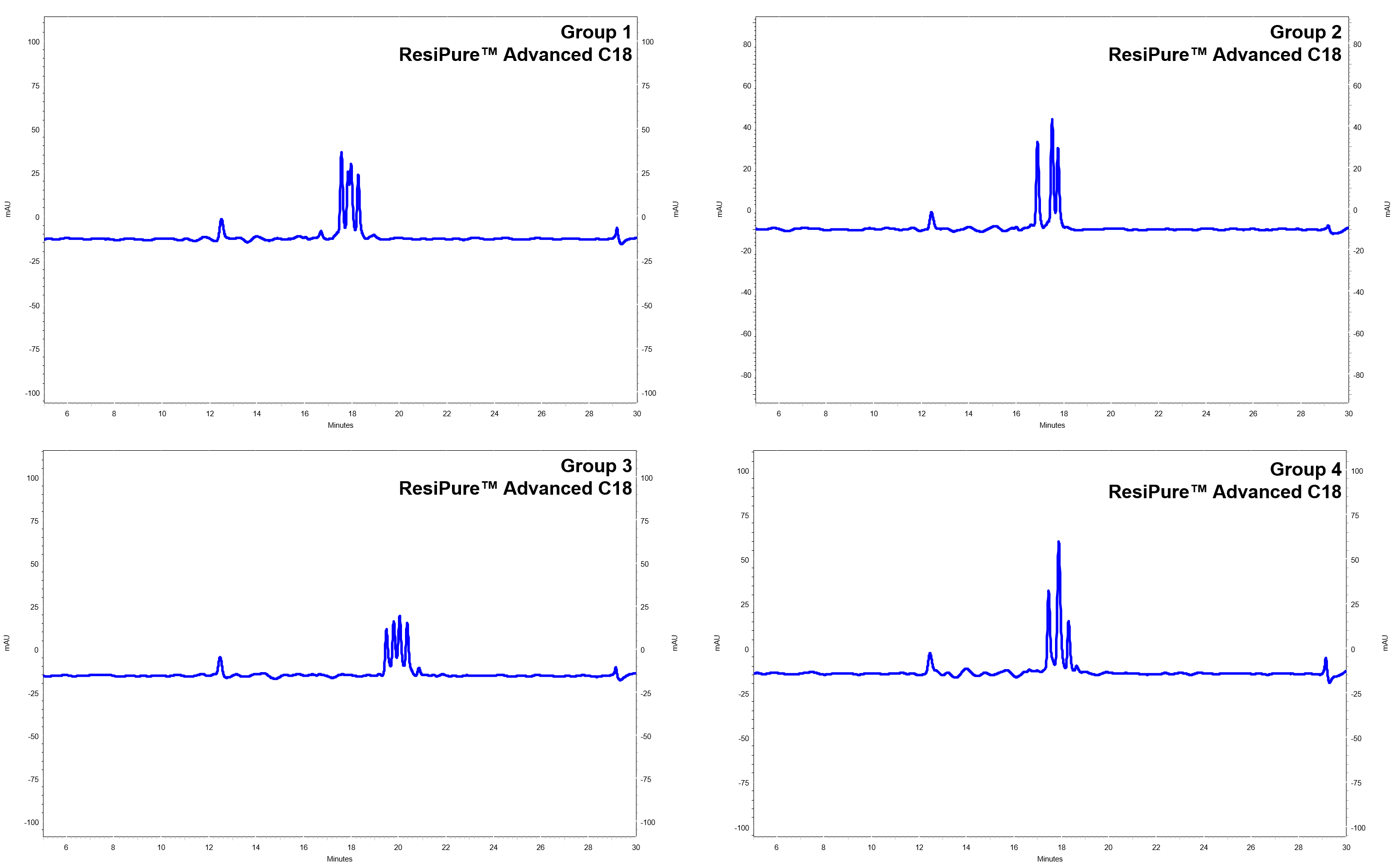


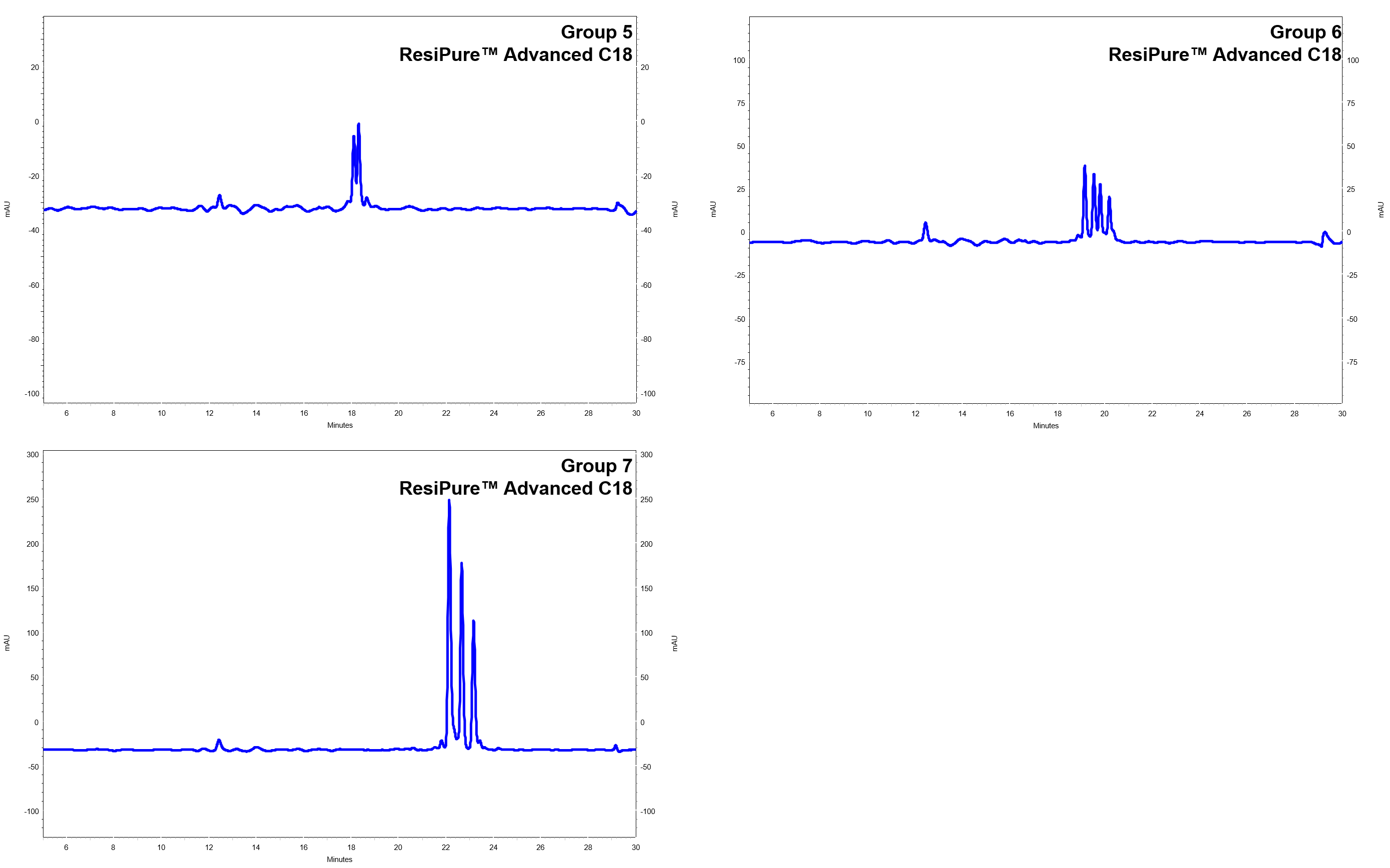


Figure SI-2.3: RP-LC chromatograms and mass spectra of impurity groups 1-7 measured with the ResiPure™ Advanced C18.


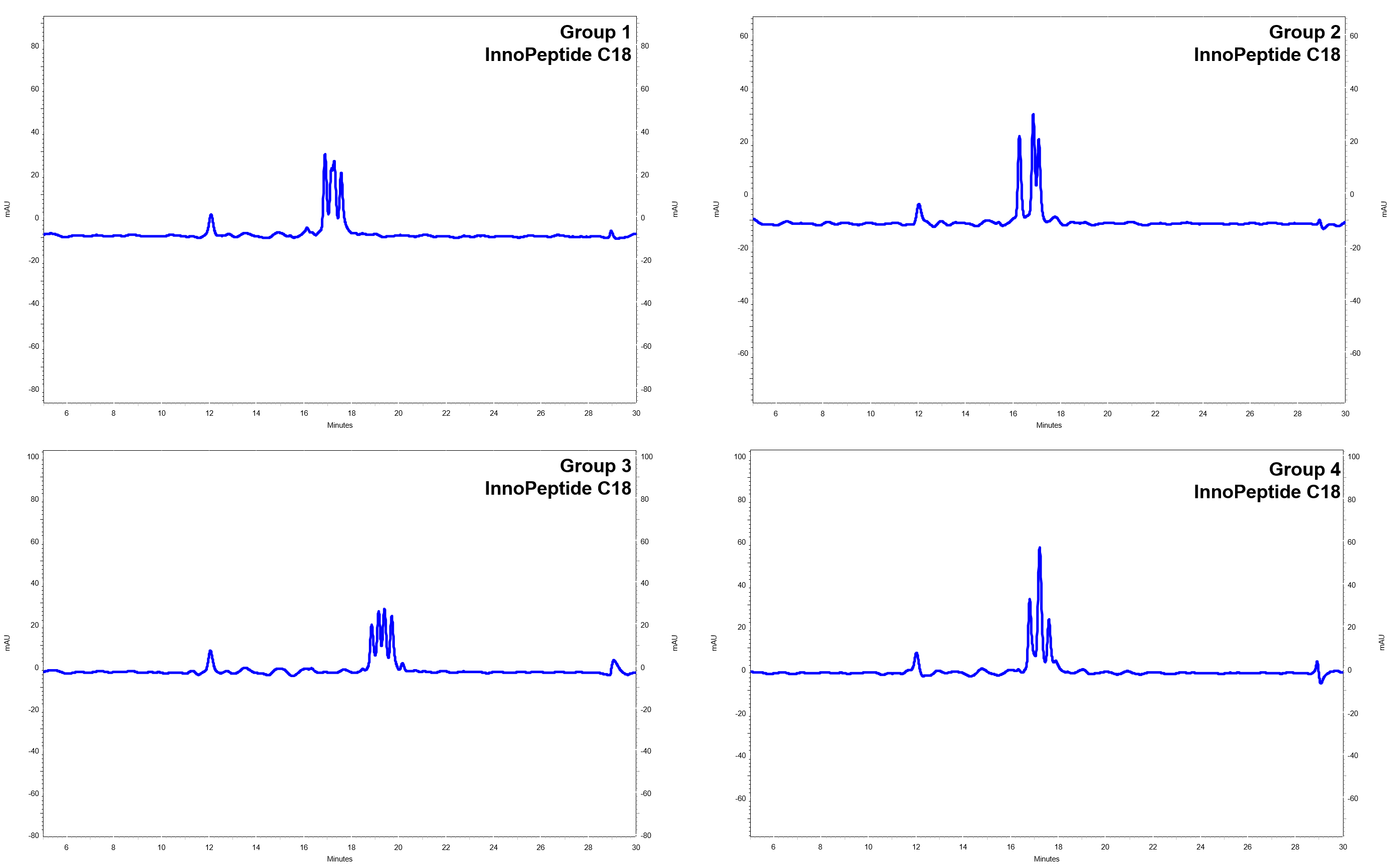


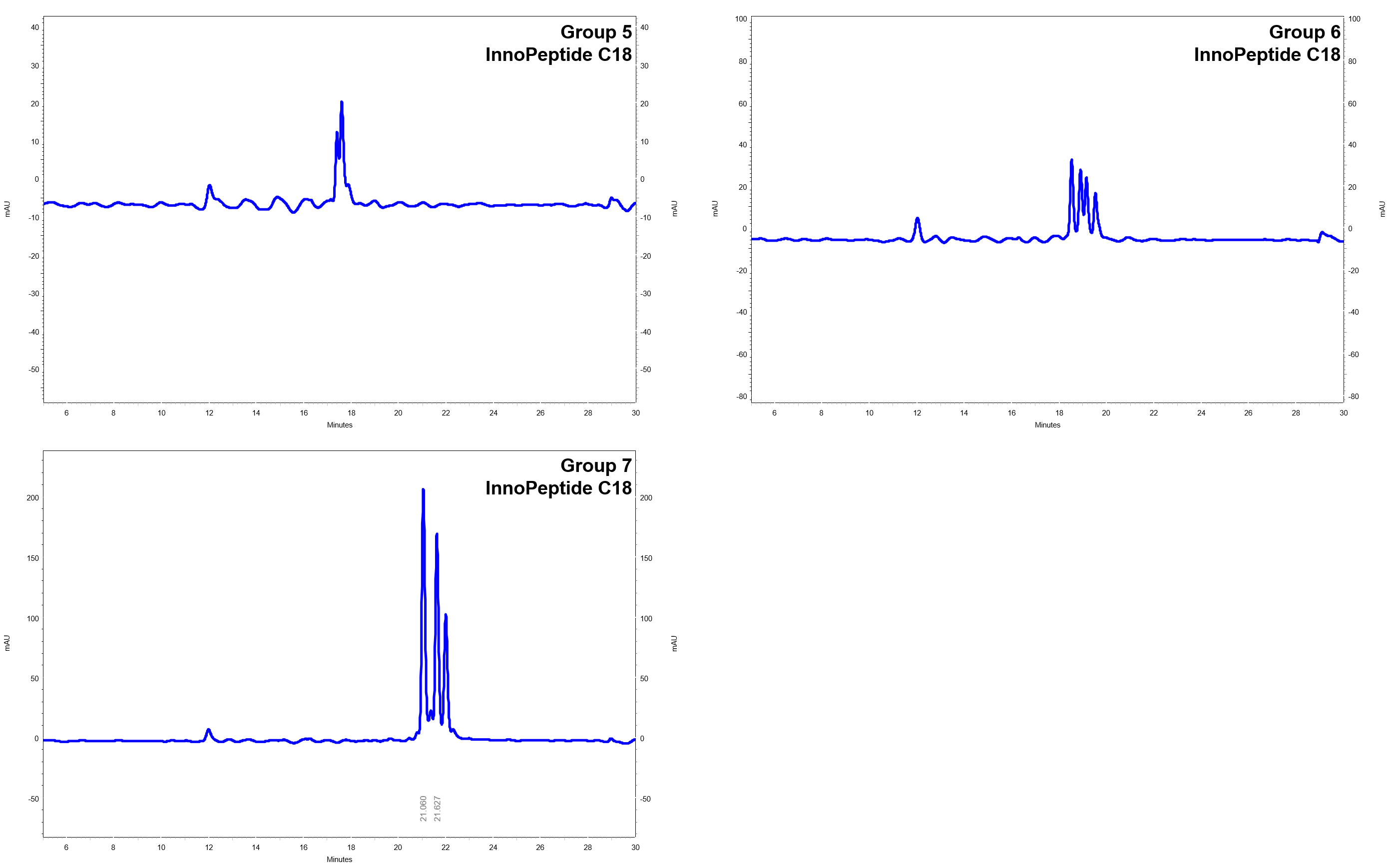


Figure SI-2.4: RP-LC chromatograms and mass spectra of impurity groups 1-7 measured with the InnoPeptide C18.


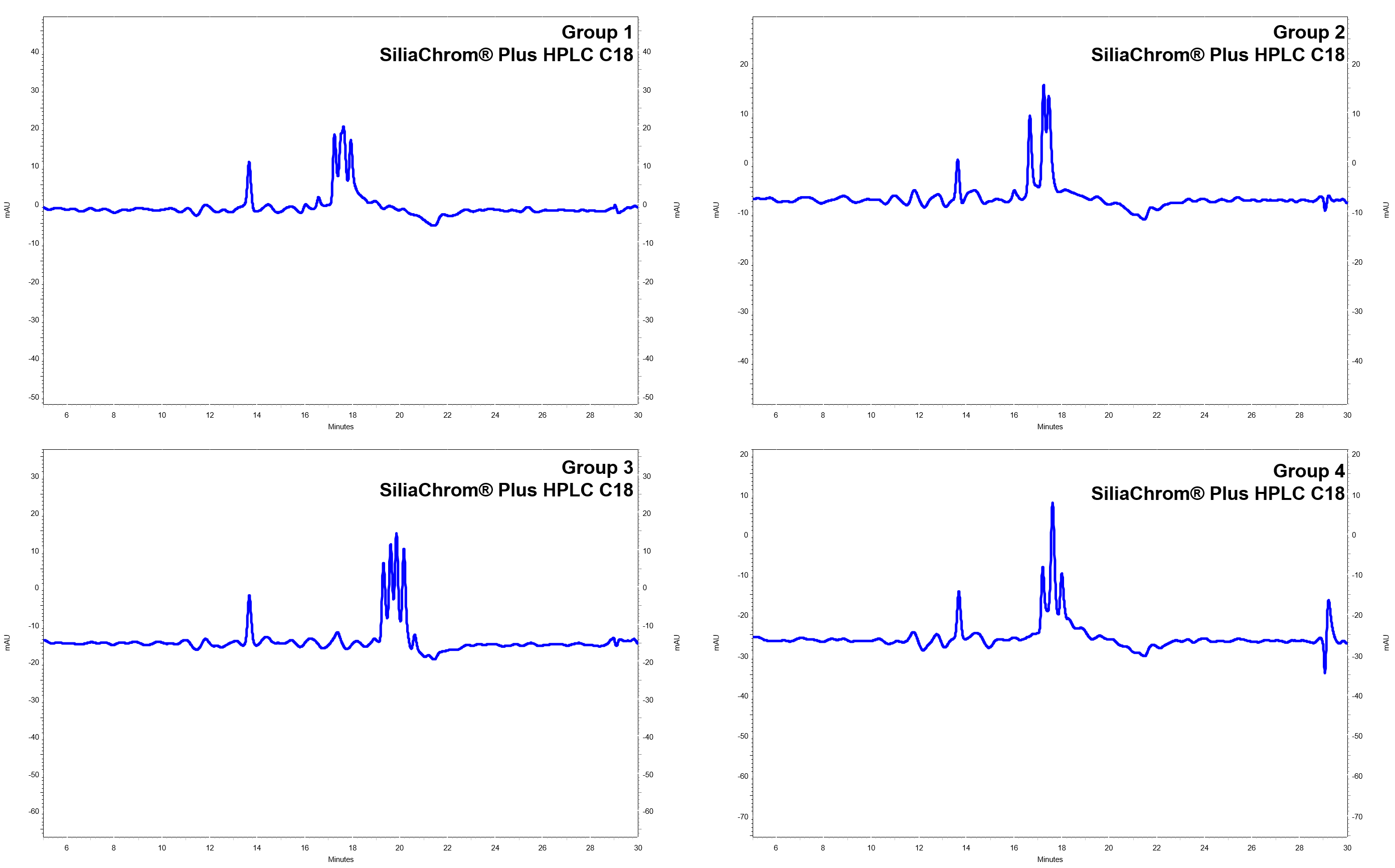


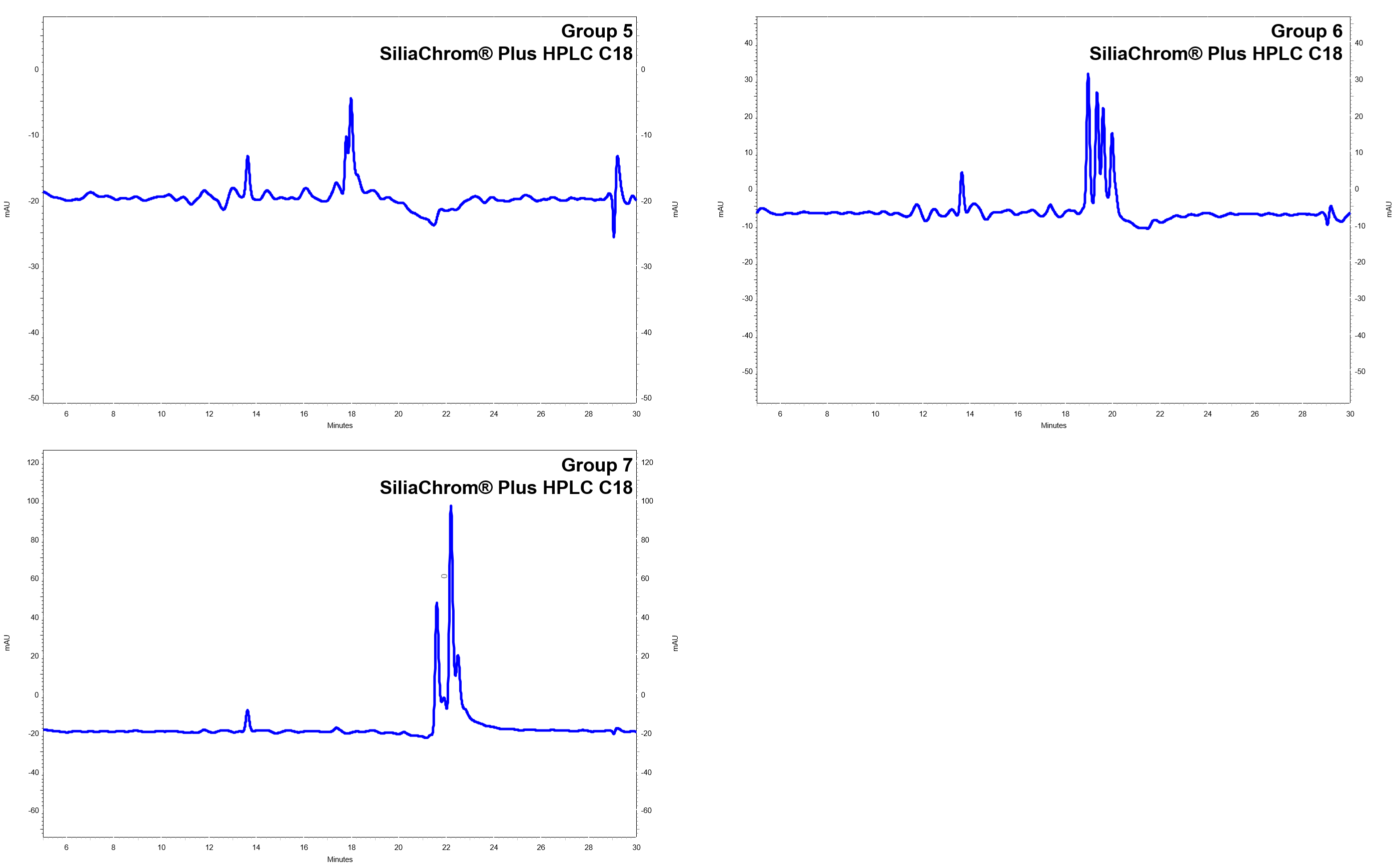


Figure SI-2.5: RP-LC chromatograms and mass spectra of impurity groups 1-7 measured with the SiliaChrom® Plus HPLC C18

# SI-3: Individual chromatograms to test the effect of the gradient steepness on the separation efficacy


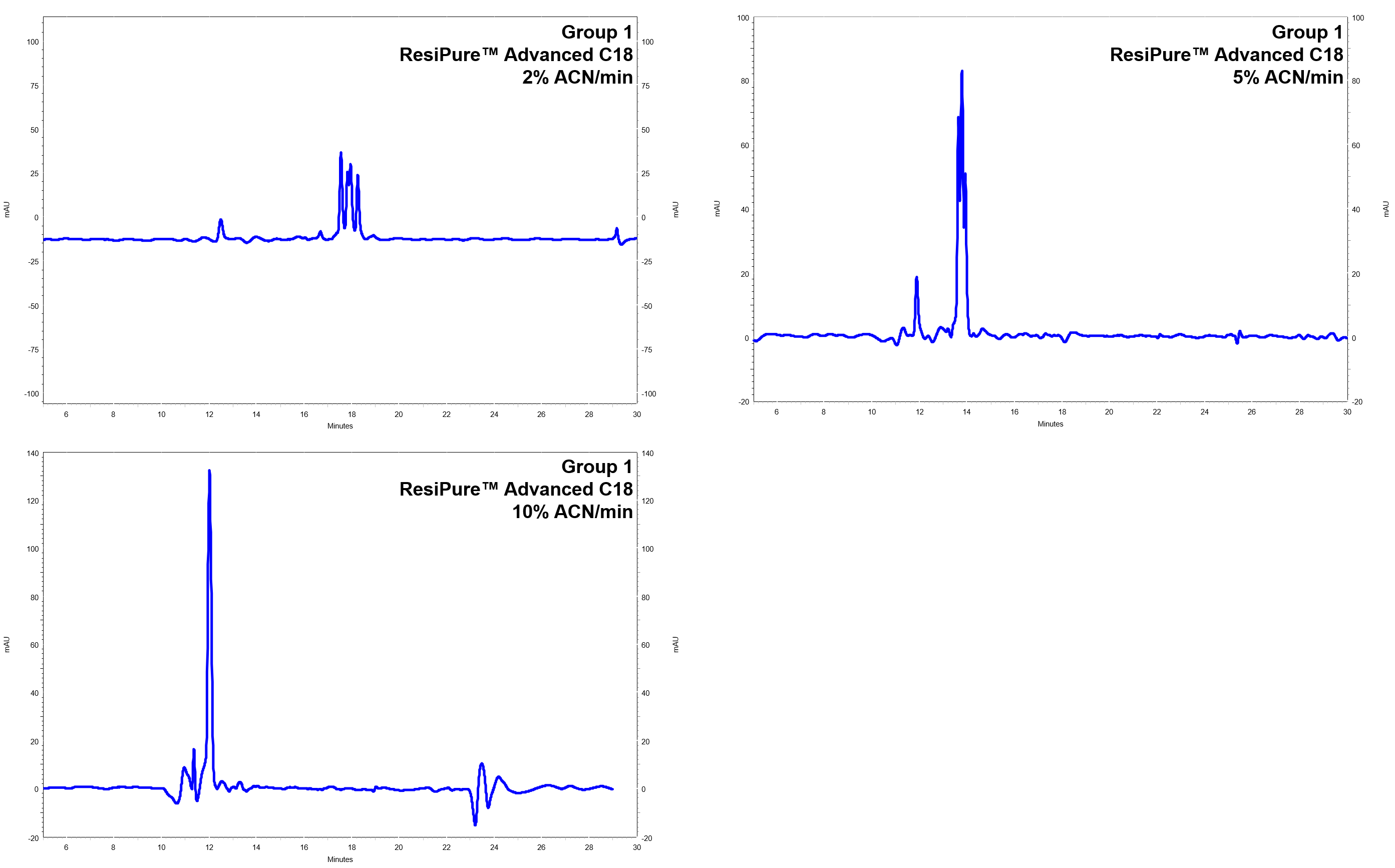


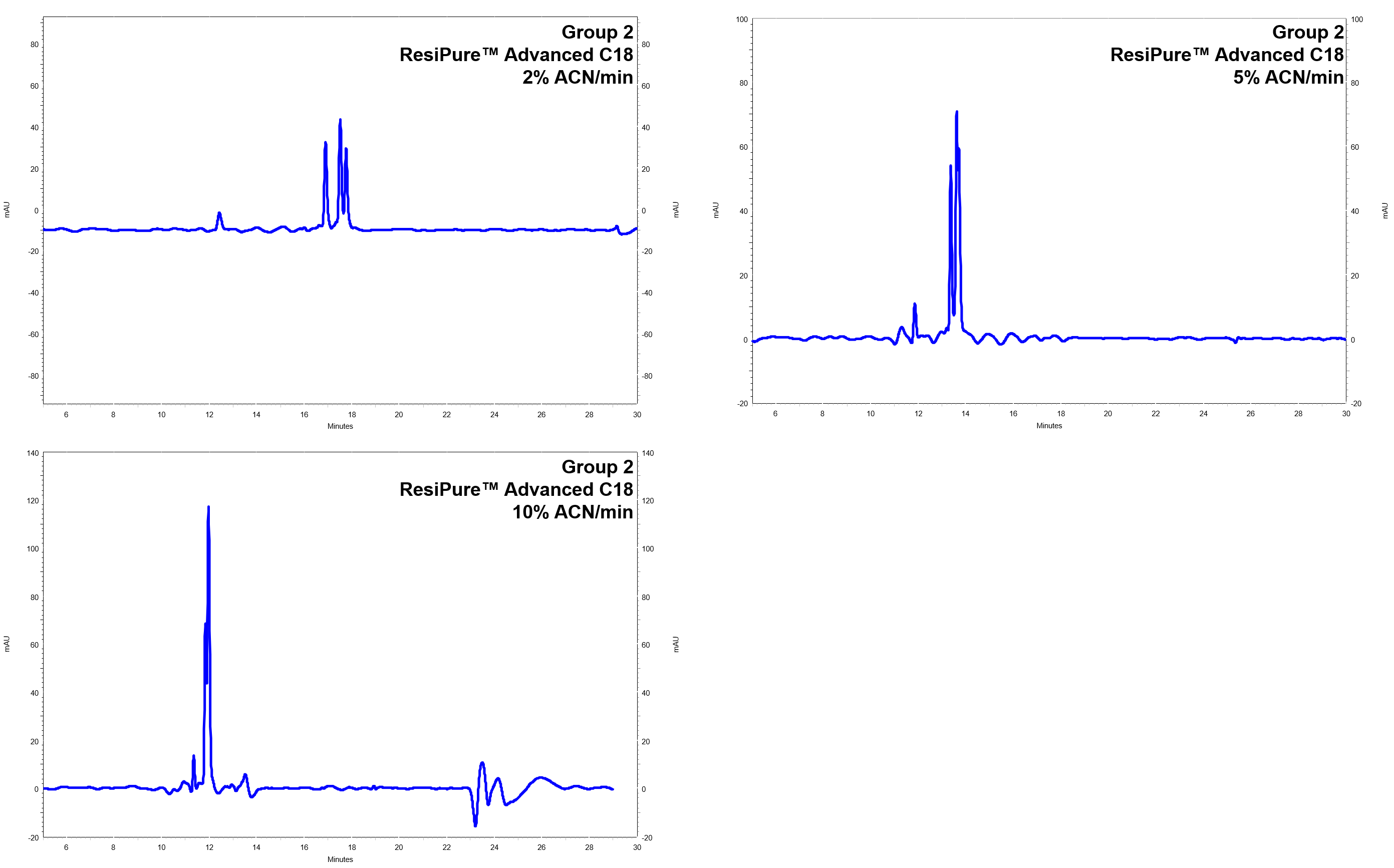


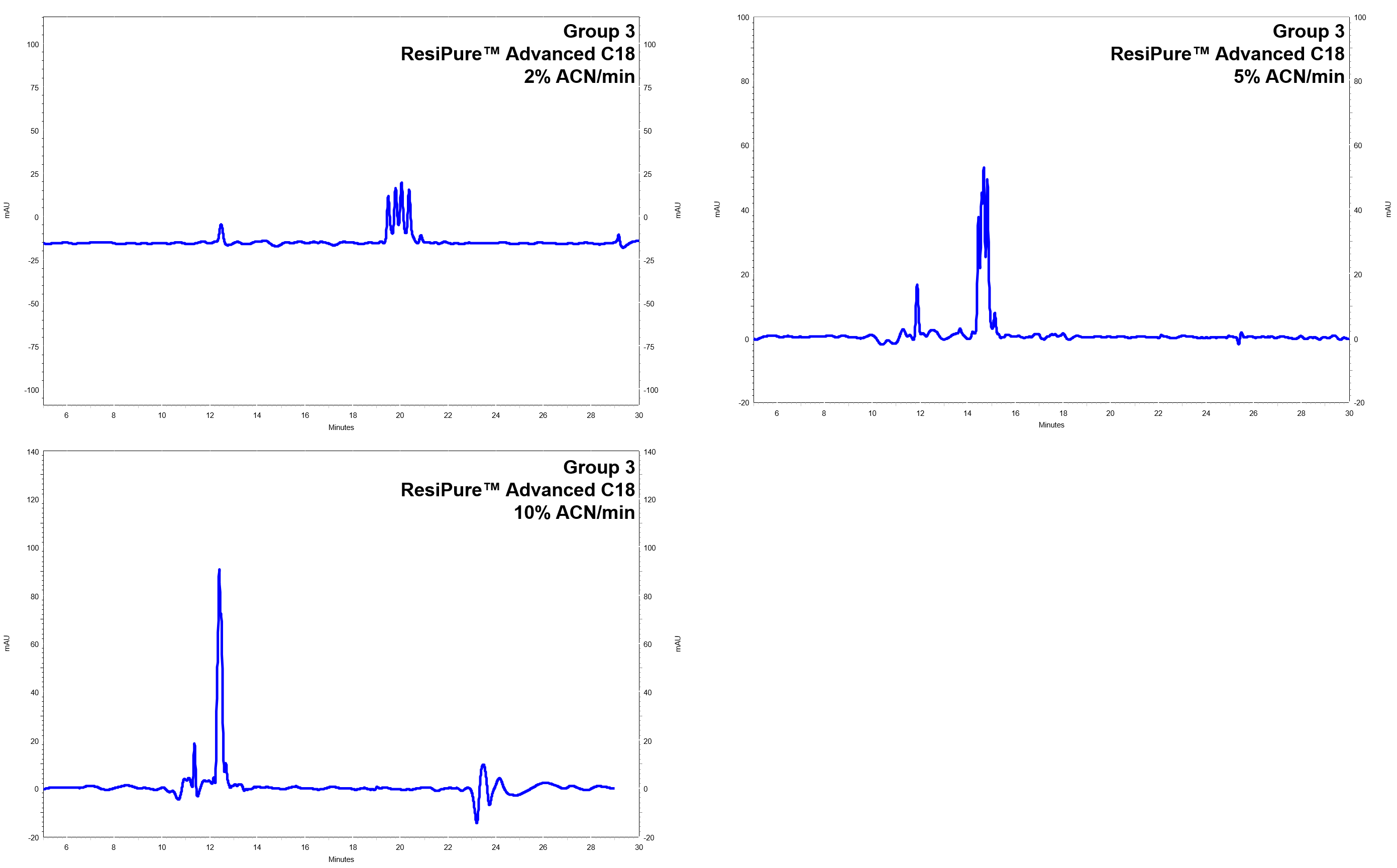


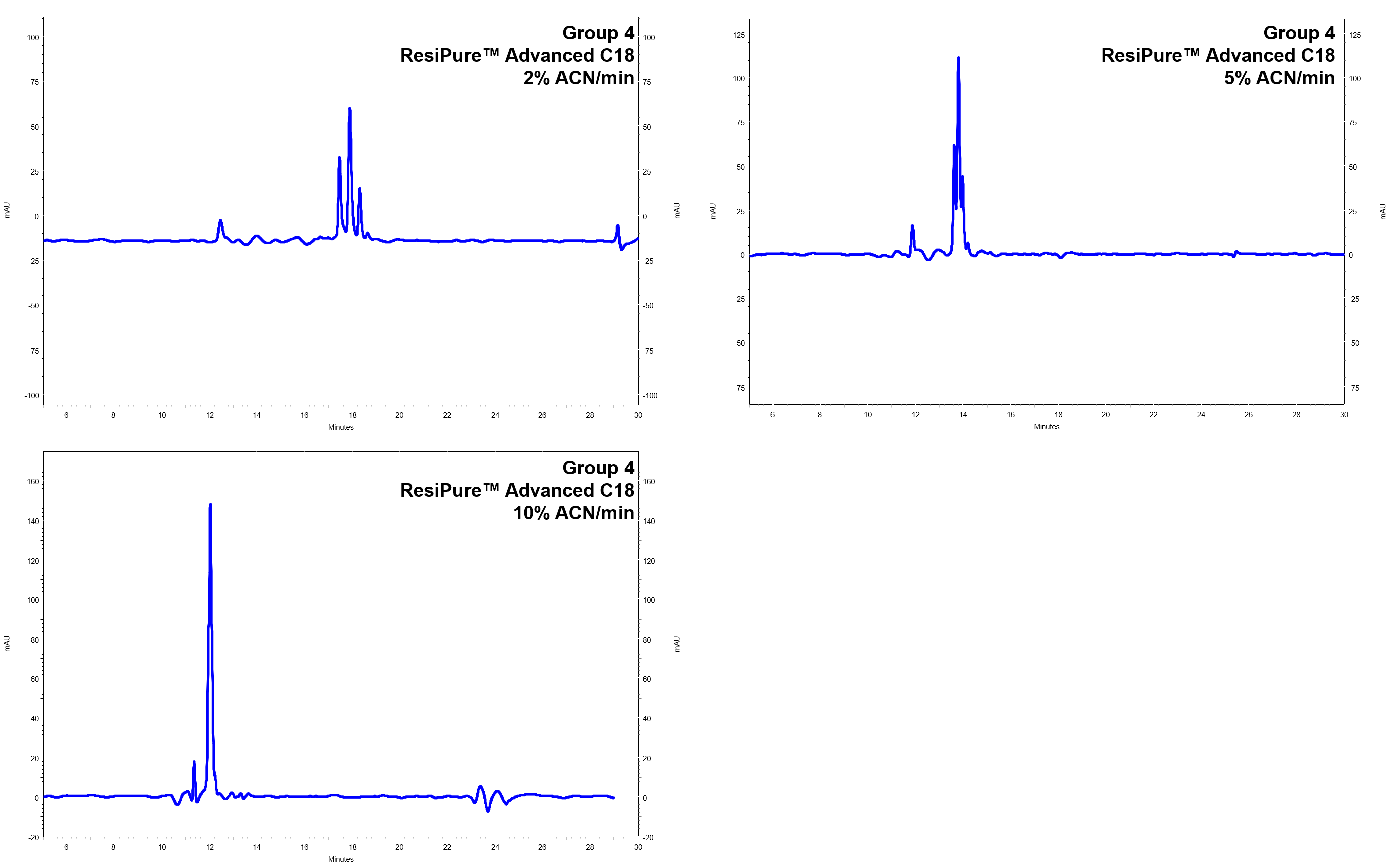


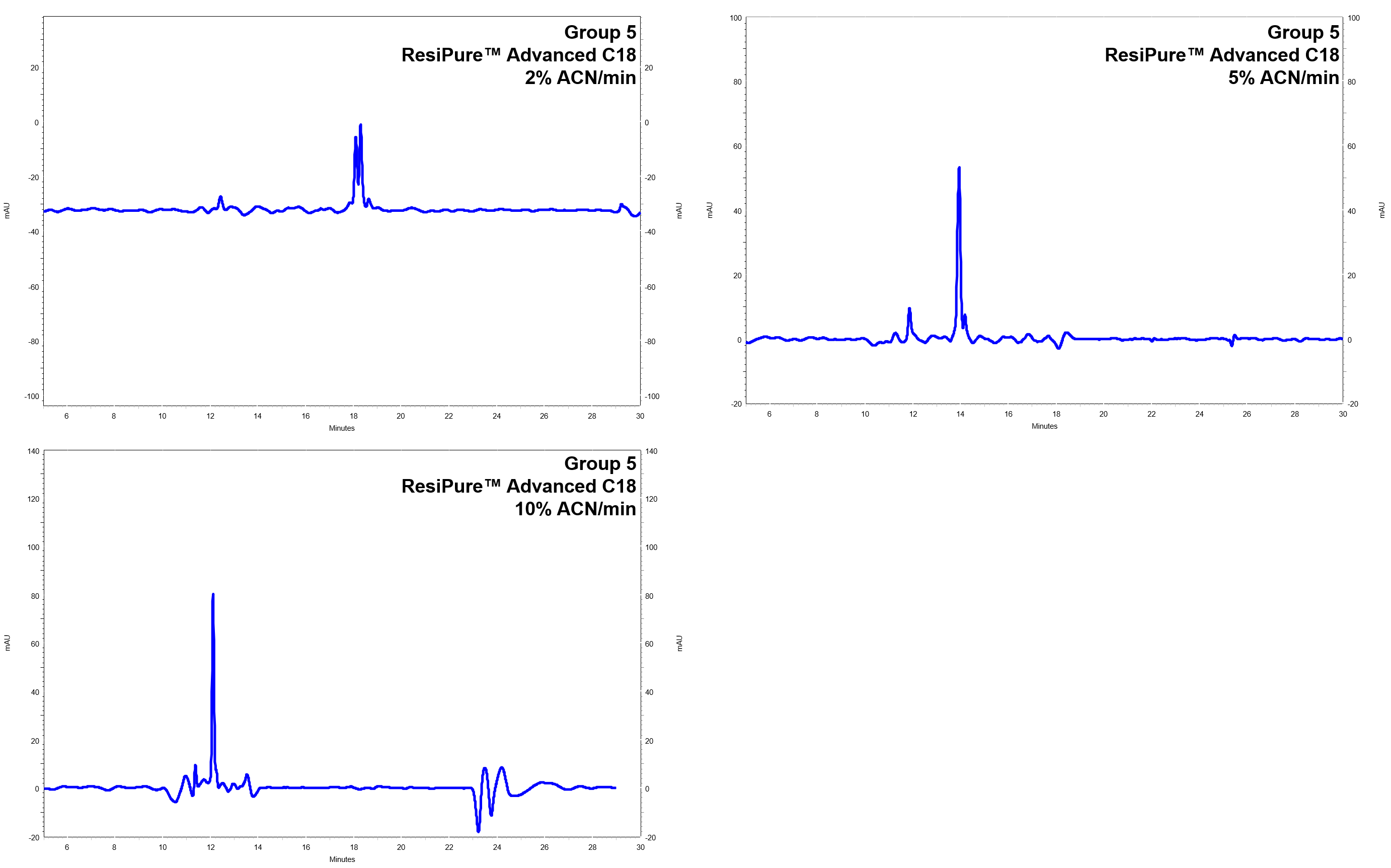


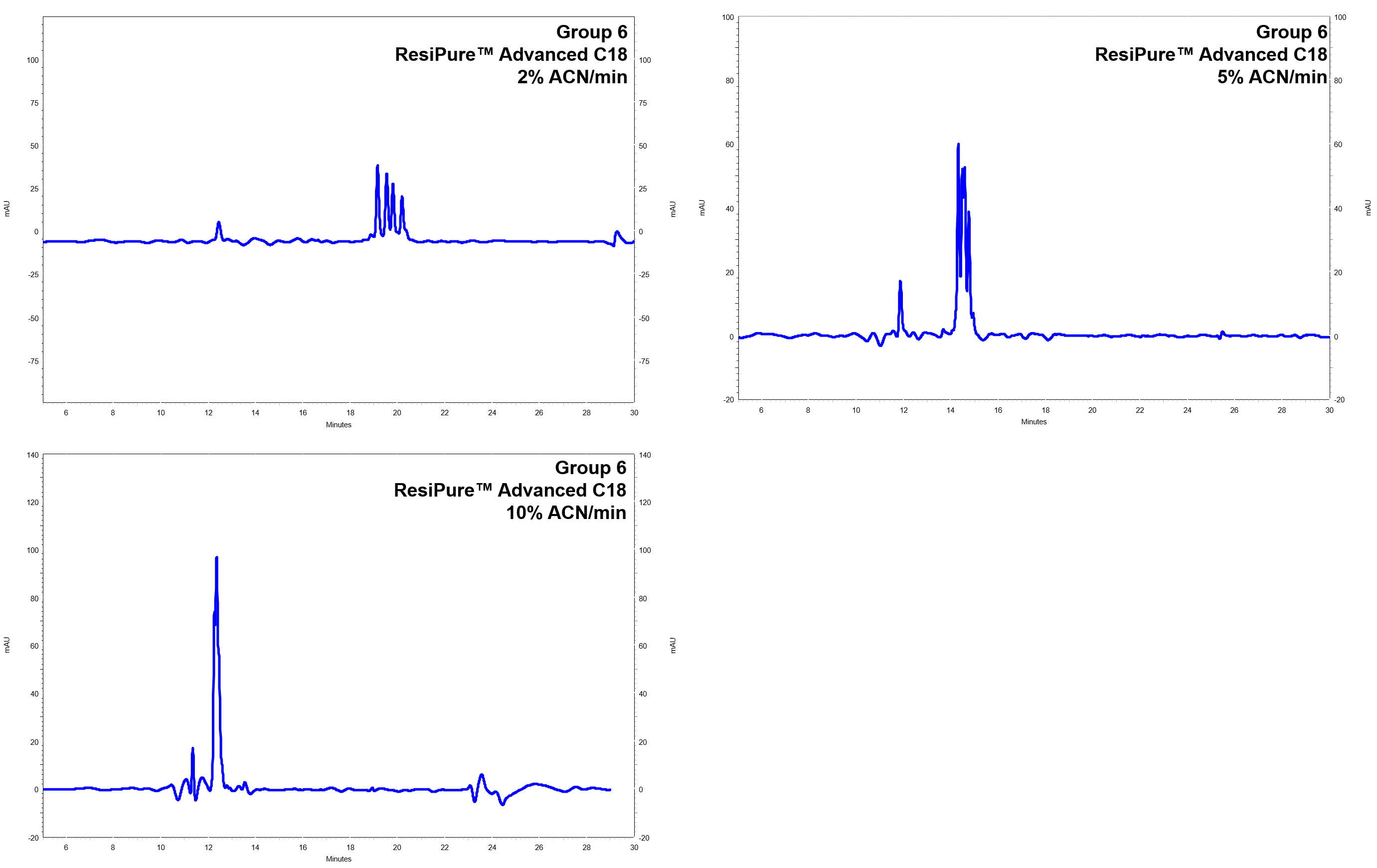


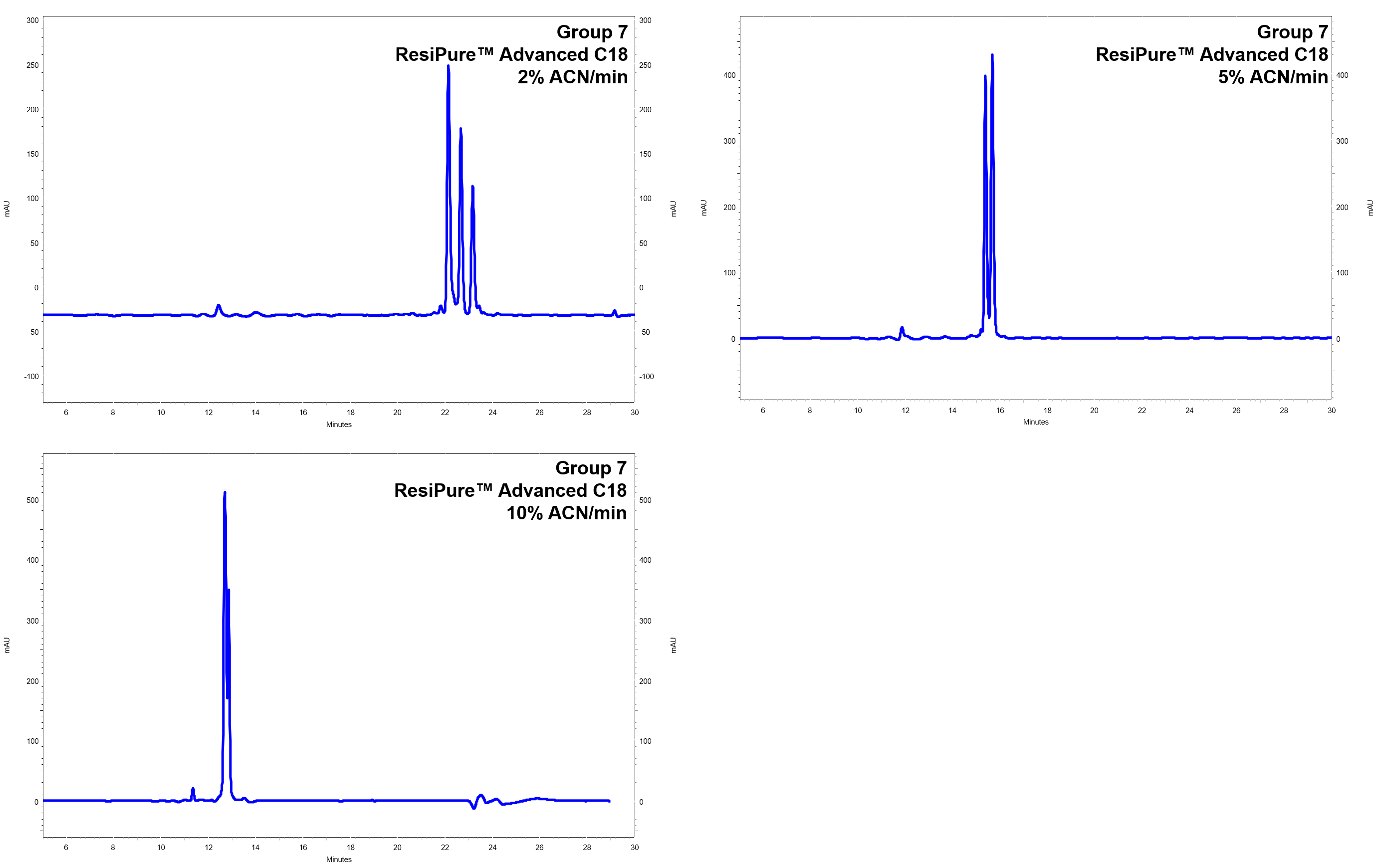


Figure SI-3.1: RP-HPLC-UV chromatograms of groups 1-7 with varying gradient steepness measured on the ResiPure™ Advanced C18


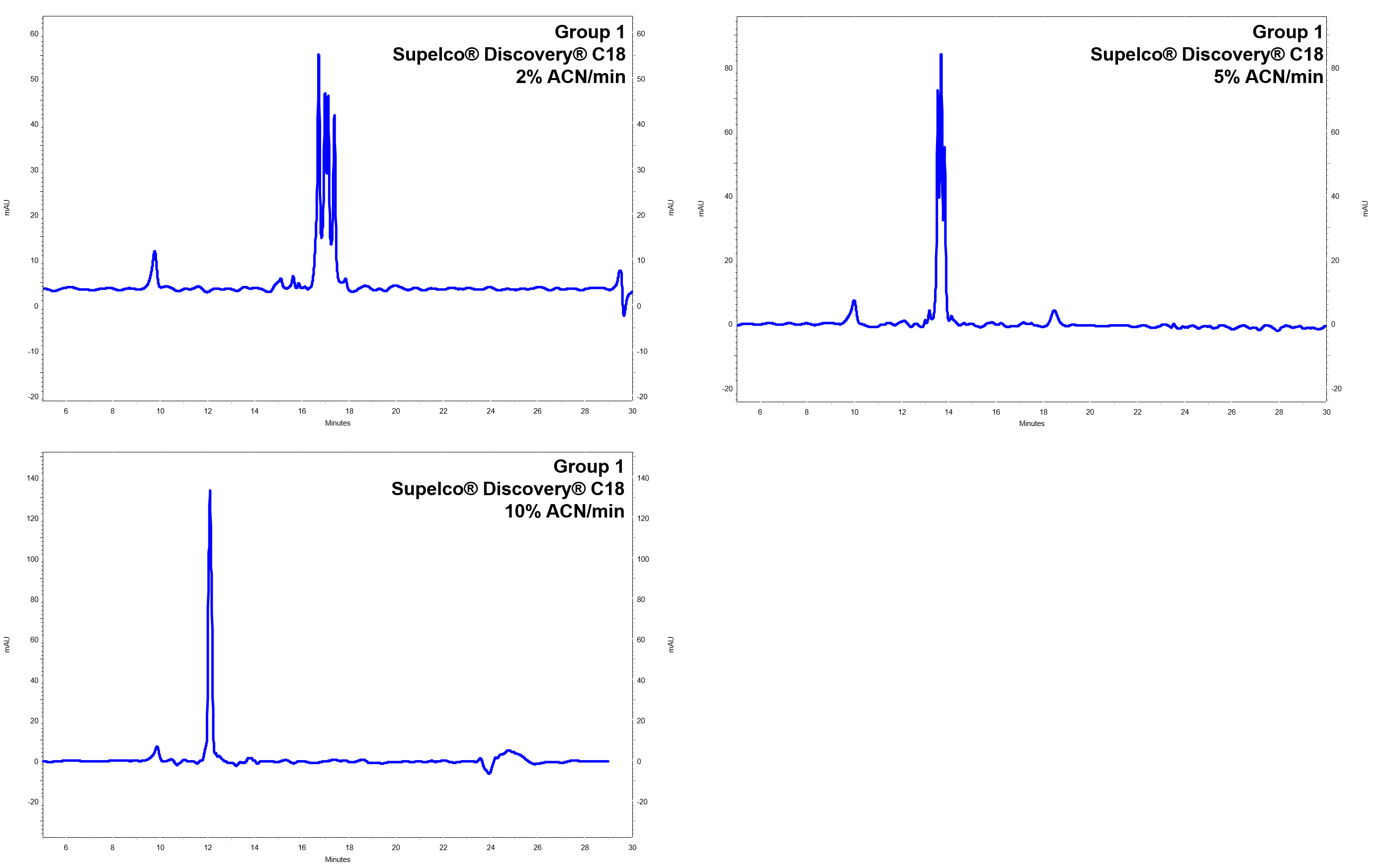


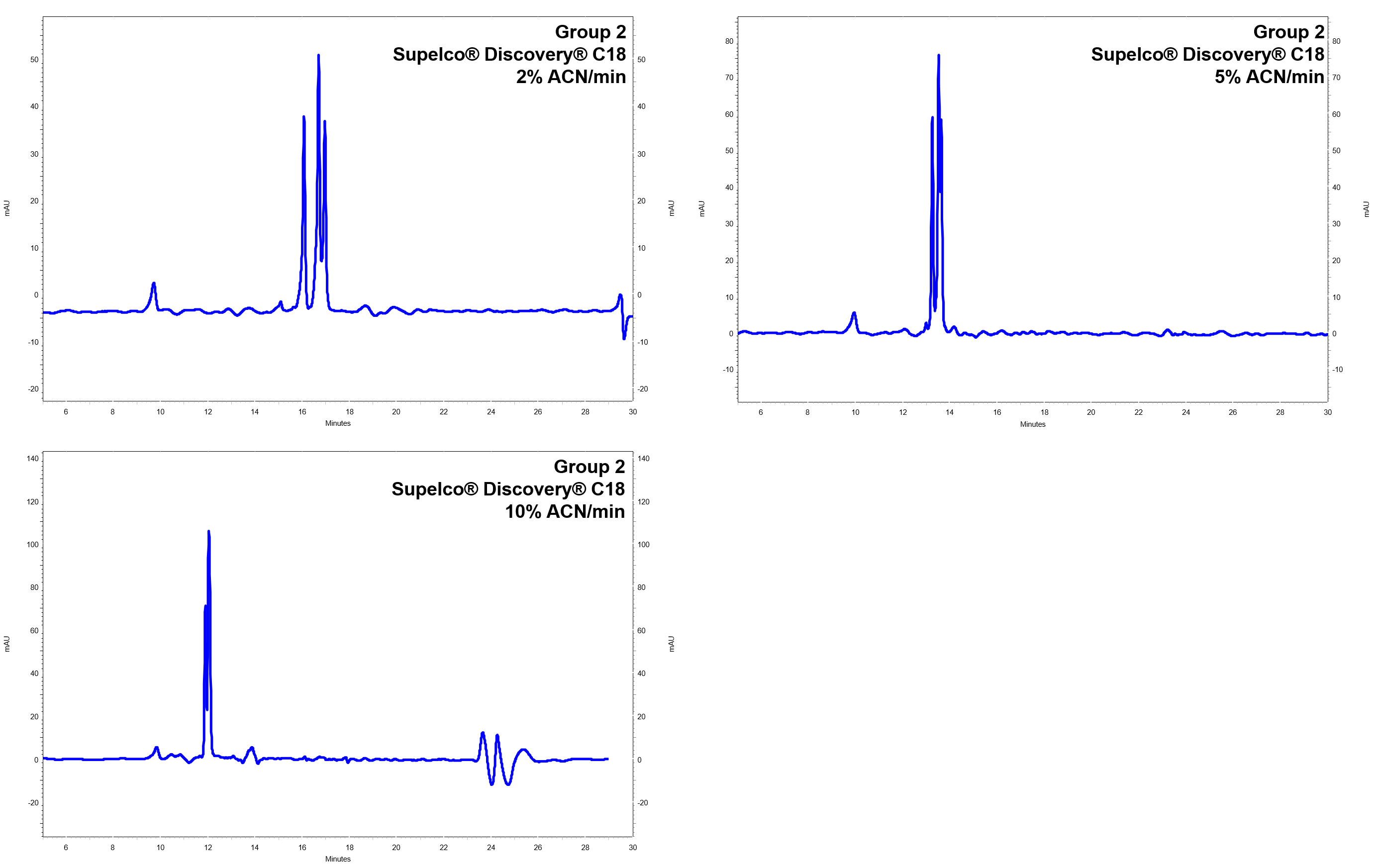


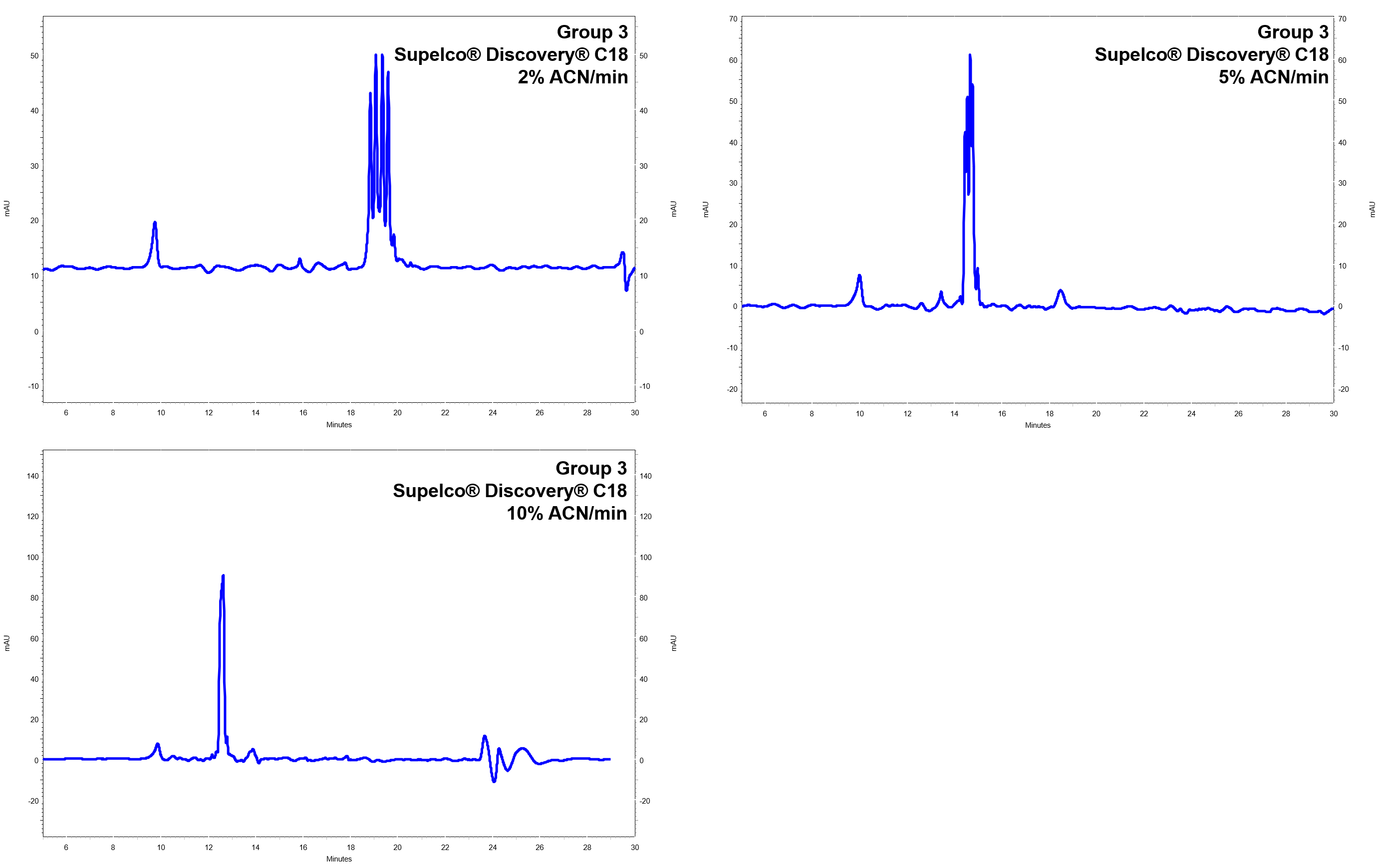


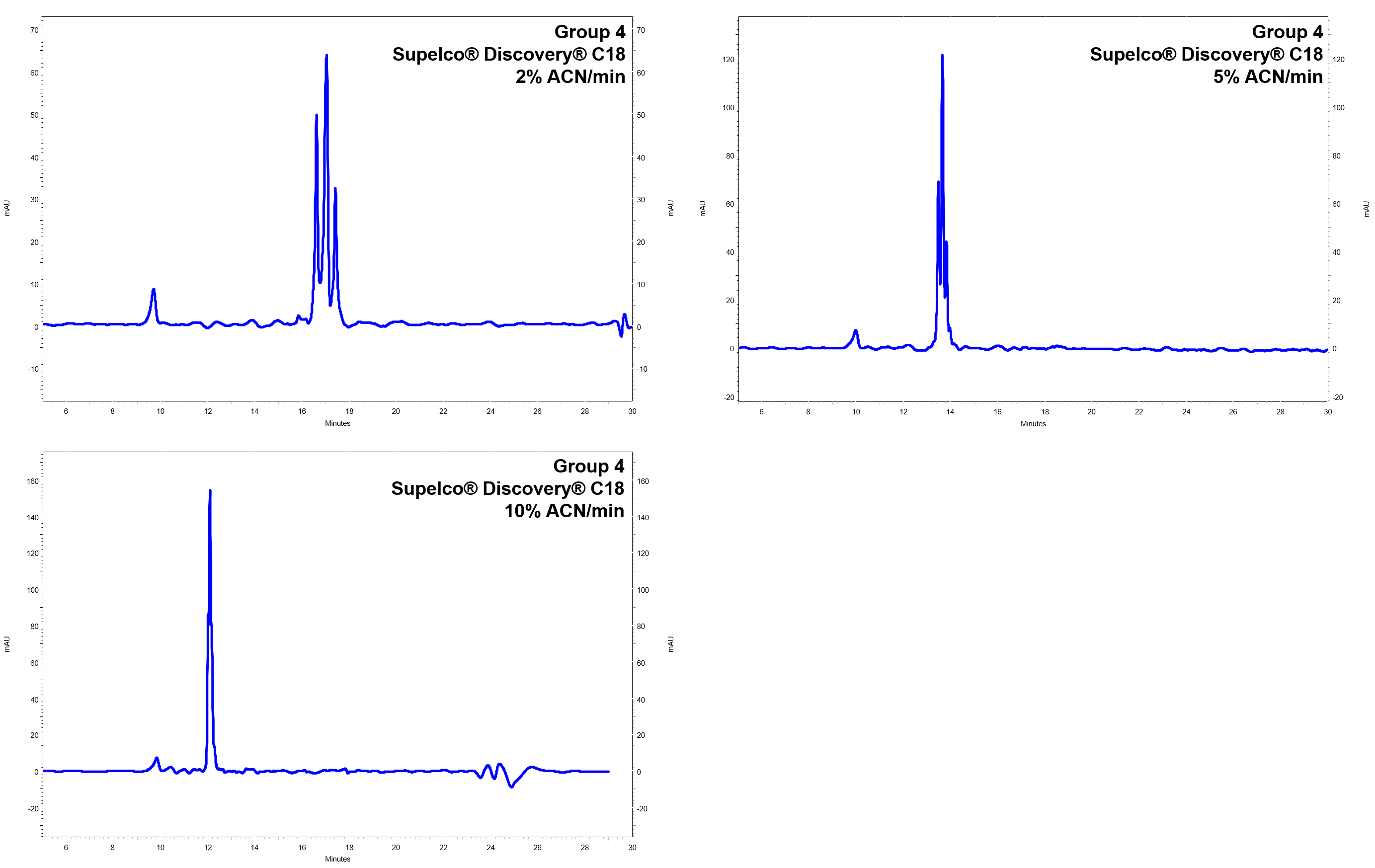


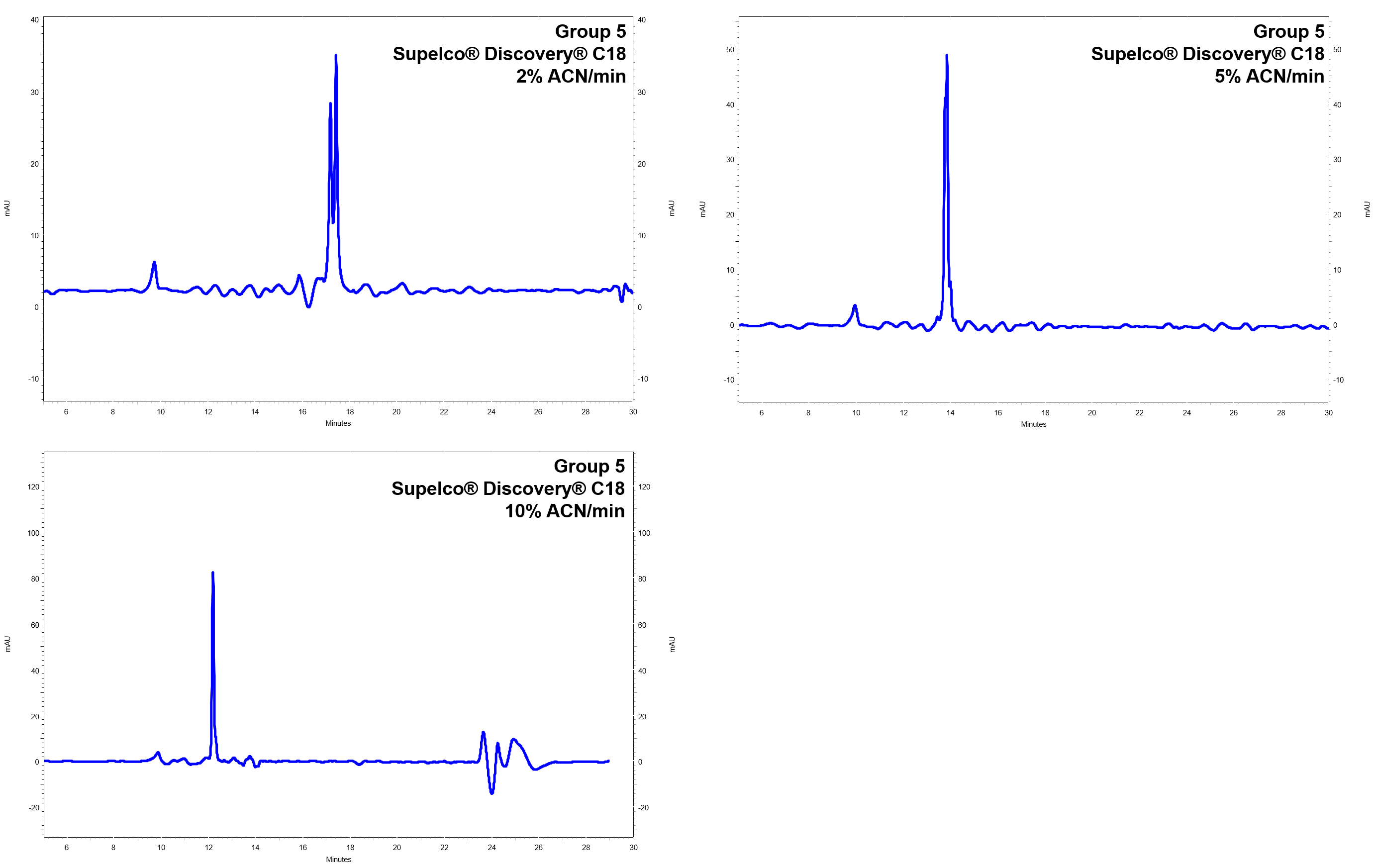


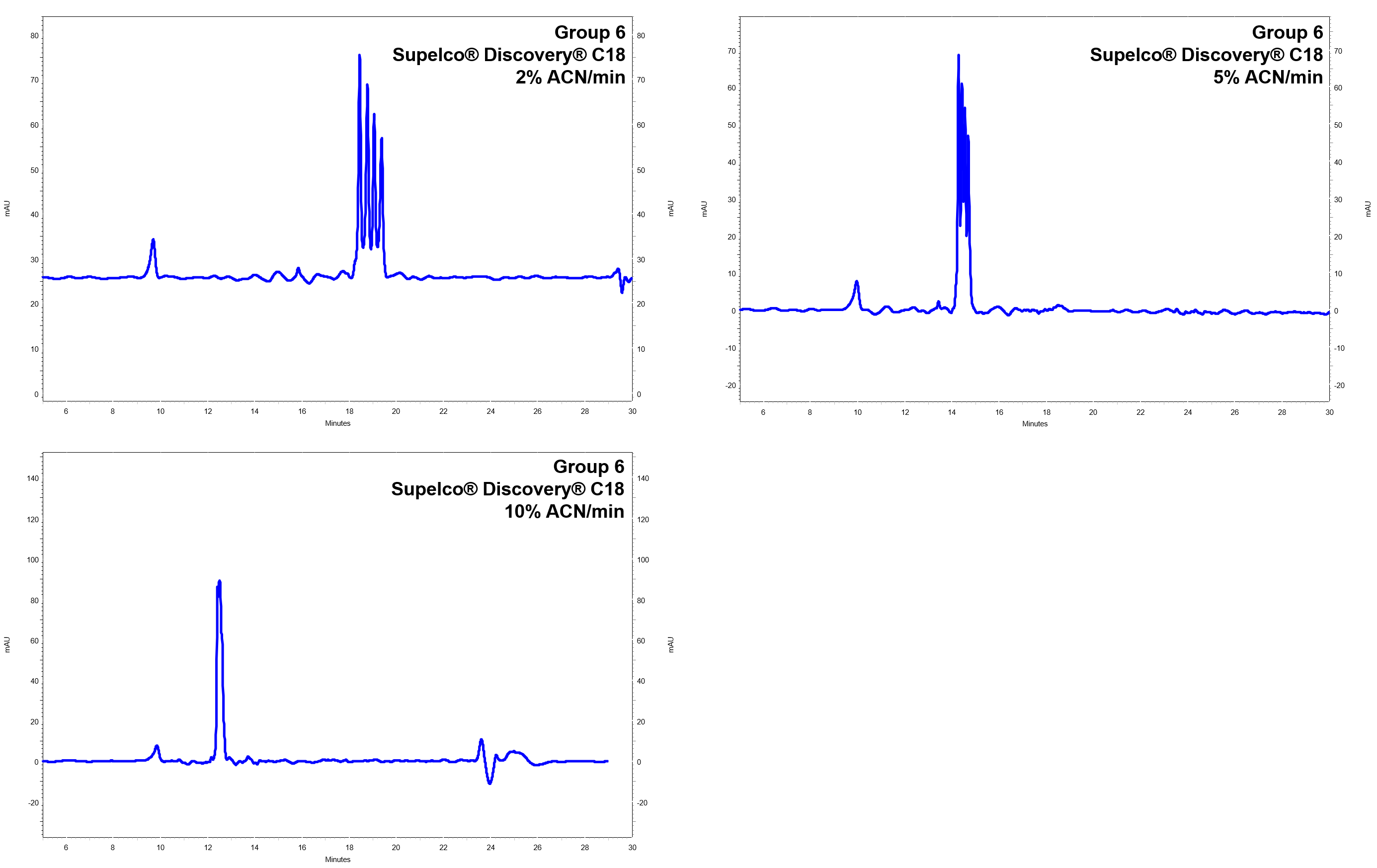


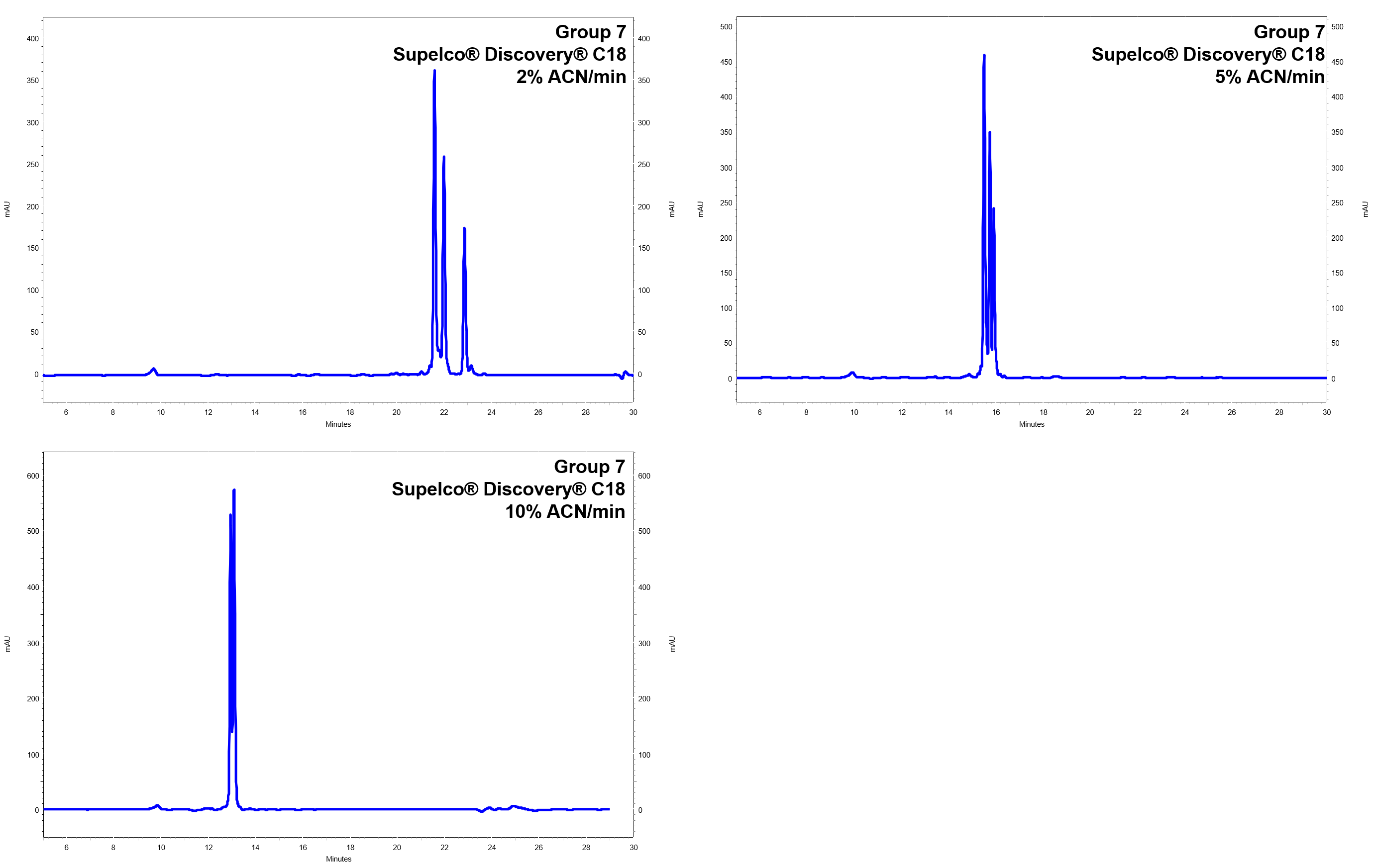


Figure SI-3.2: RP-HPLC-UV chromatograms of groups 1-7 with varying gradient steepness measured on the Supelco® Discovery® C18.


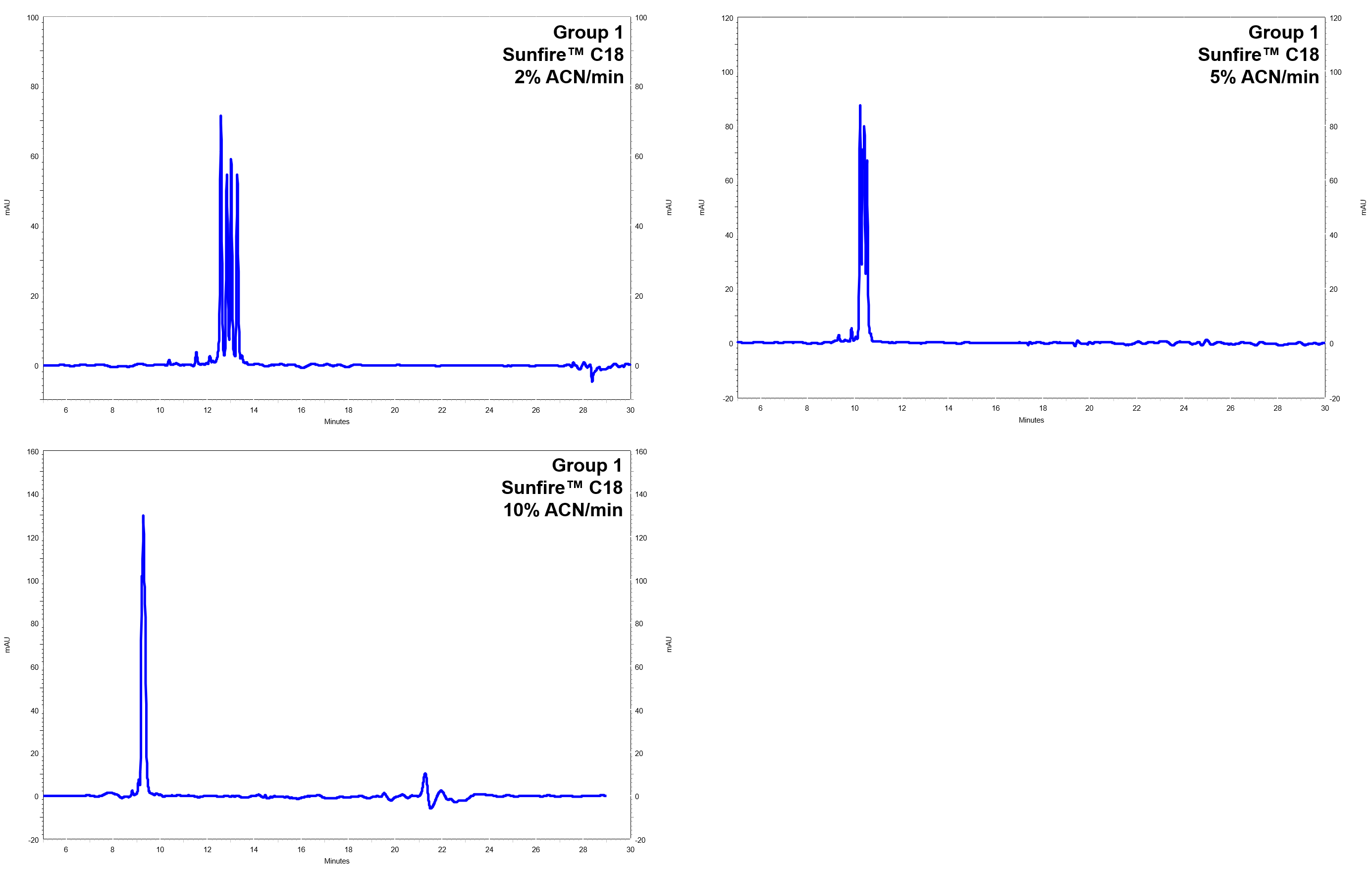


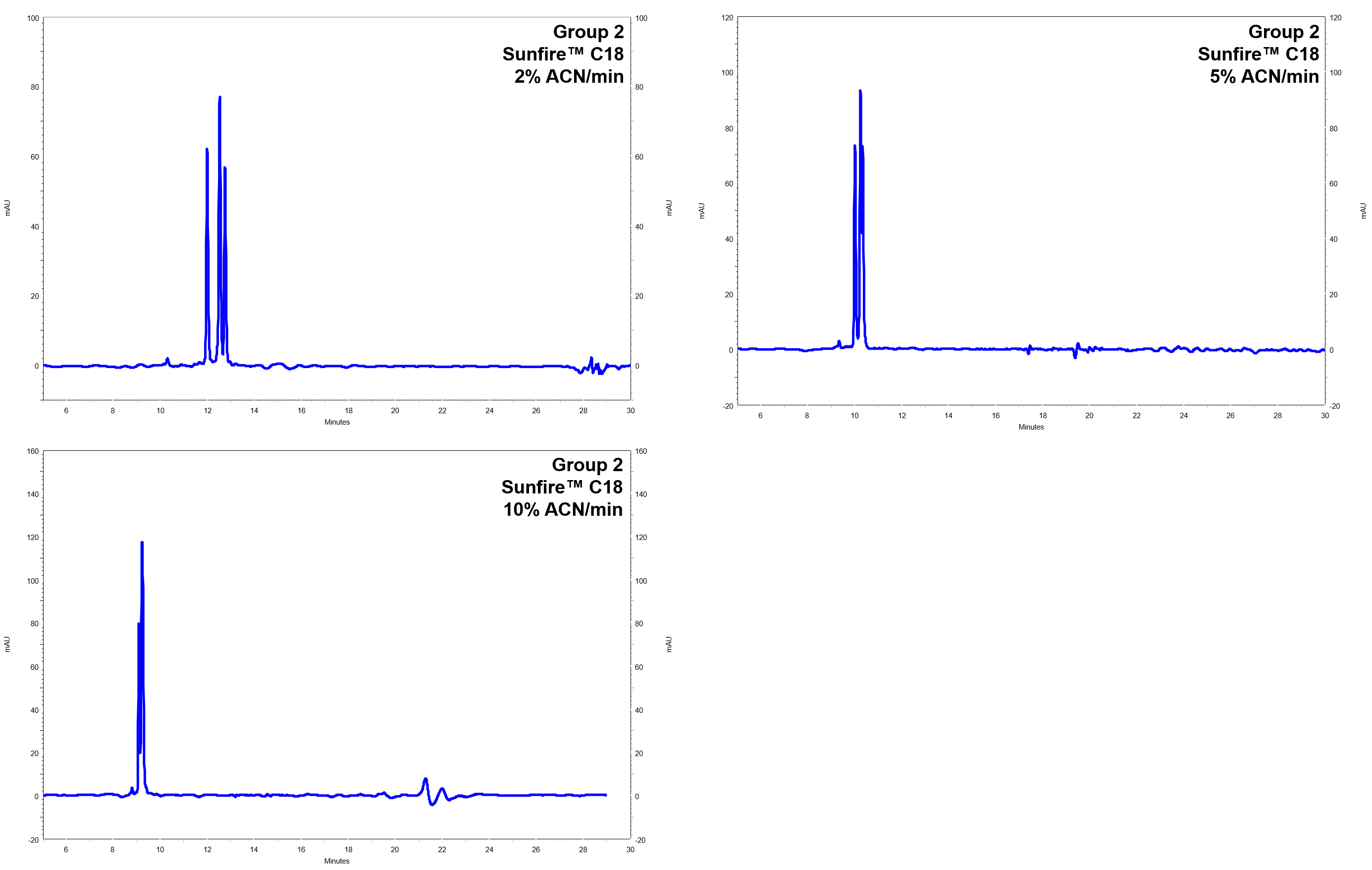


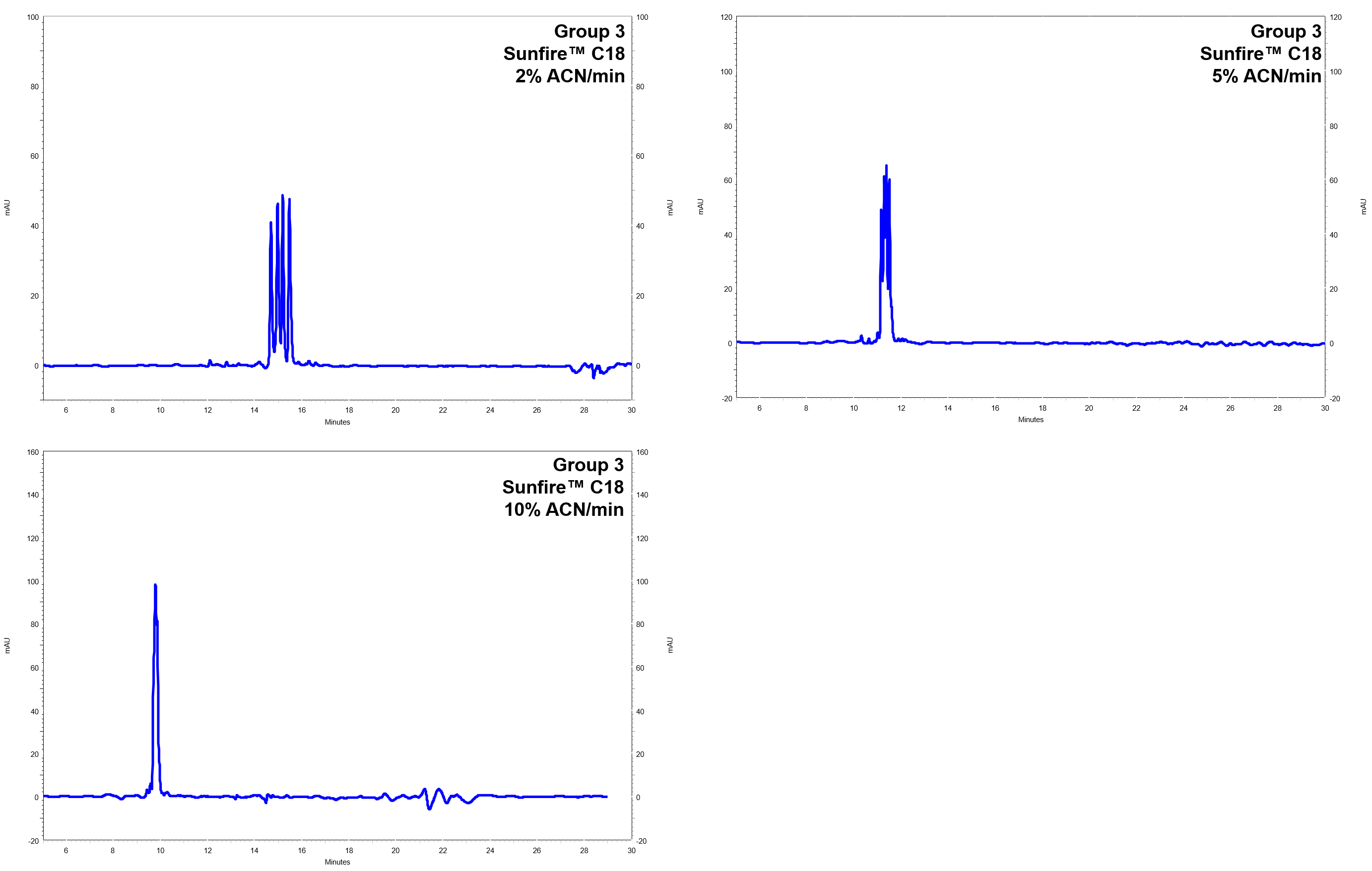


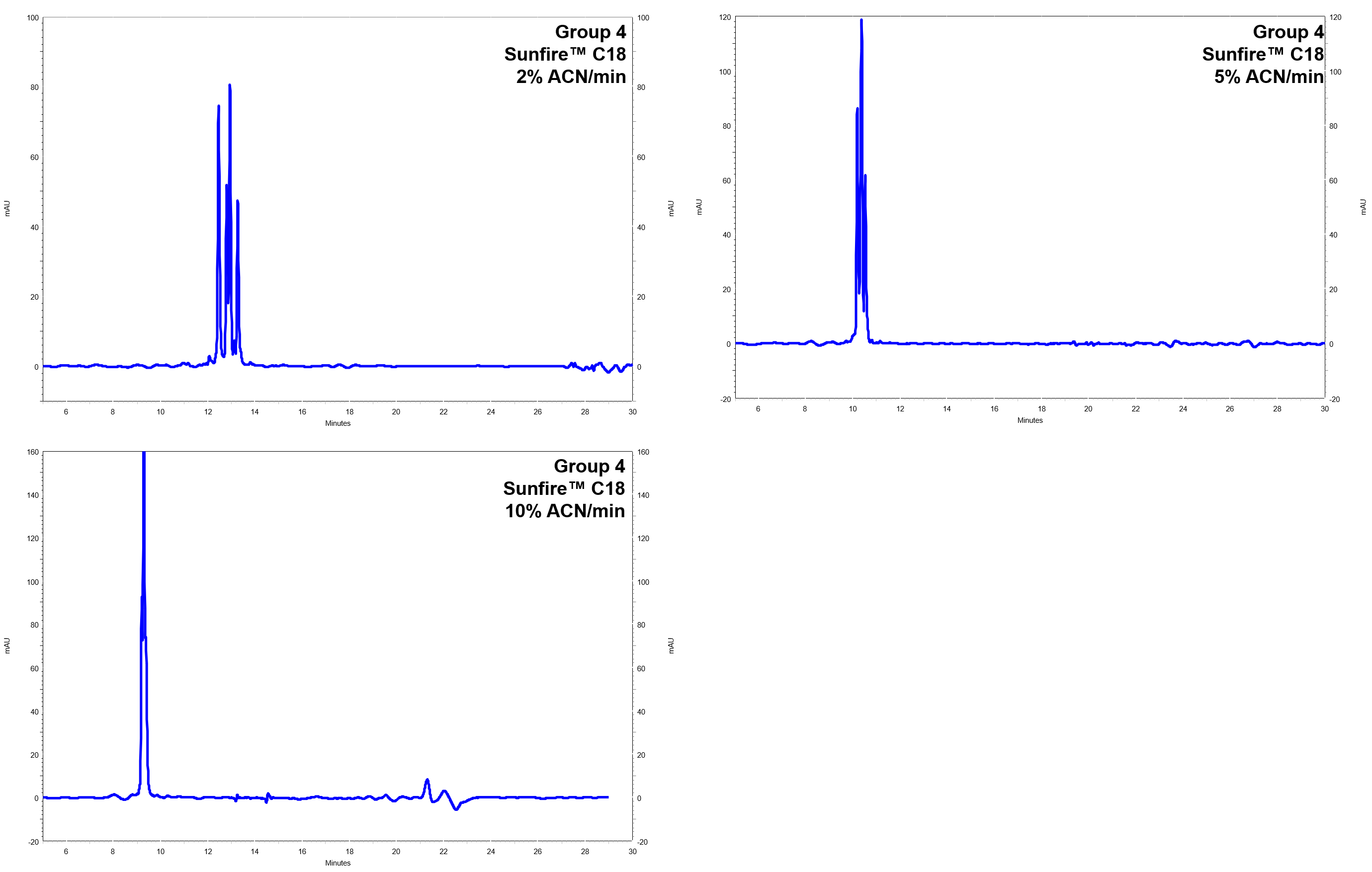


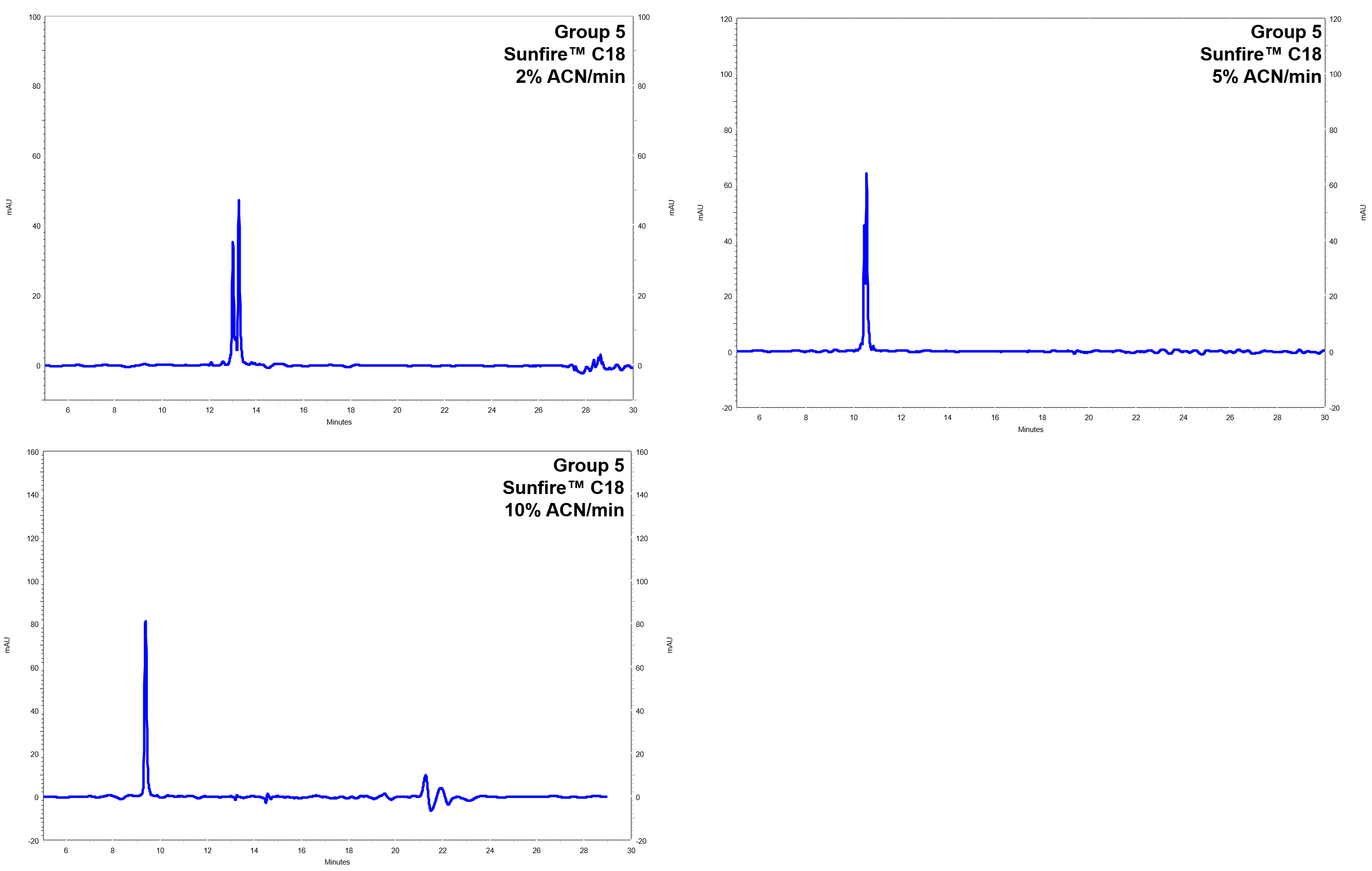


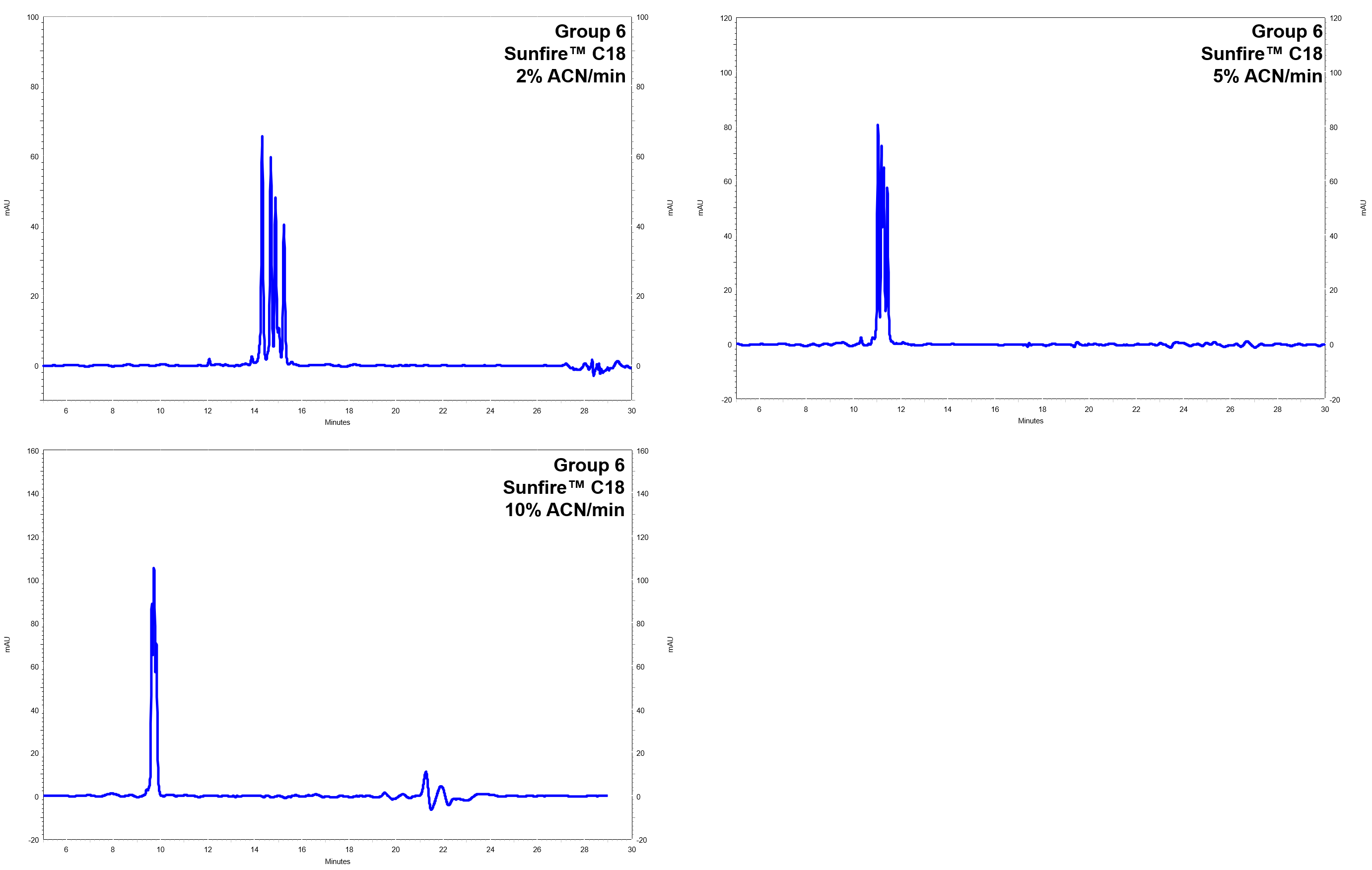


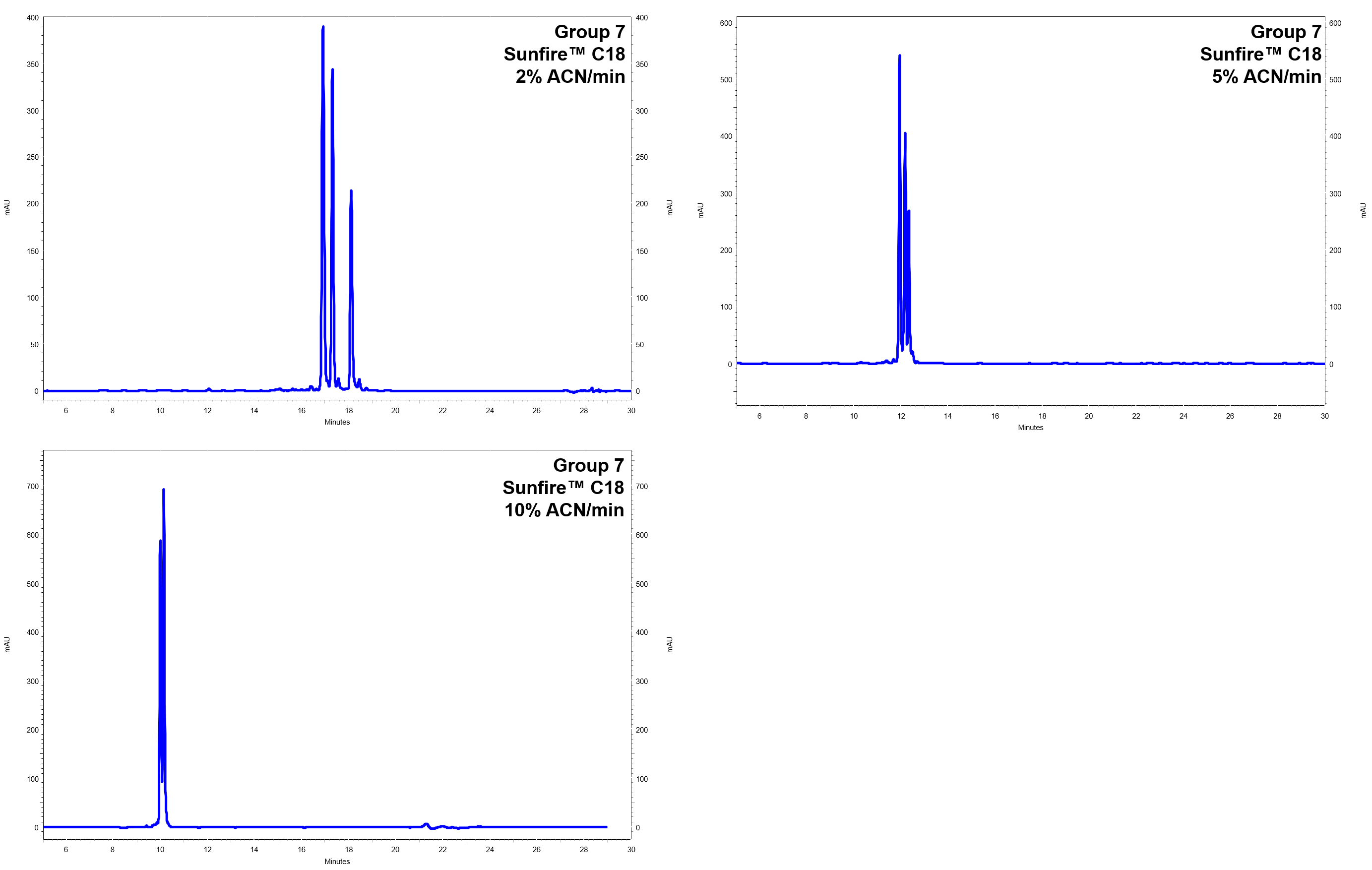


Figure SI-3.3: RP-HPLC-UV chromatograms of groups 1-7 with varying gradient steepness measured on the Sunfire™ C18.

# SI-4: Individual chromatograms to test the effect of the flow rate on the separation efficacy


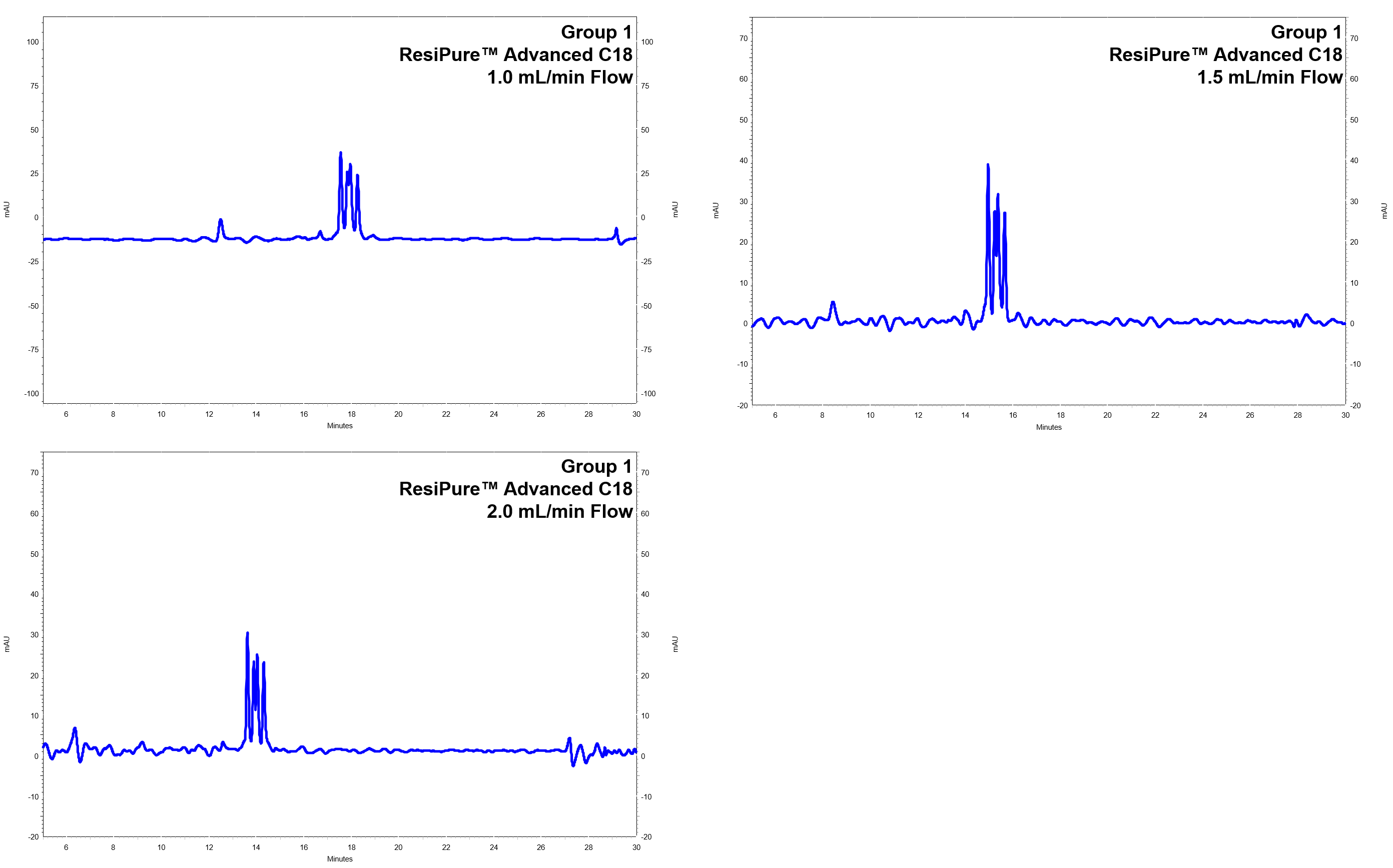


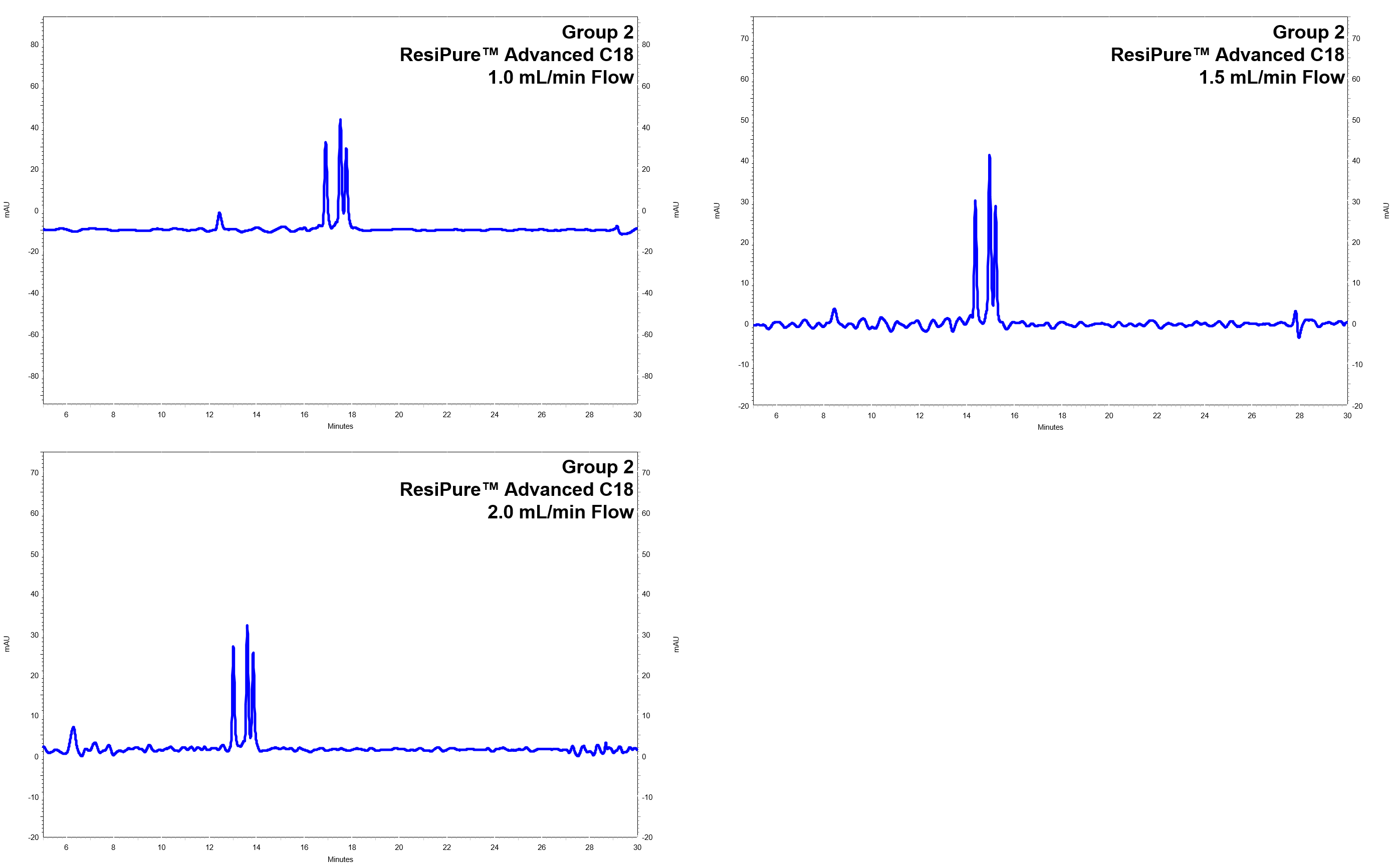


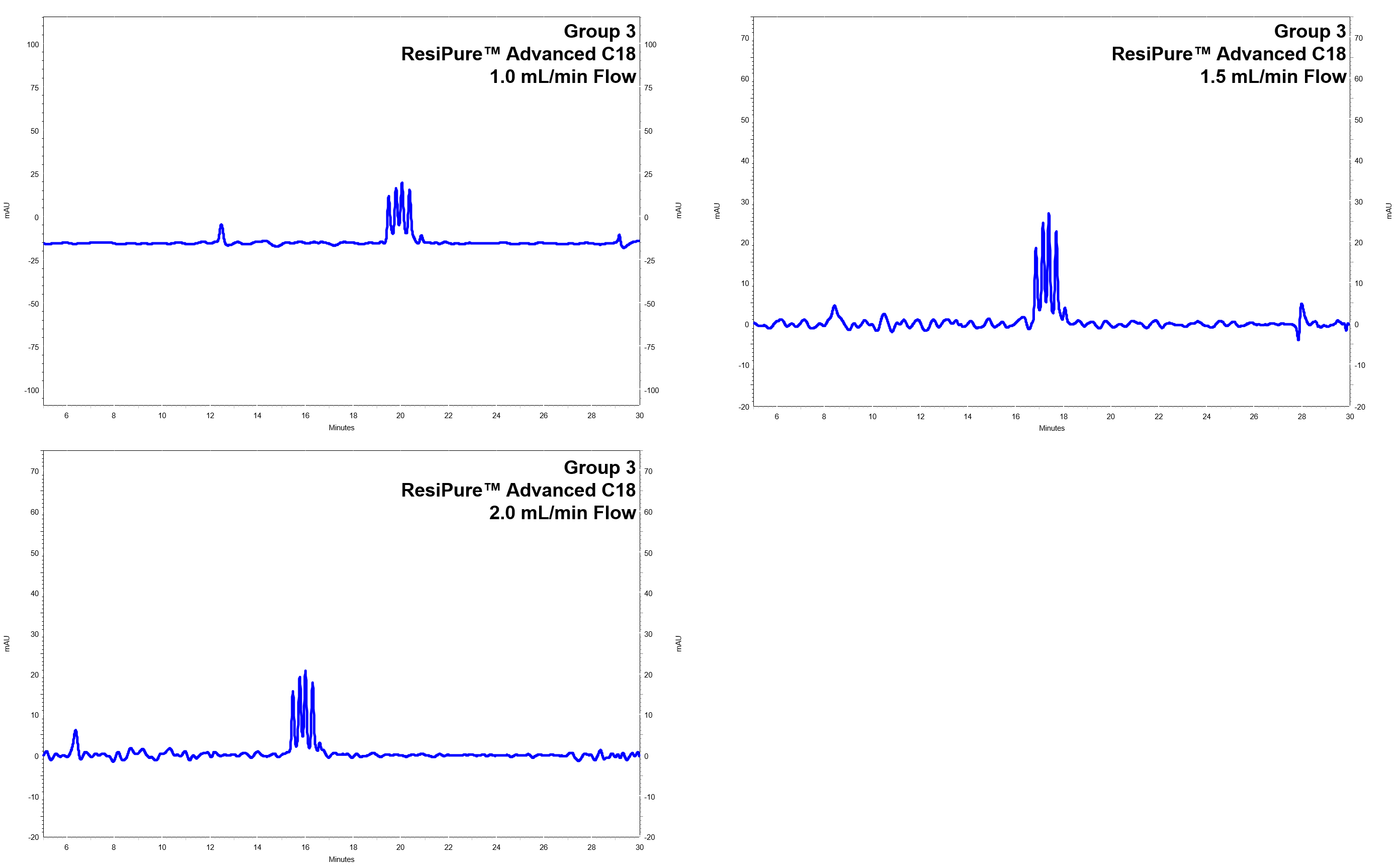


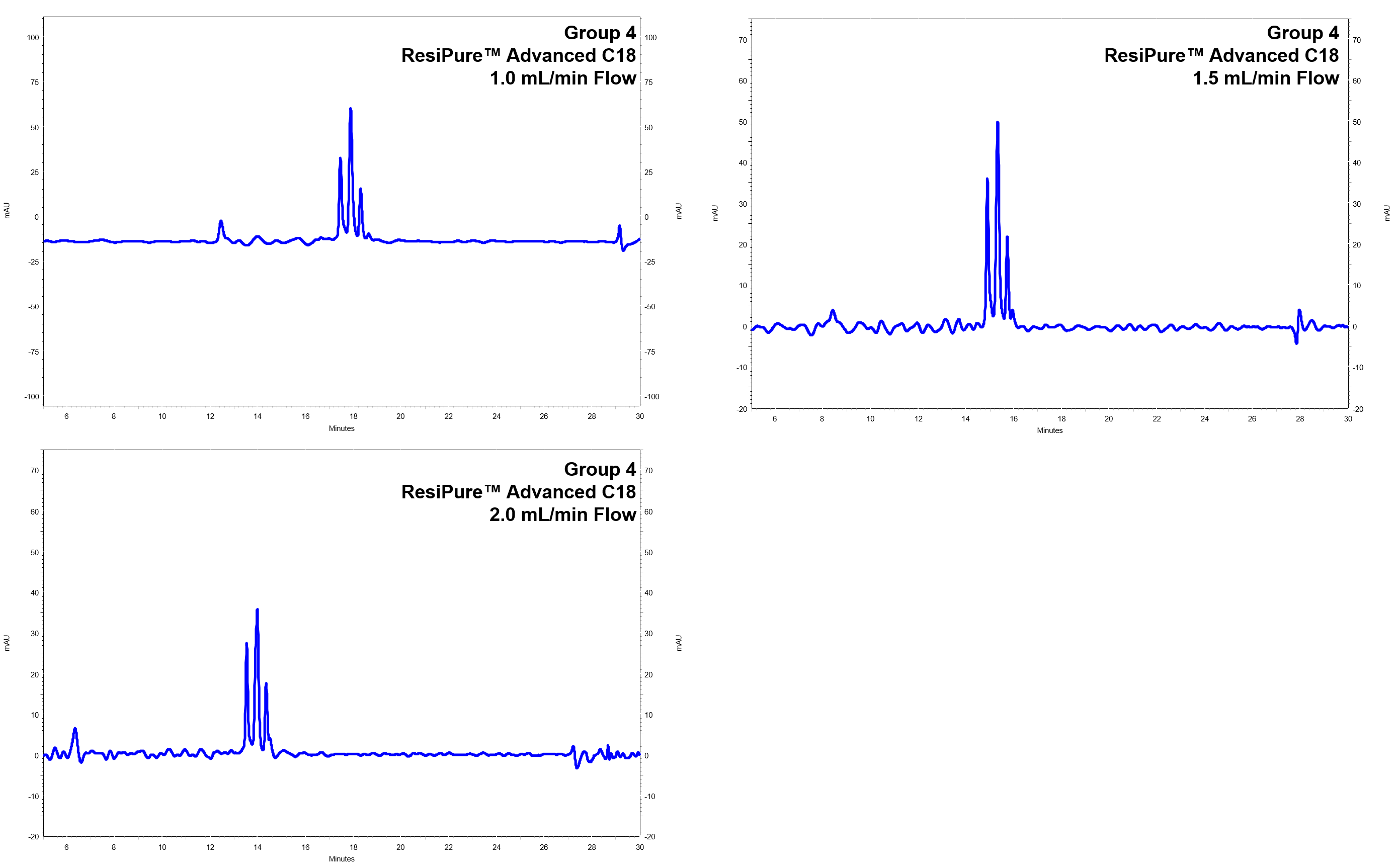


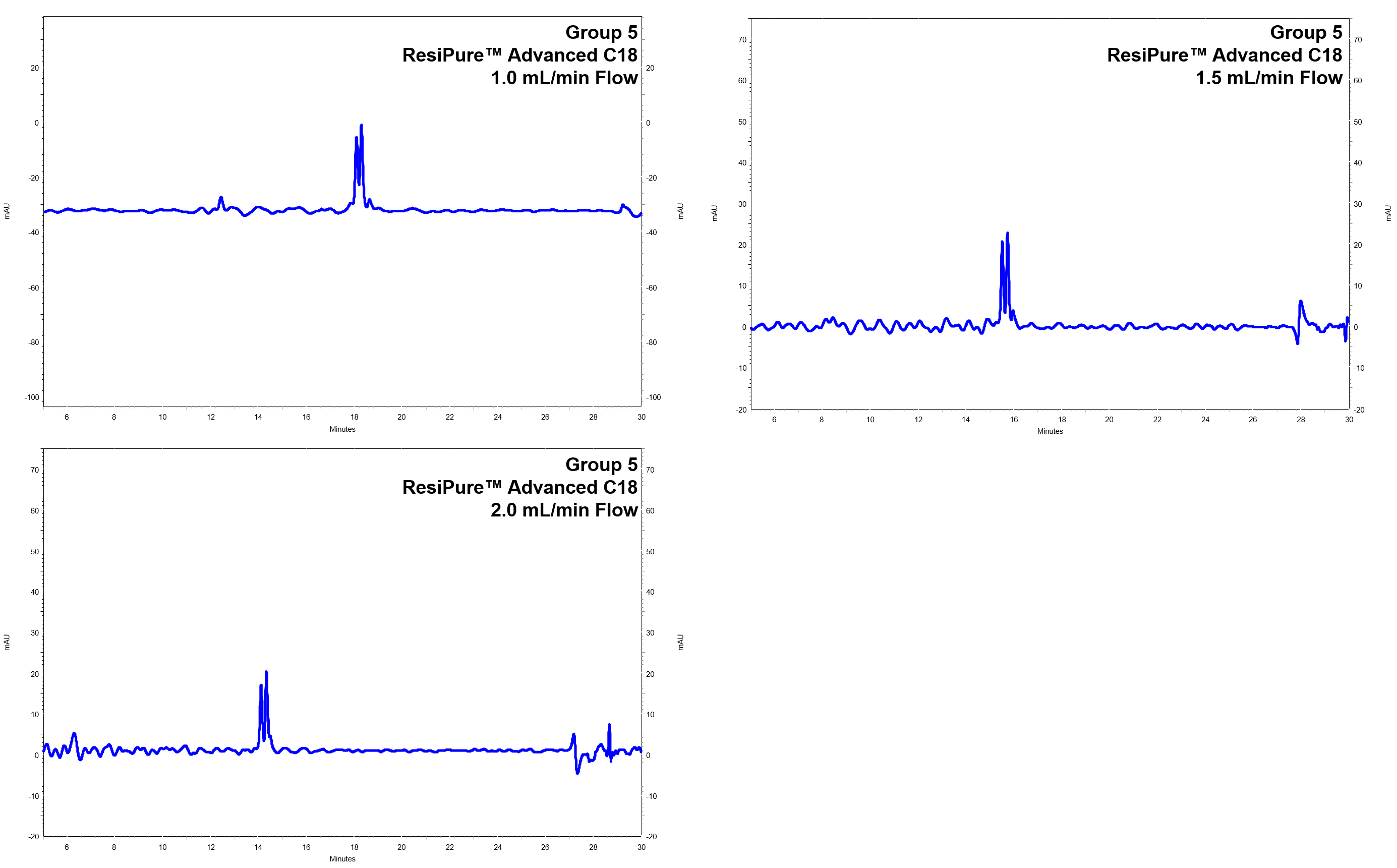


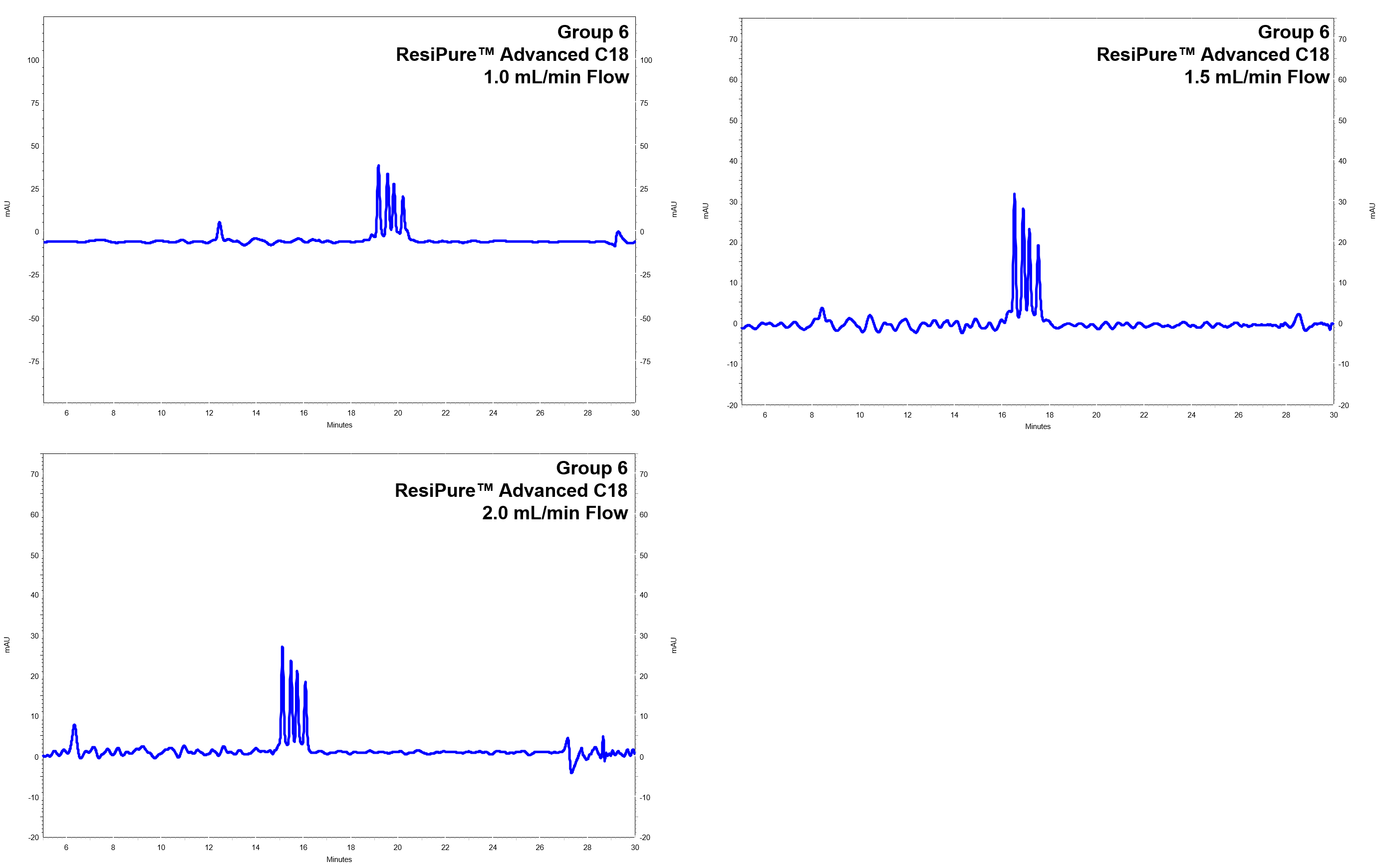


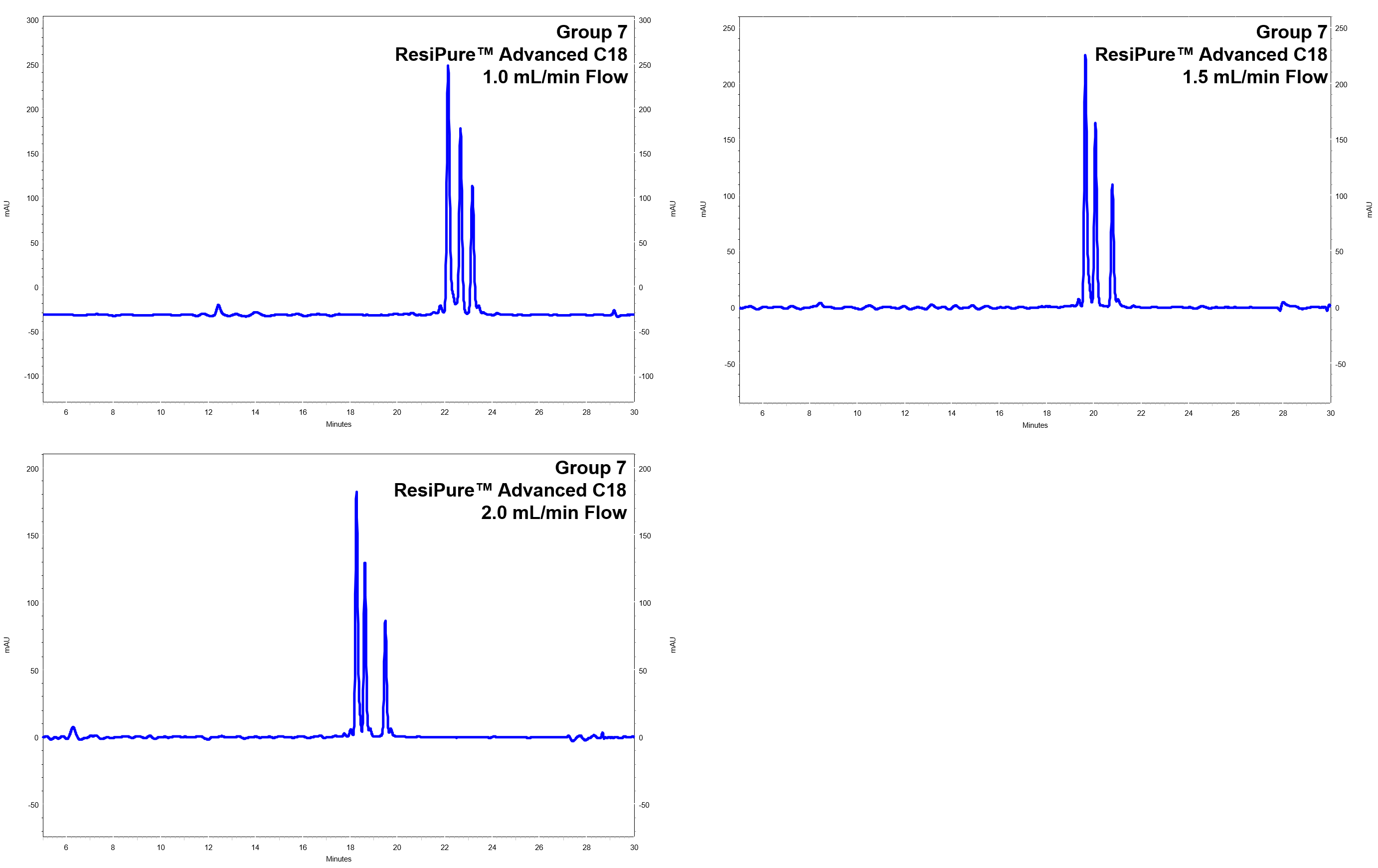


Figure SI-4.1: RP-HPLC-UV chromatograms of groups 1-7 with varying flow rates measured on the ResiPure™ Advanced C18


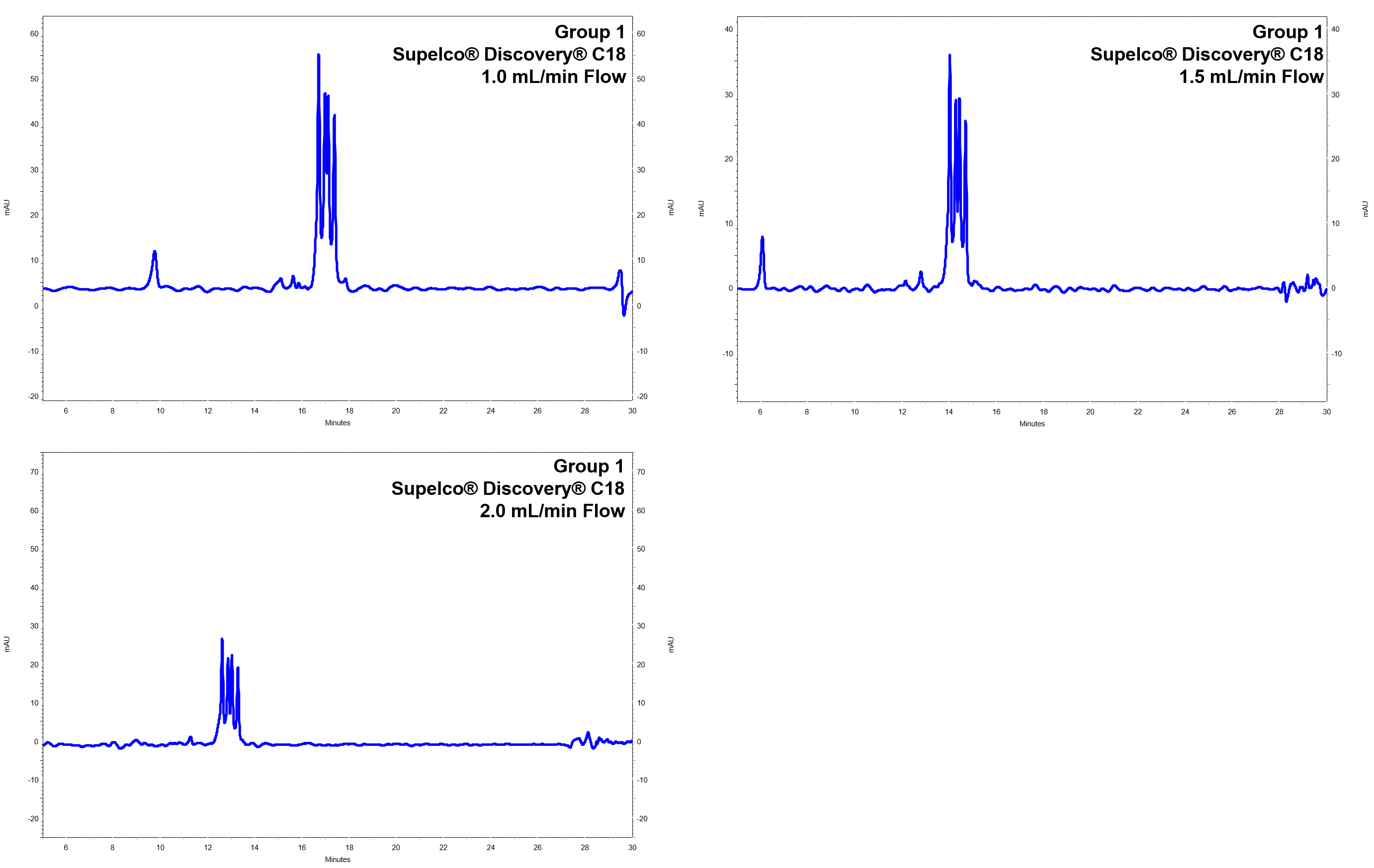


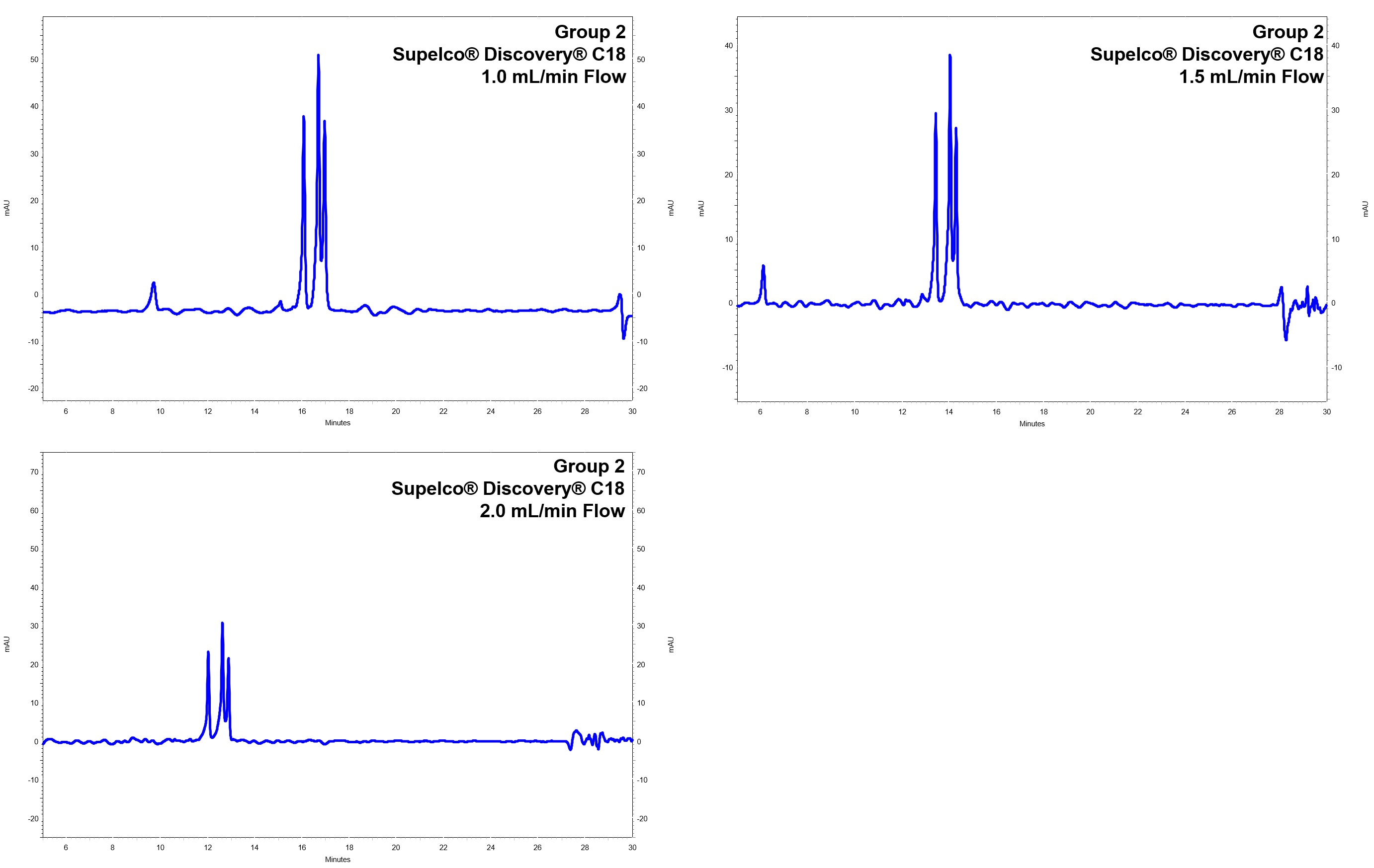


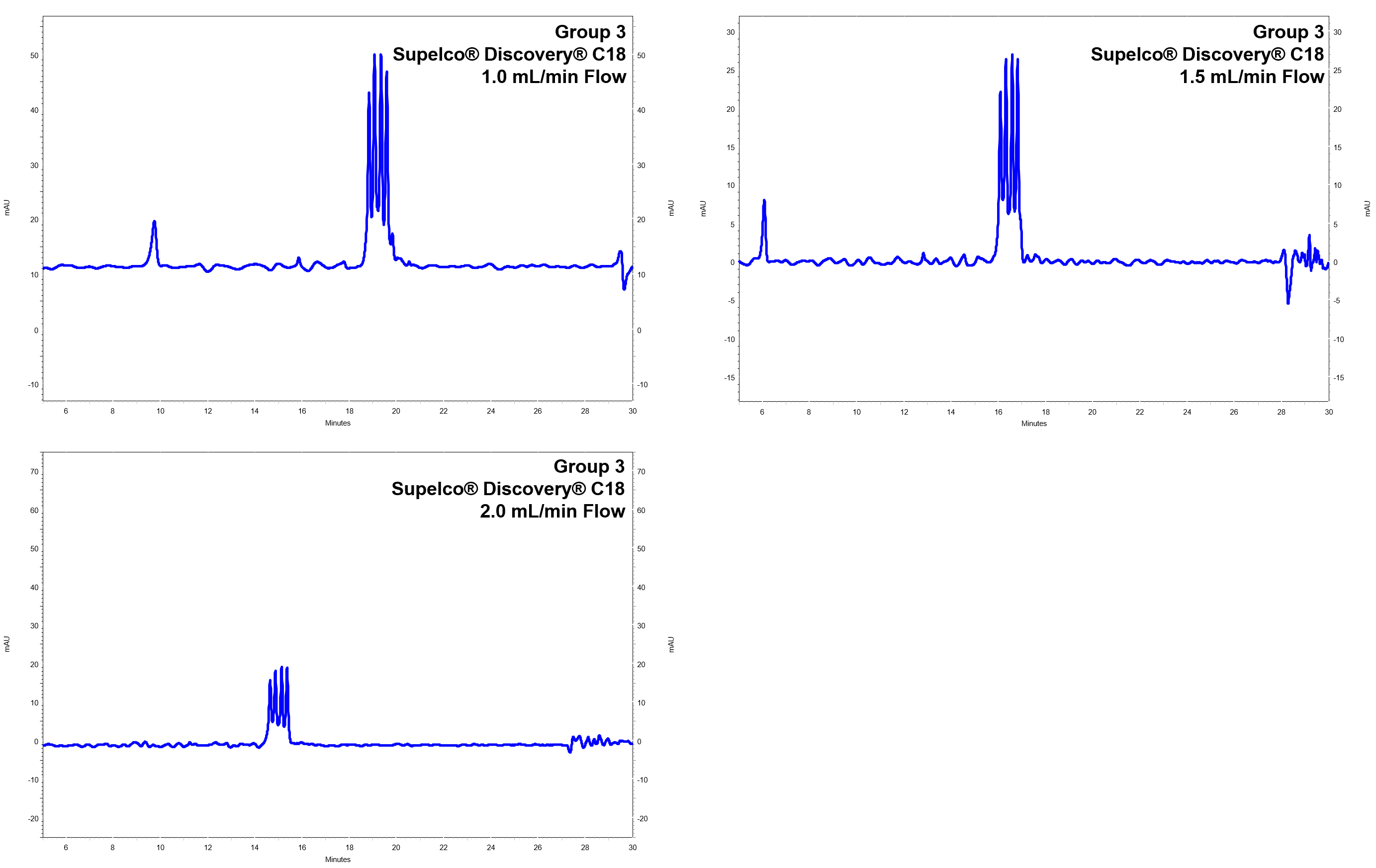


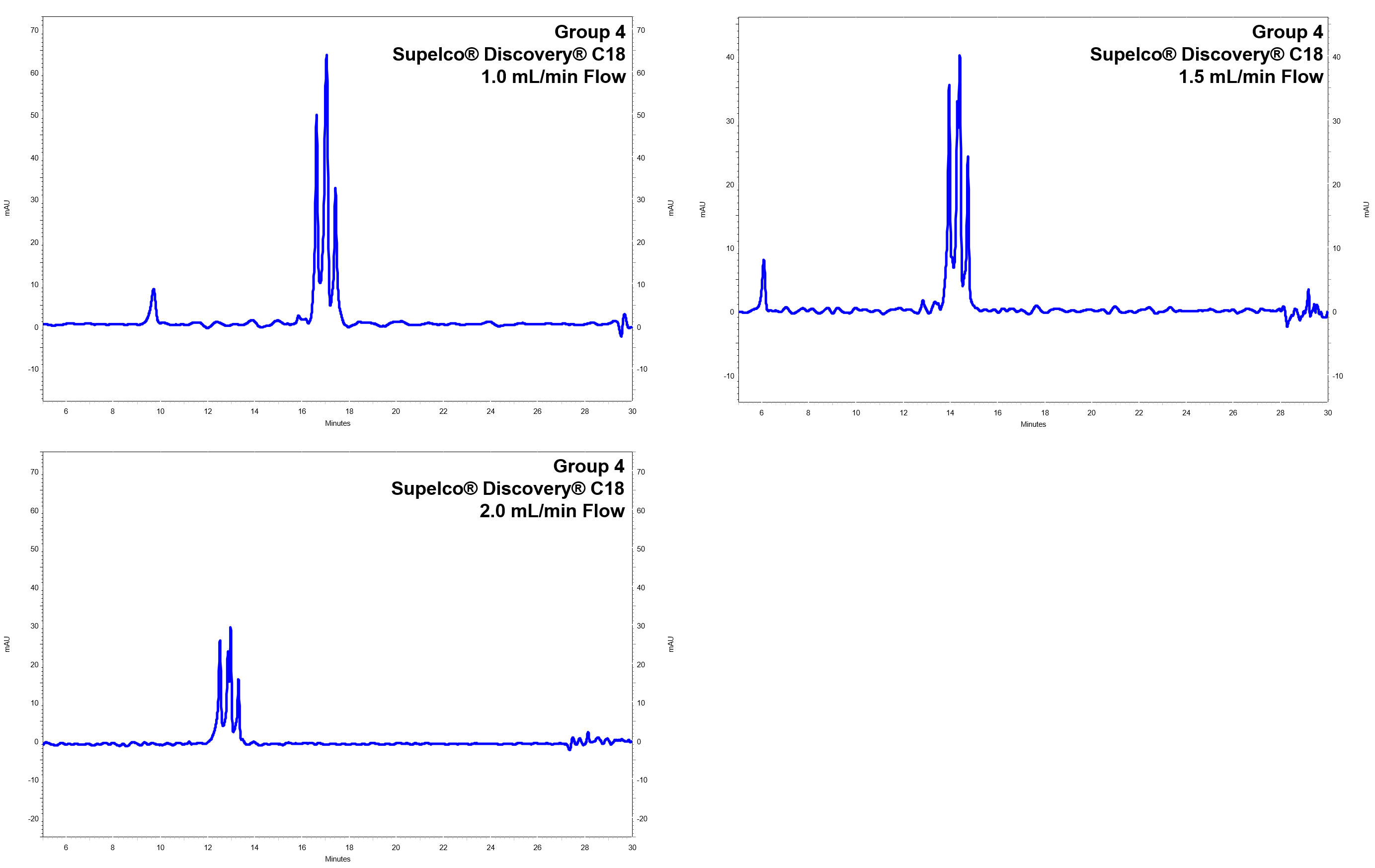


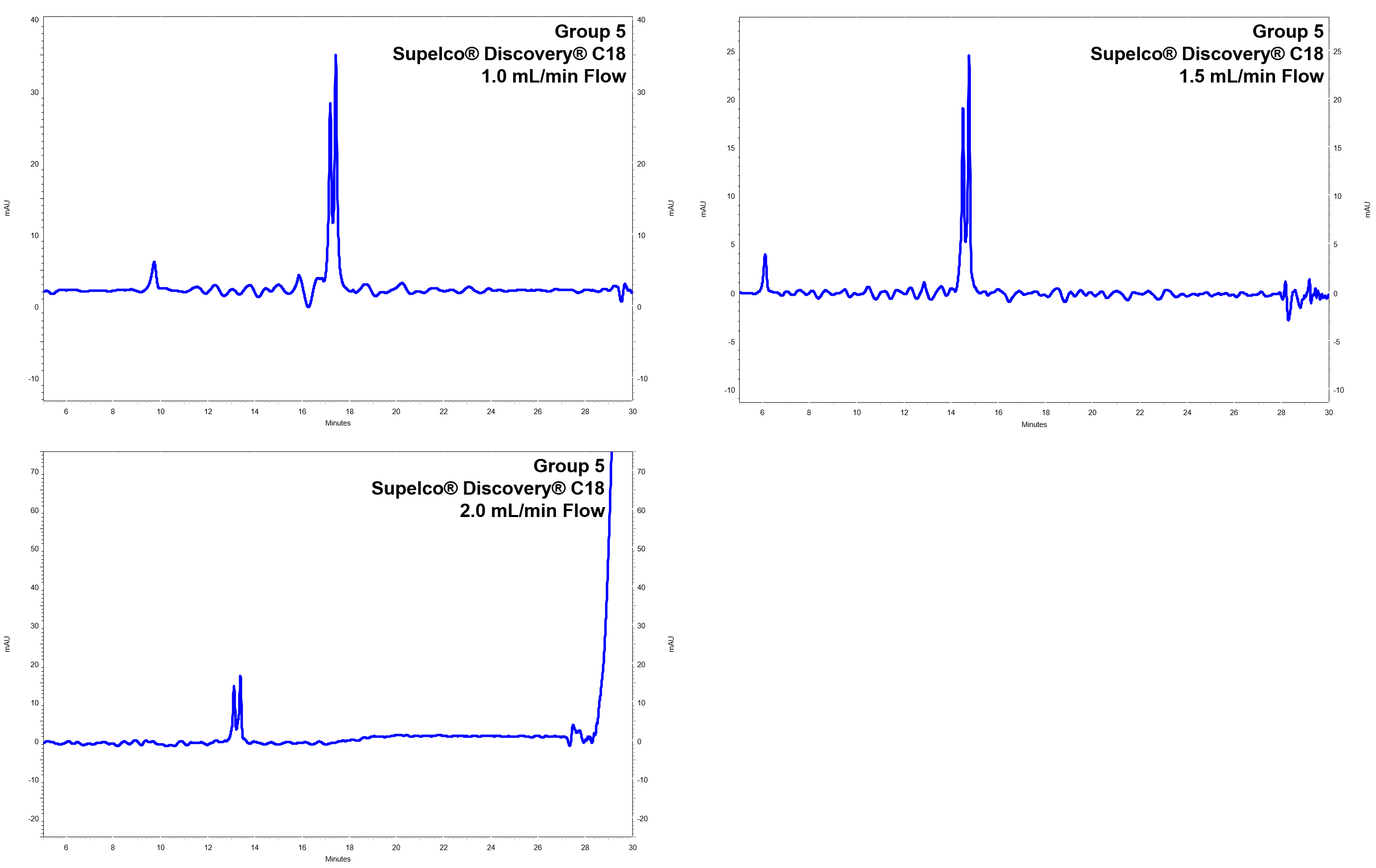


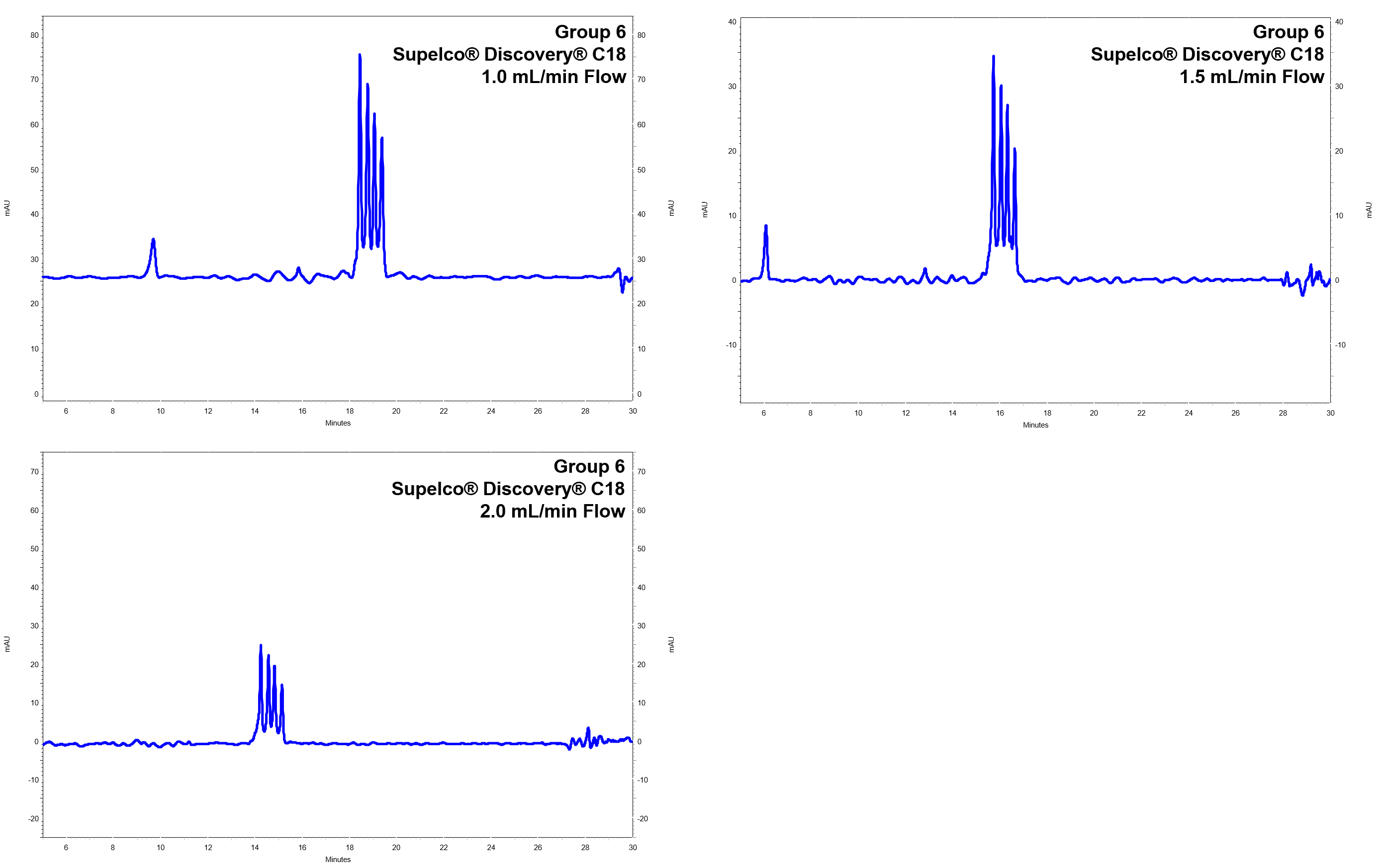


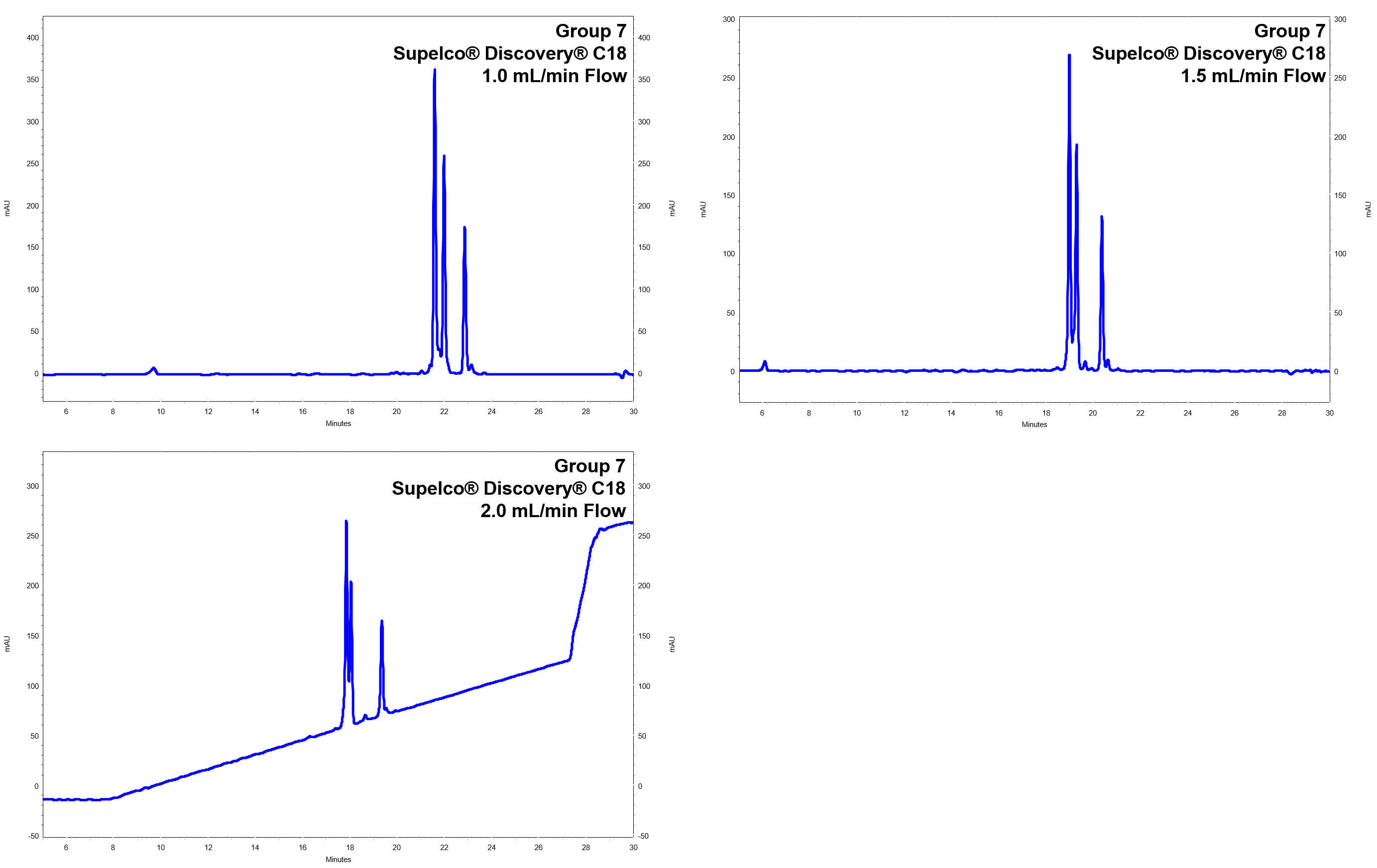


Figure SI-4.2: RP-HPLC-UV chromatograms of groups 1-7 with varying flow rates measured on the Supelco® Discovery® C18.

# SI-5: Individual chromatograms to test the effect of the temperature on the separation efficacy


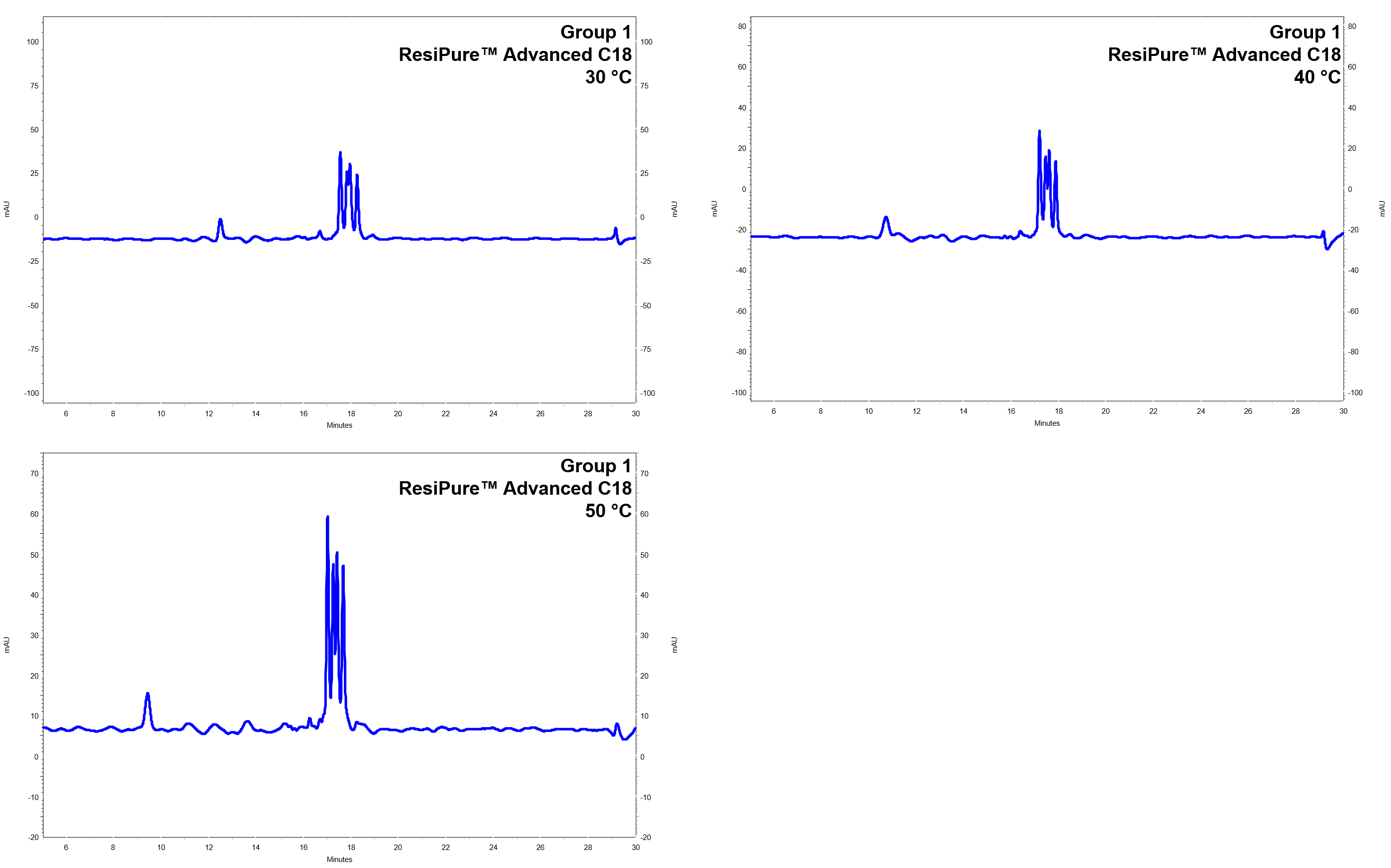


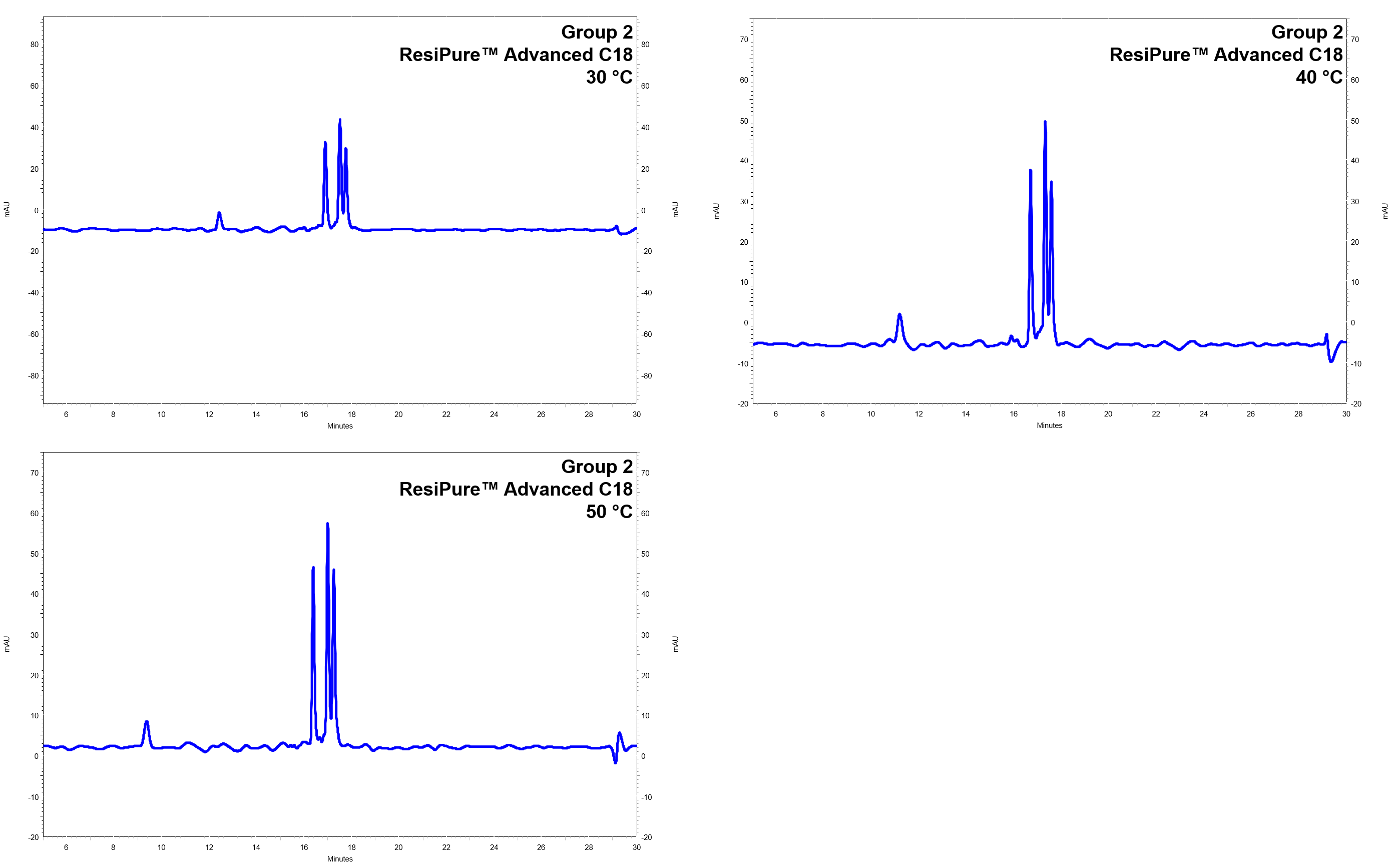


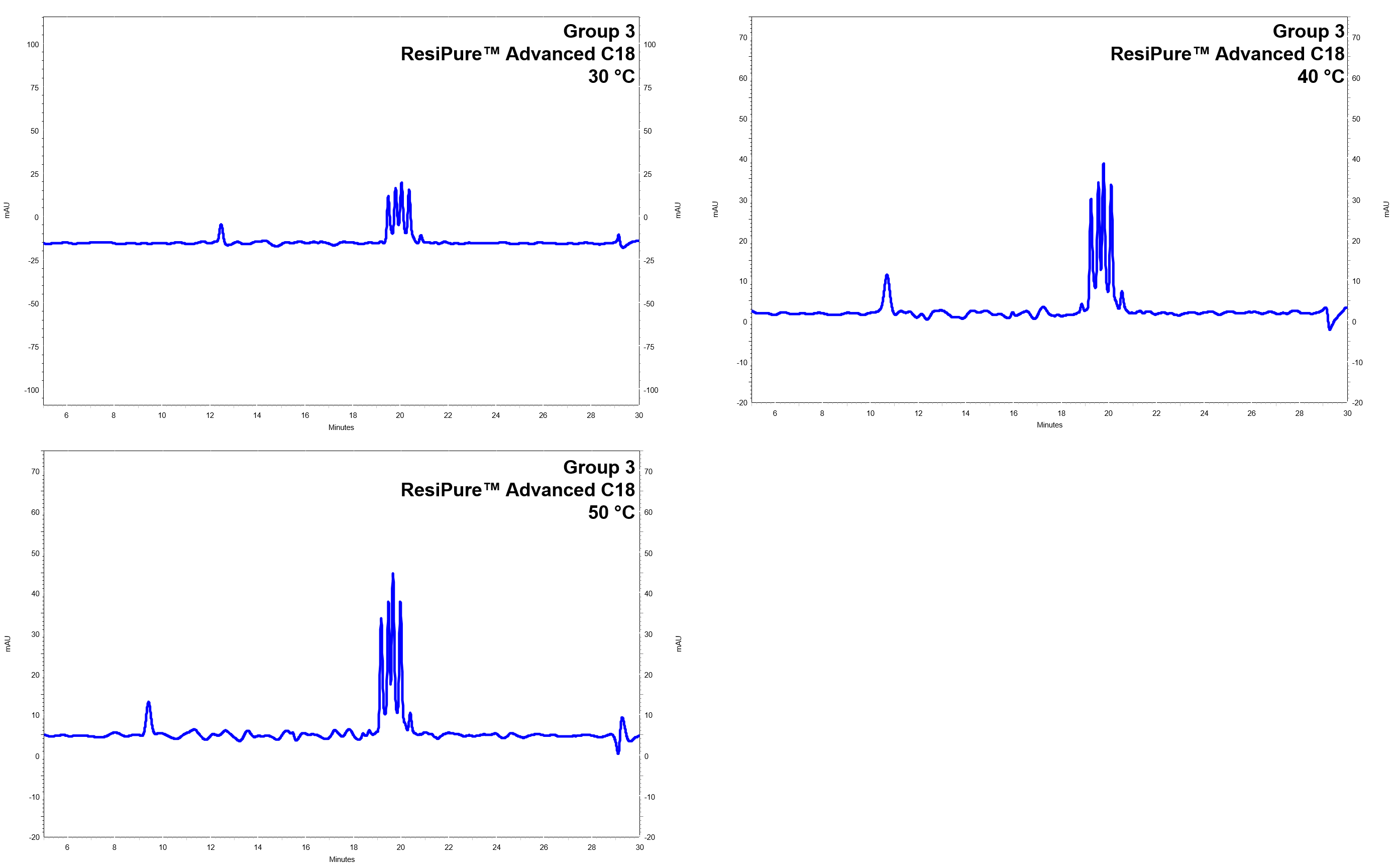


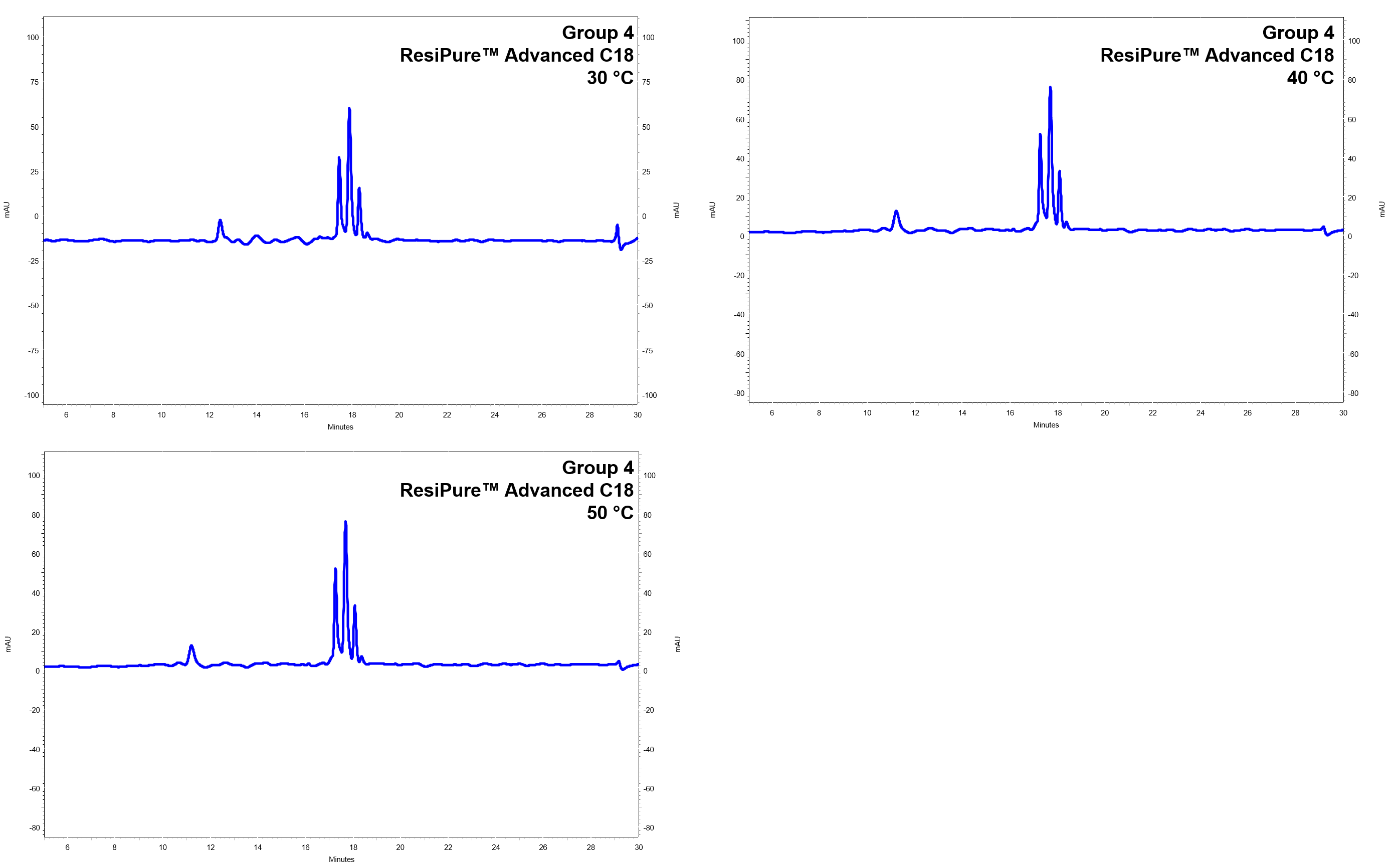


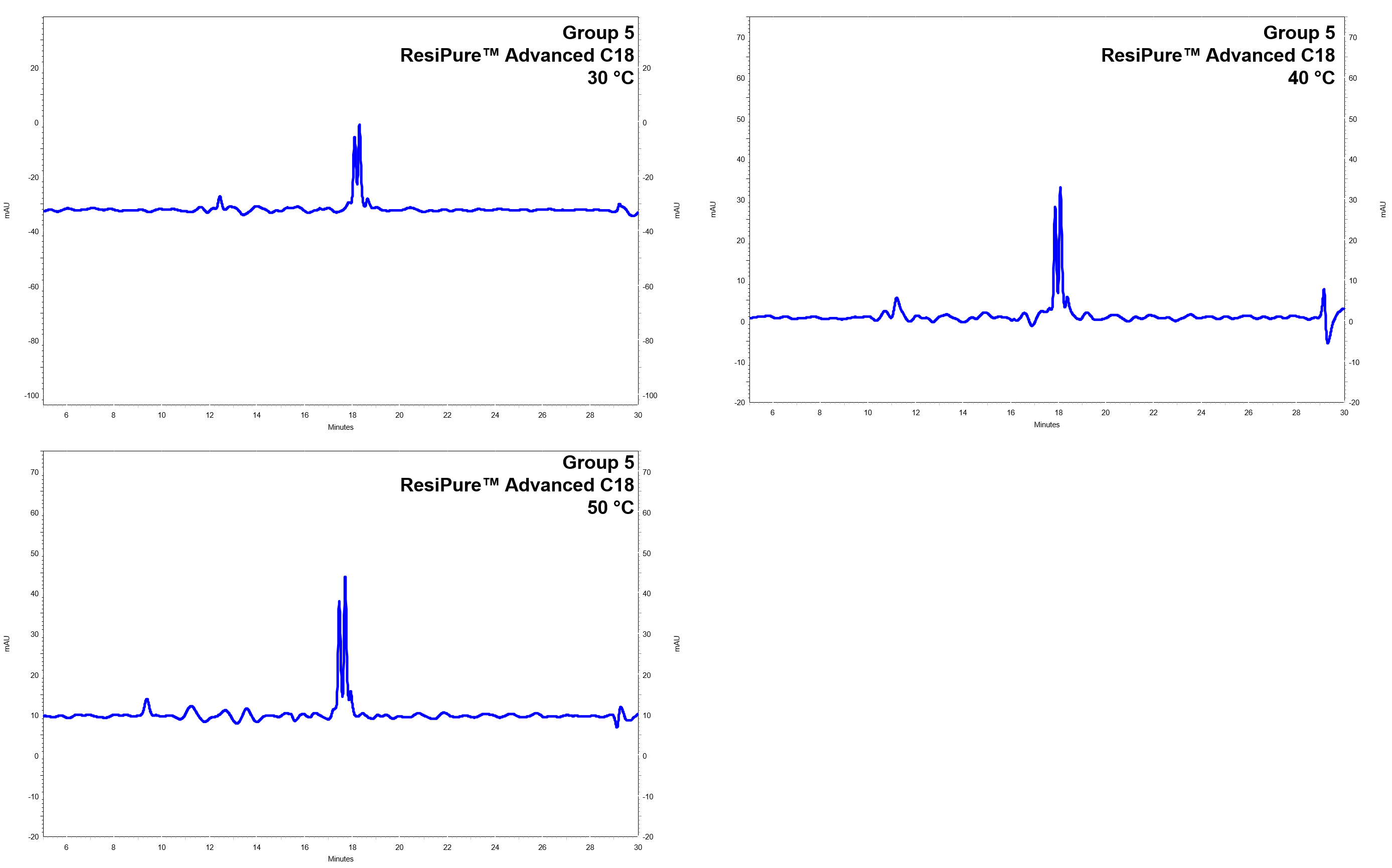


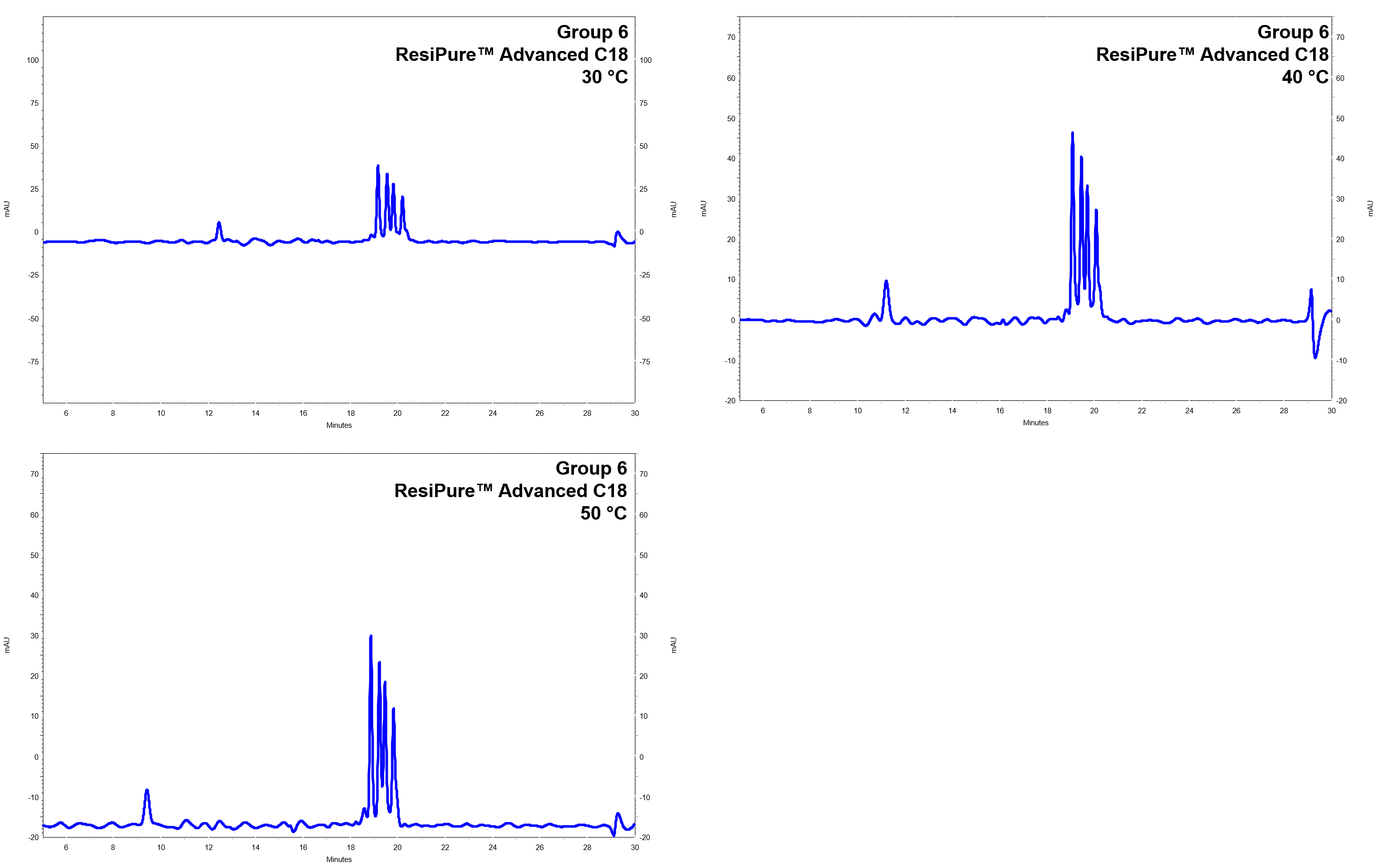


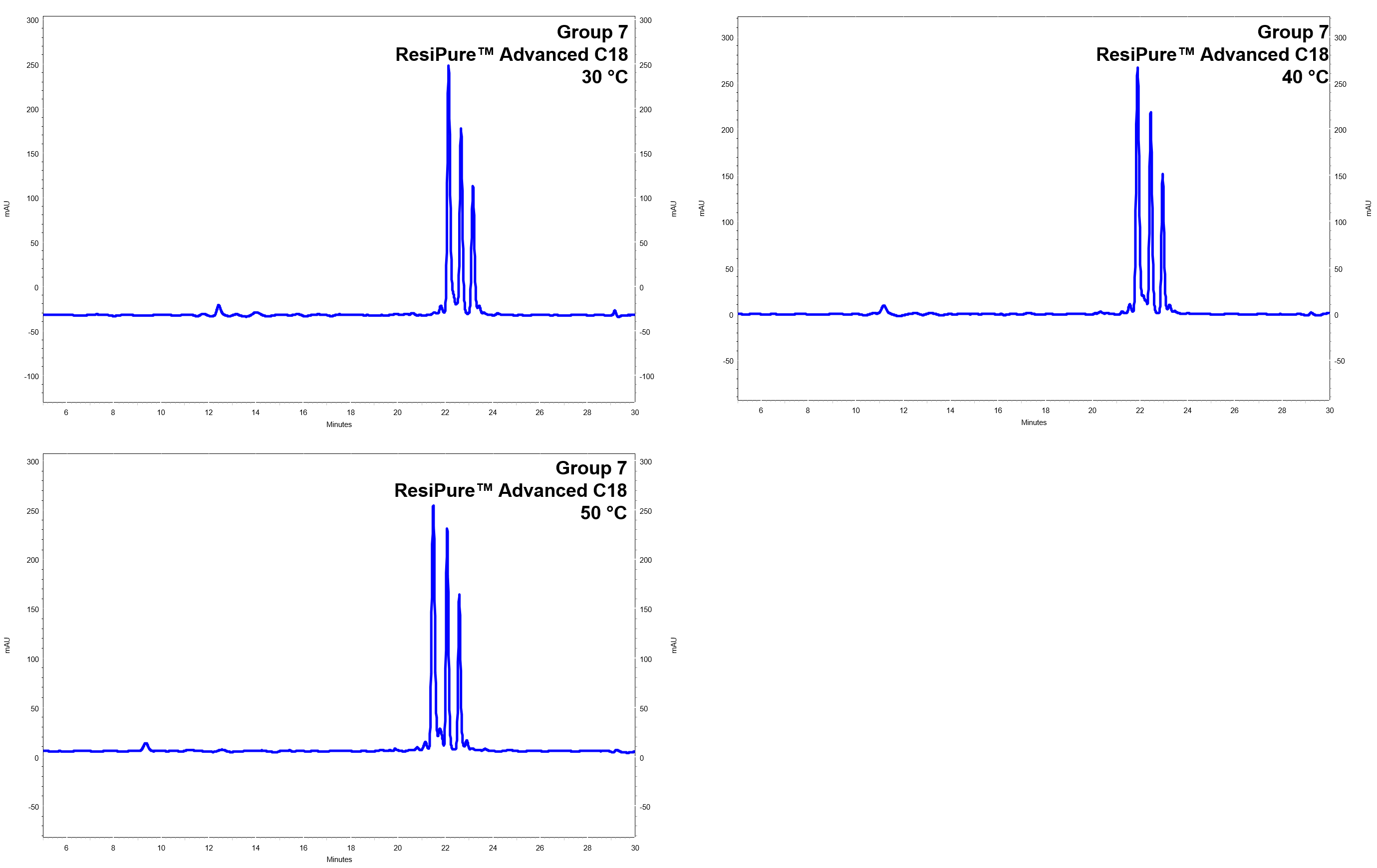


Figure SI-5.1: RP-HPLC-UV chromatograms of groups 1-7 with varying temperatures measured on the ResiPure™ Advanced C18.


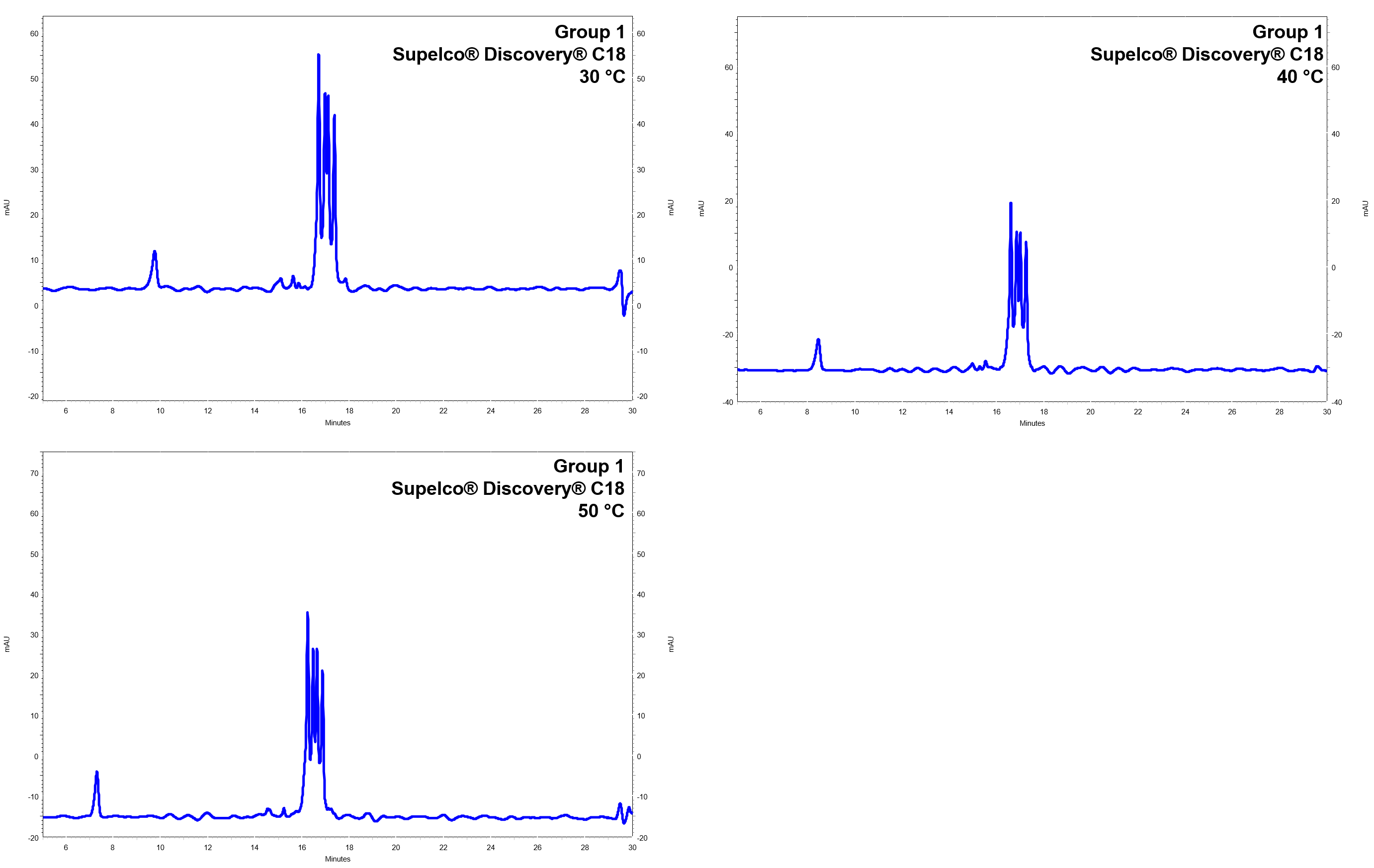


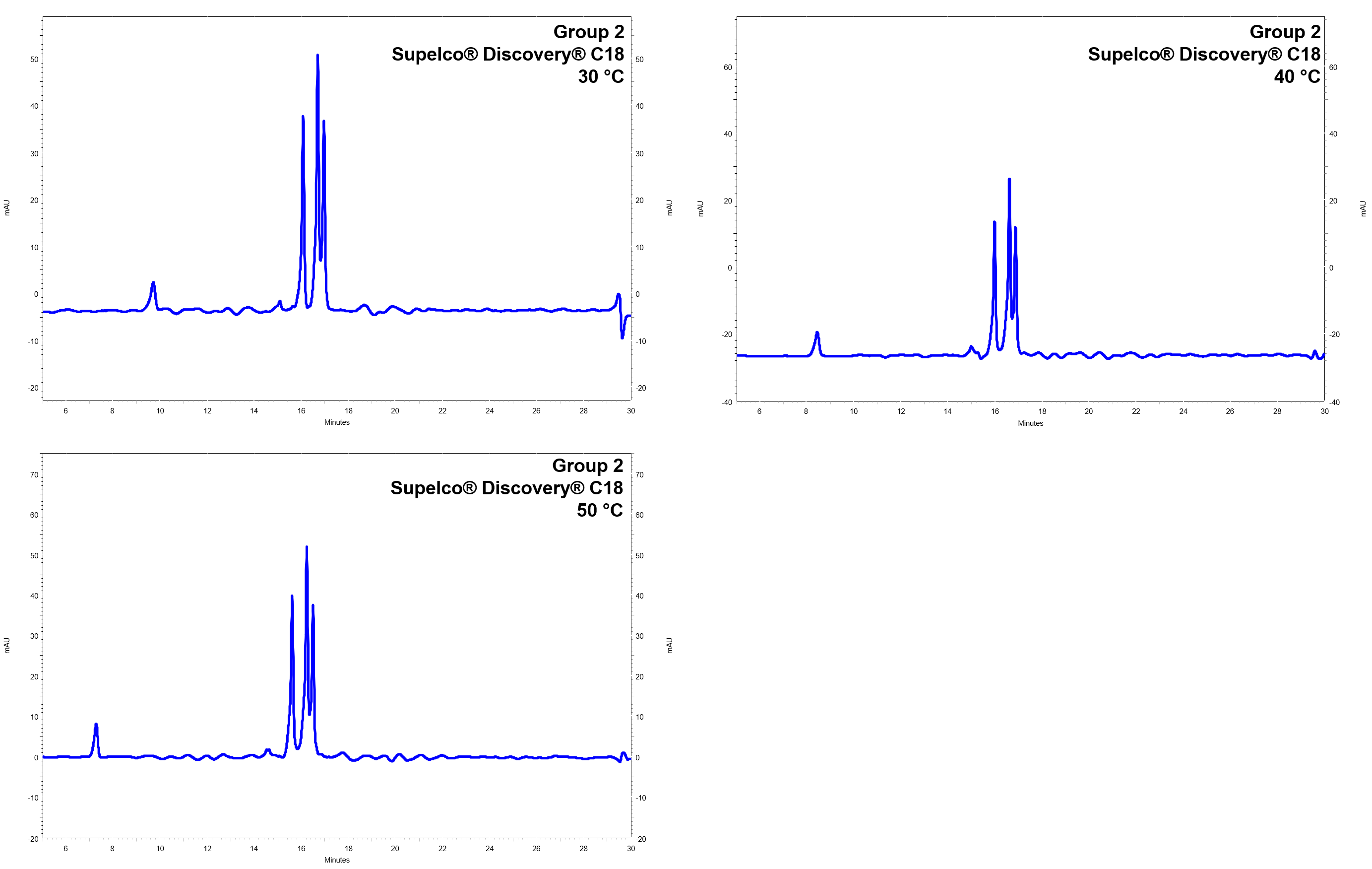


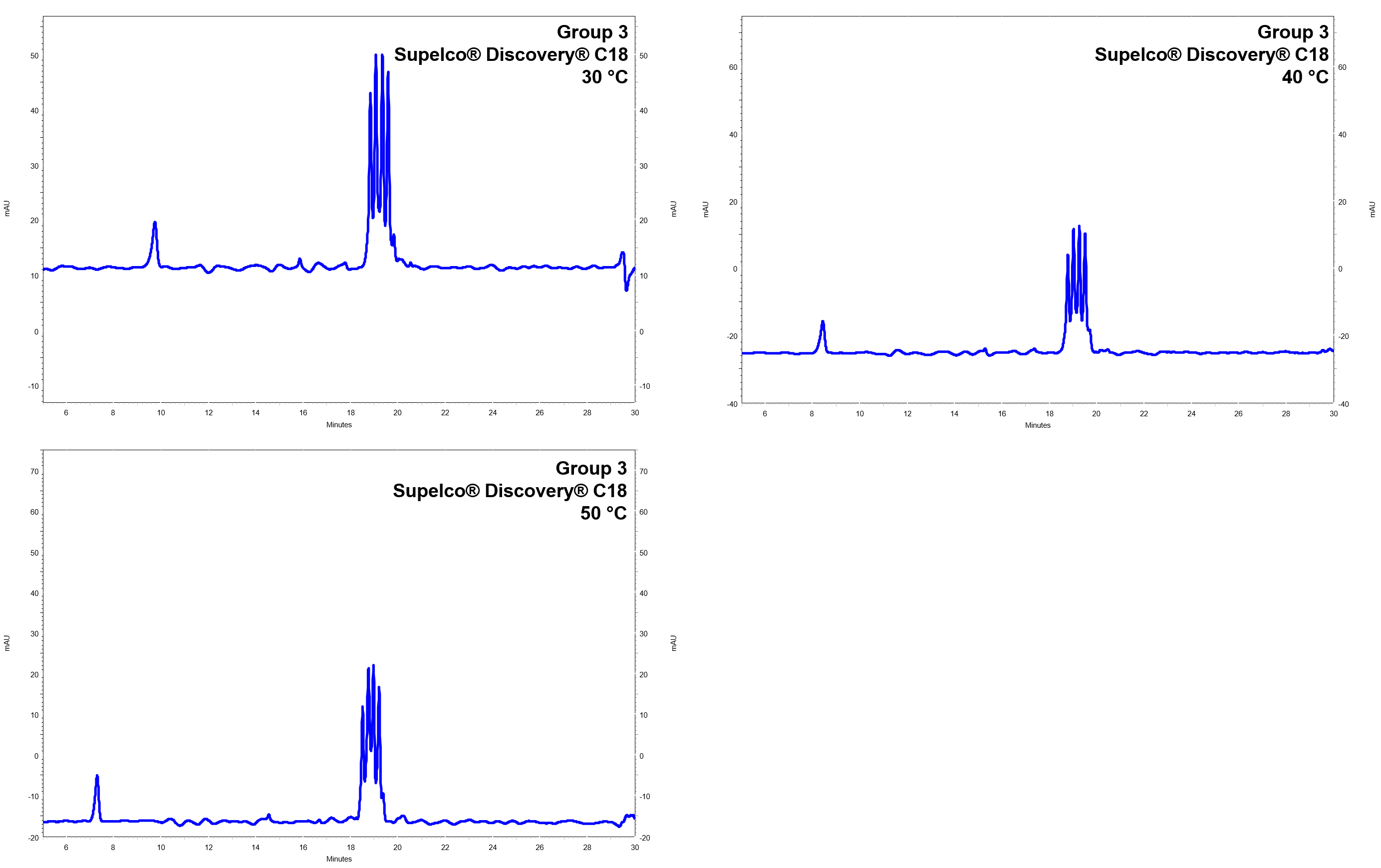


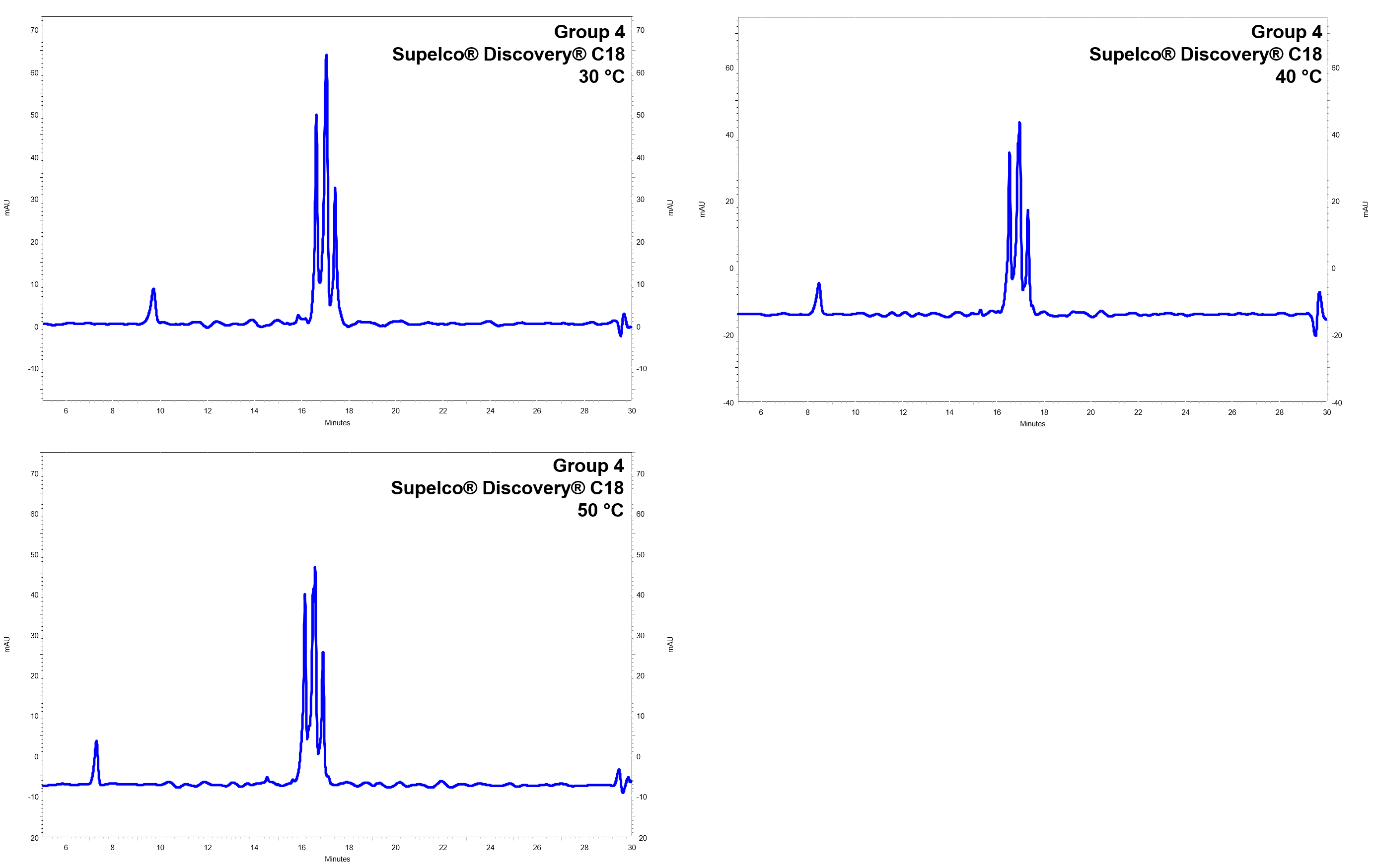


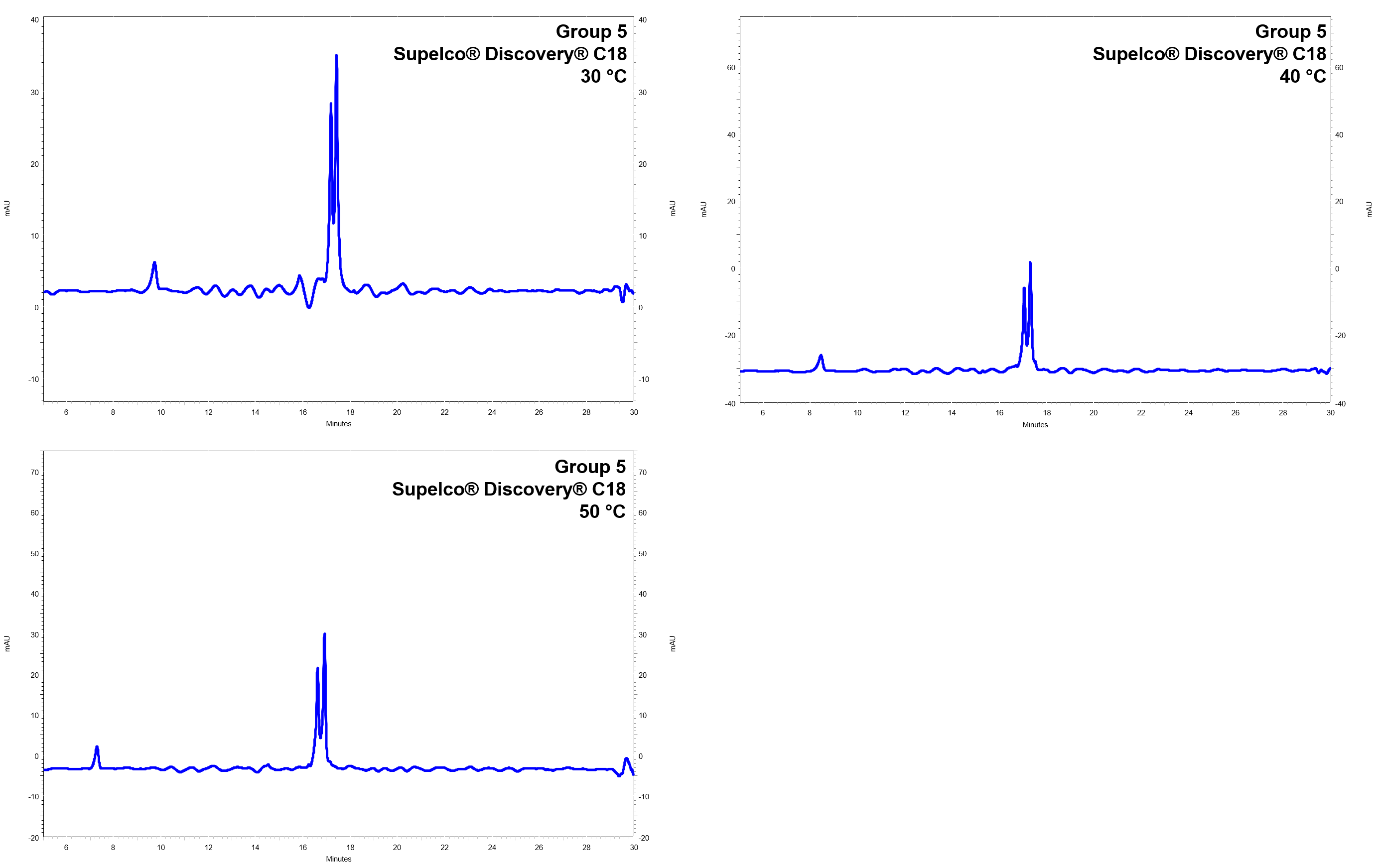


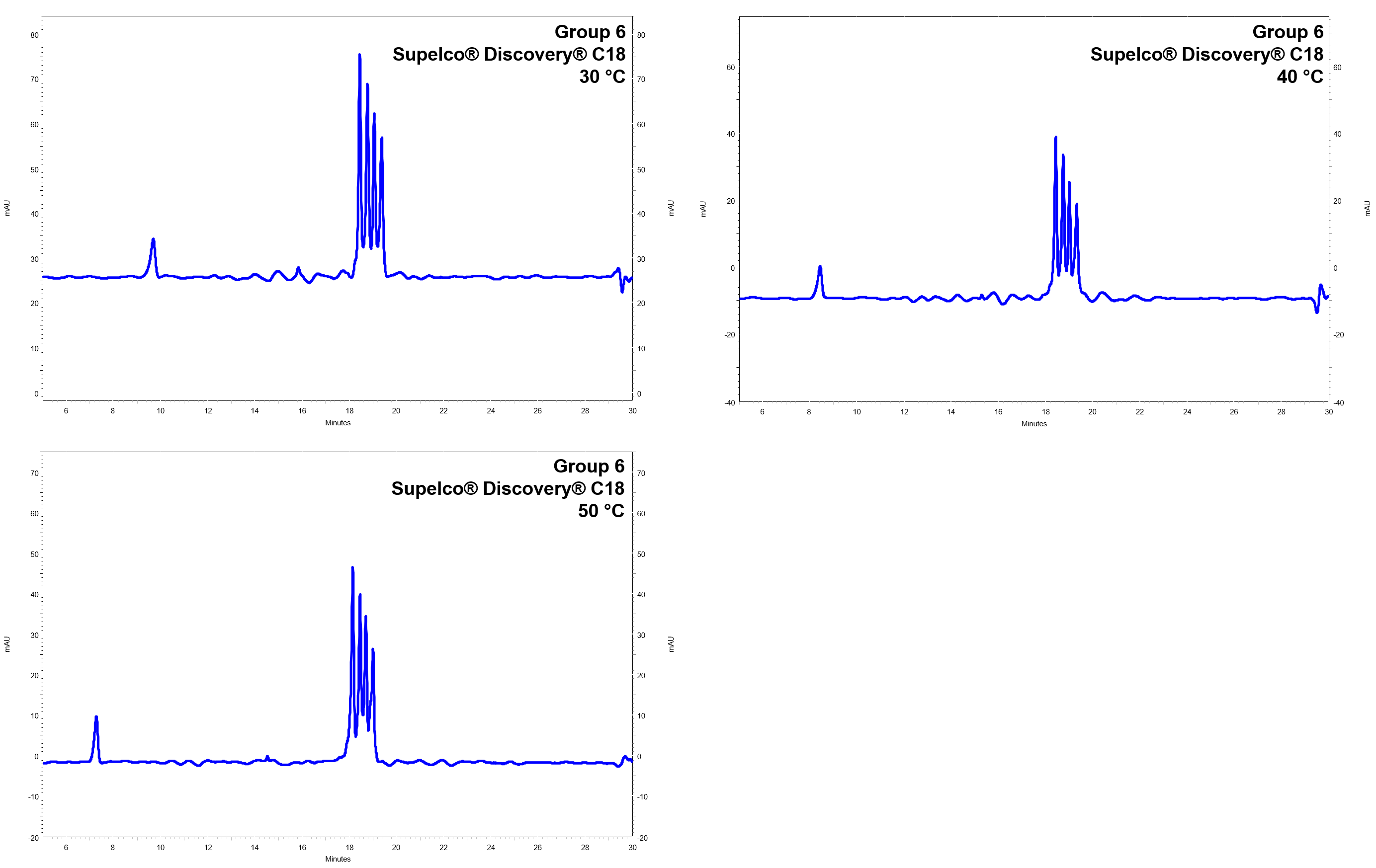


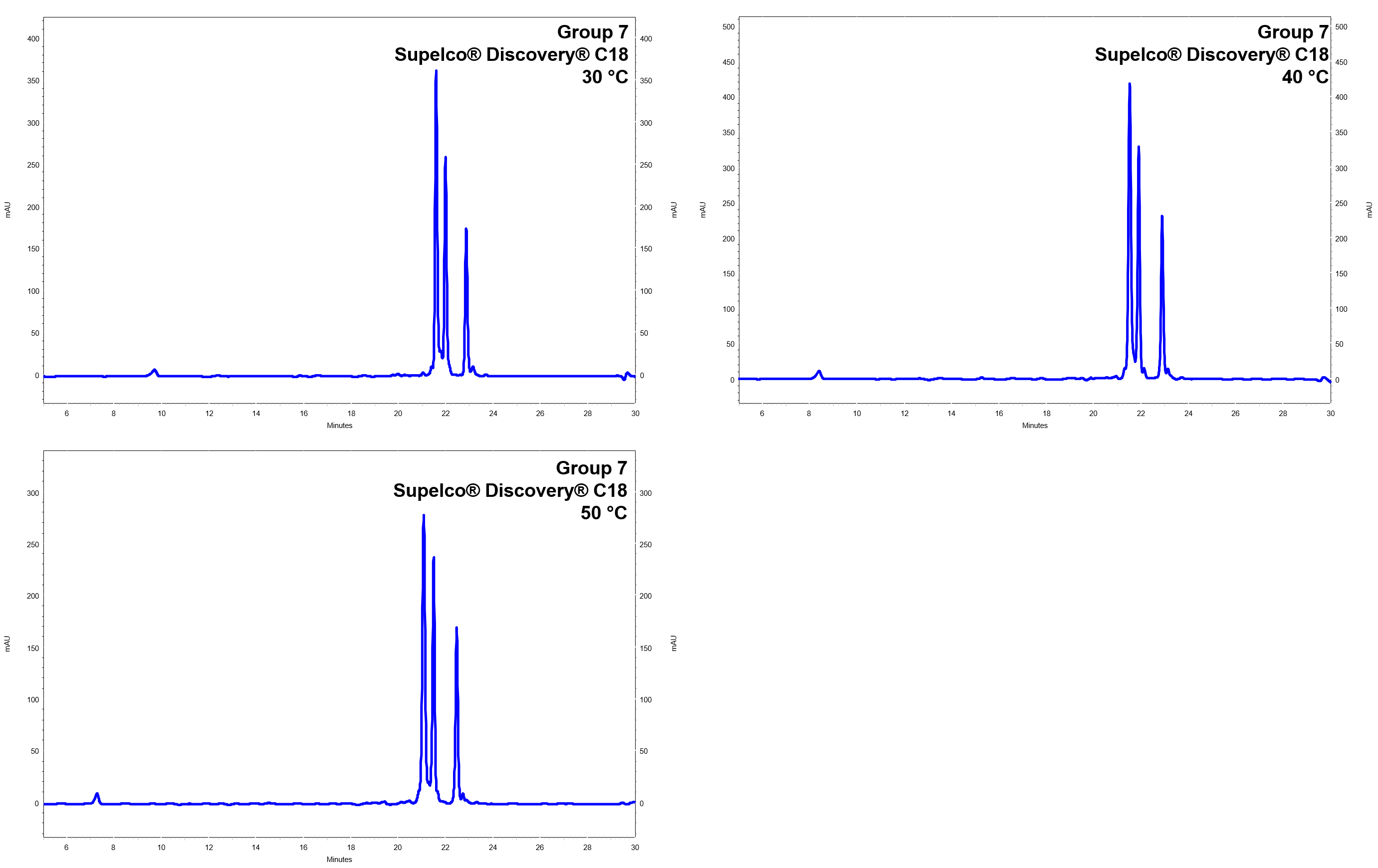


Figure SI-5.2: RP-HPLC-UV chromatograms of groups 1-7 with varying temperatures measured on the Supelco® Discovery® C18.


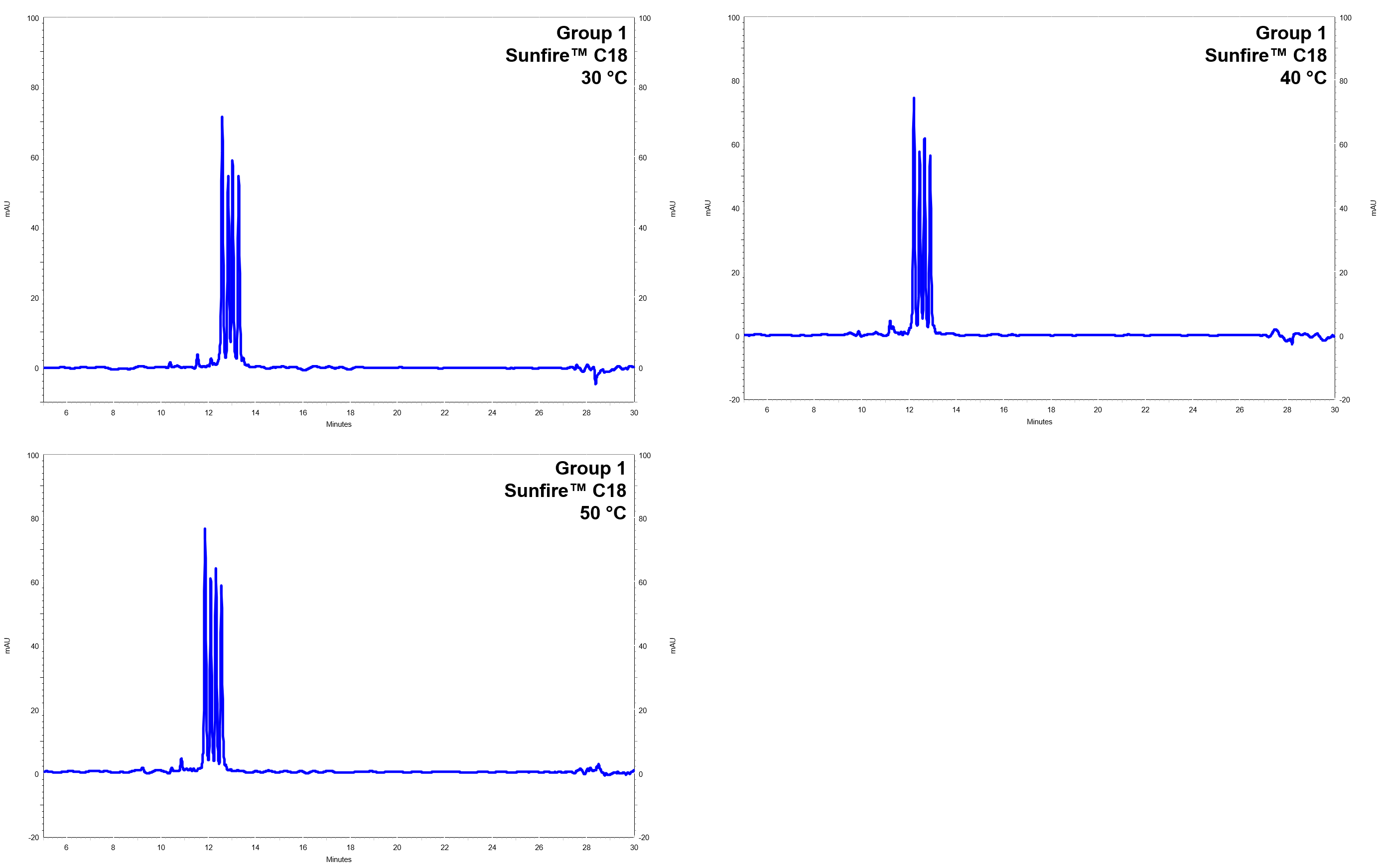


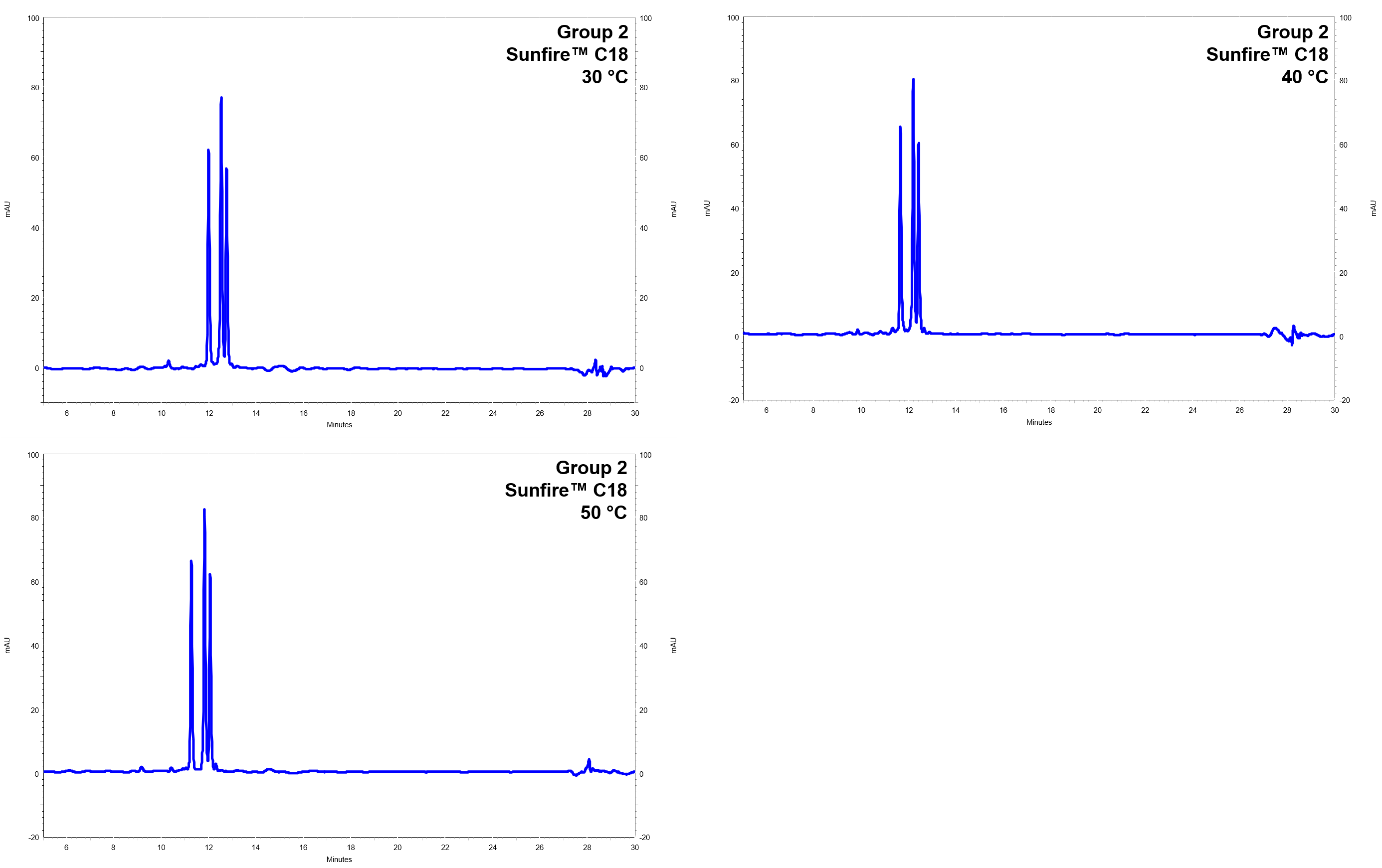


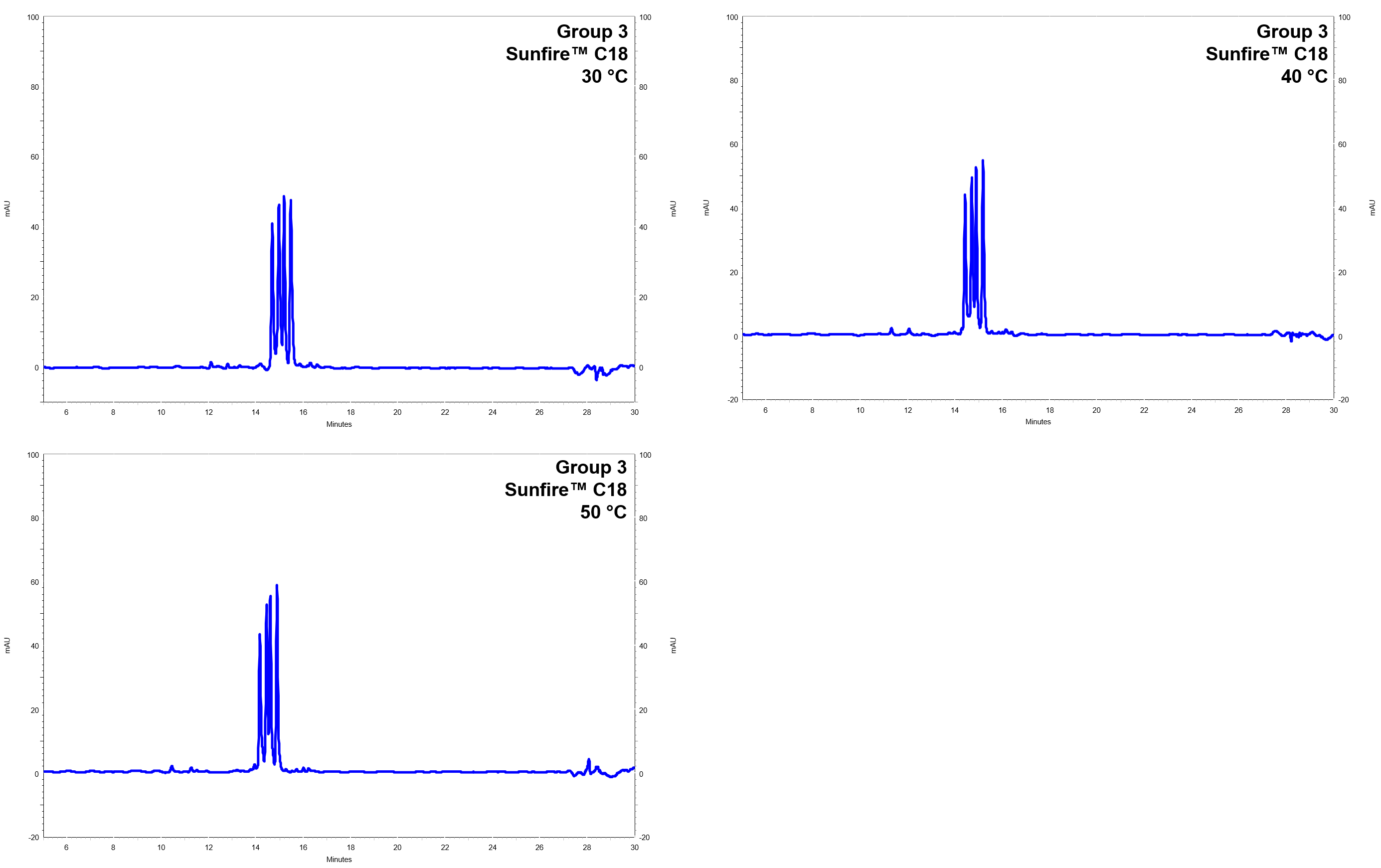


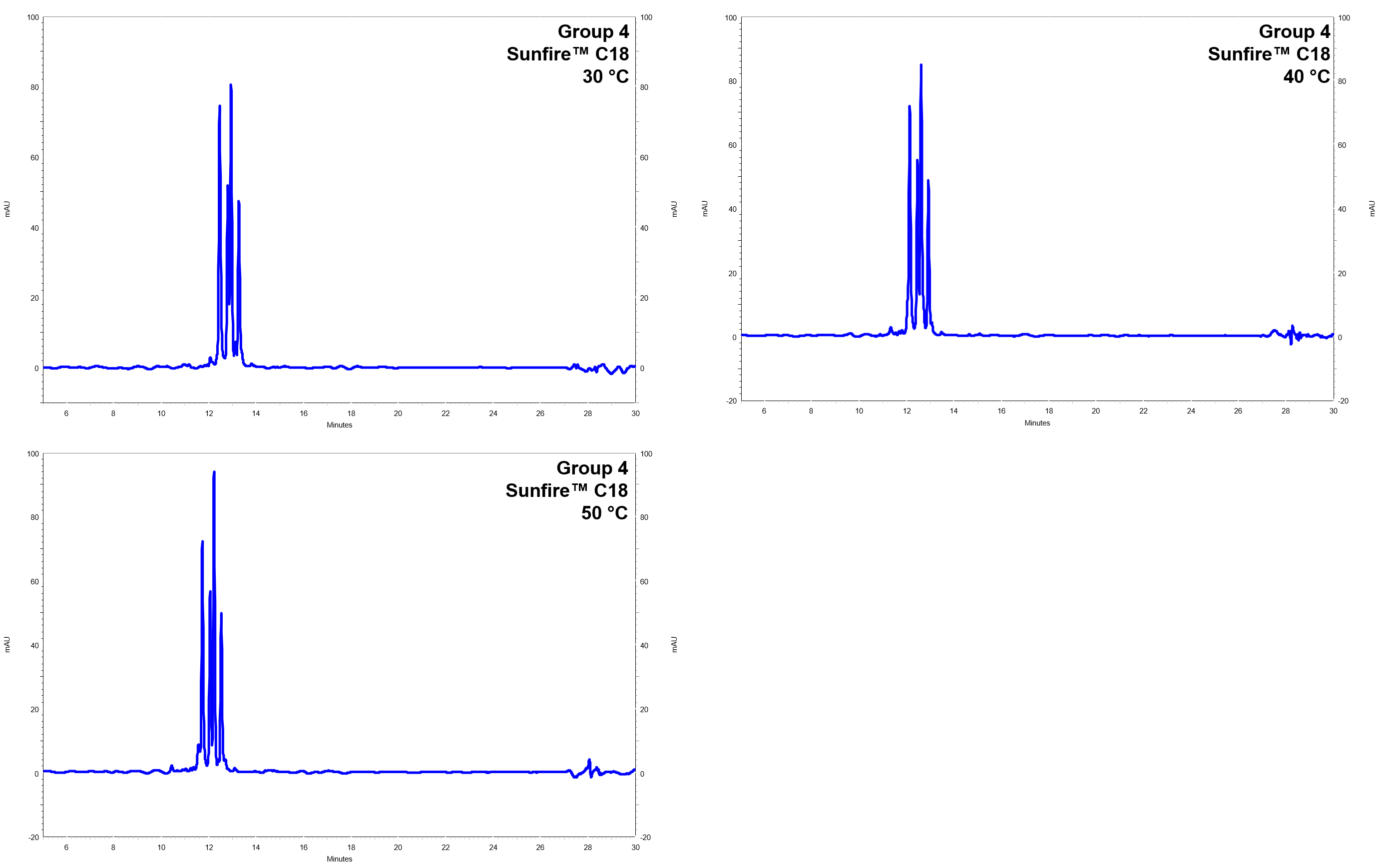


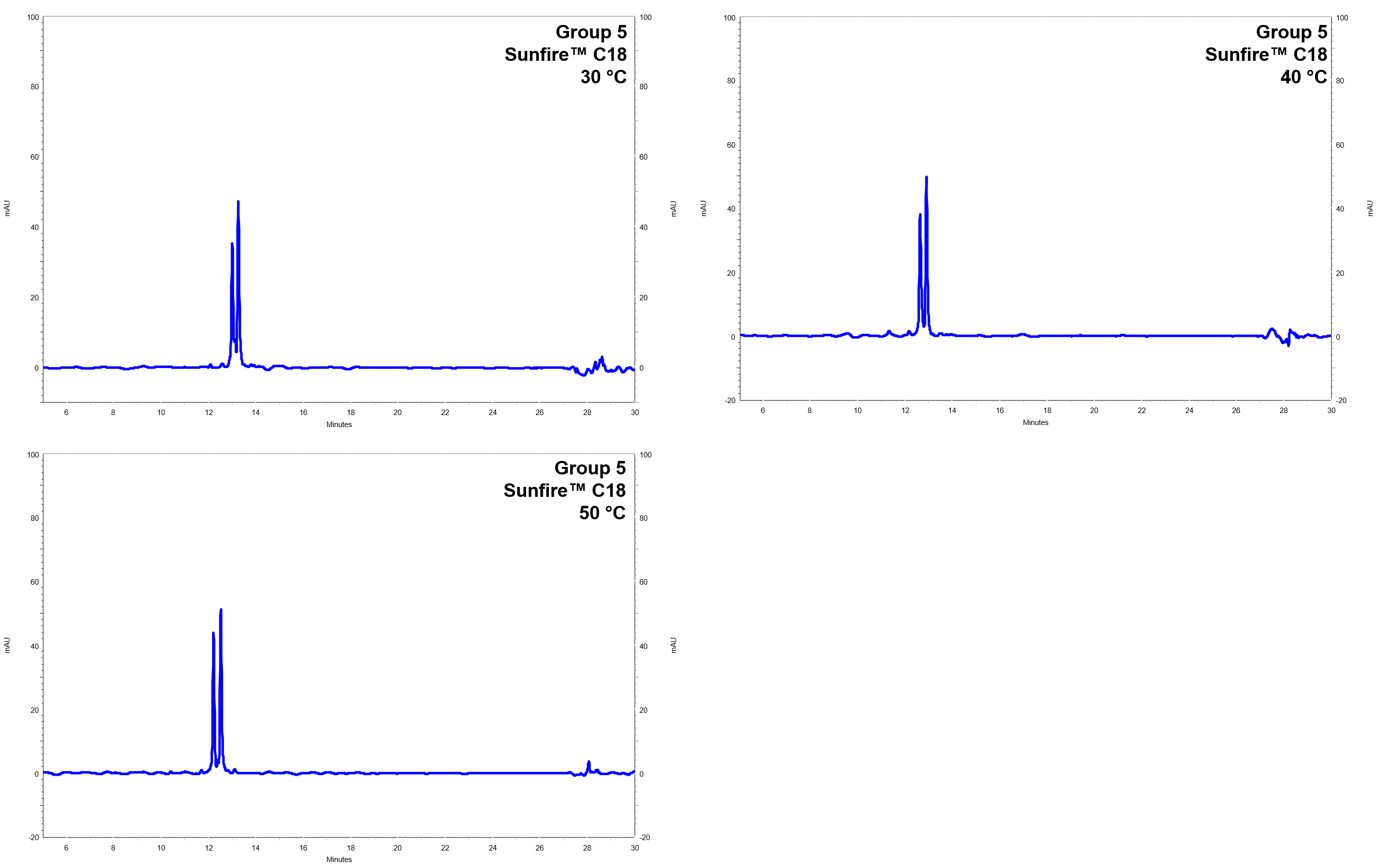


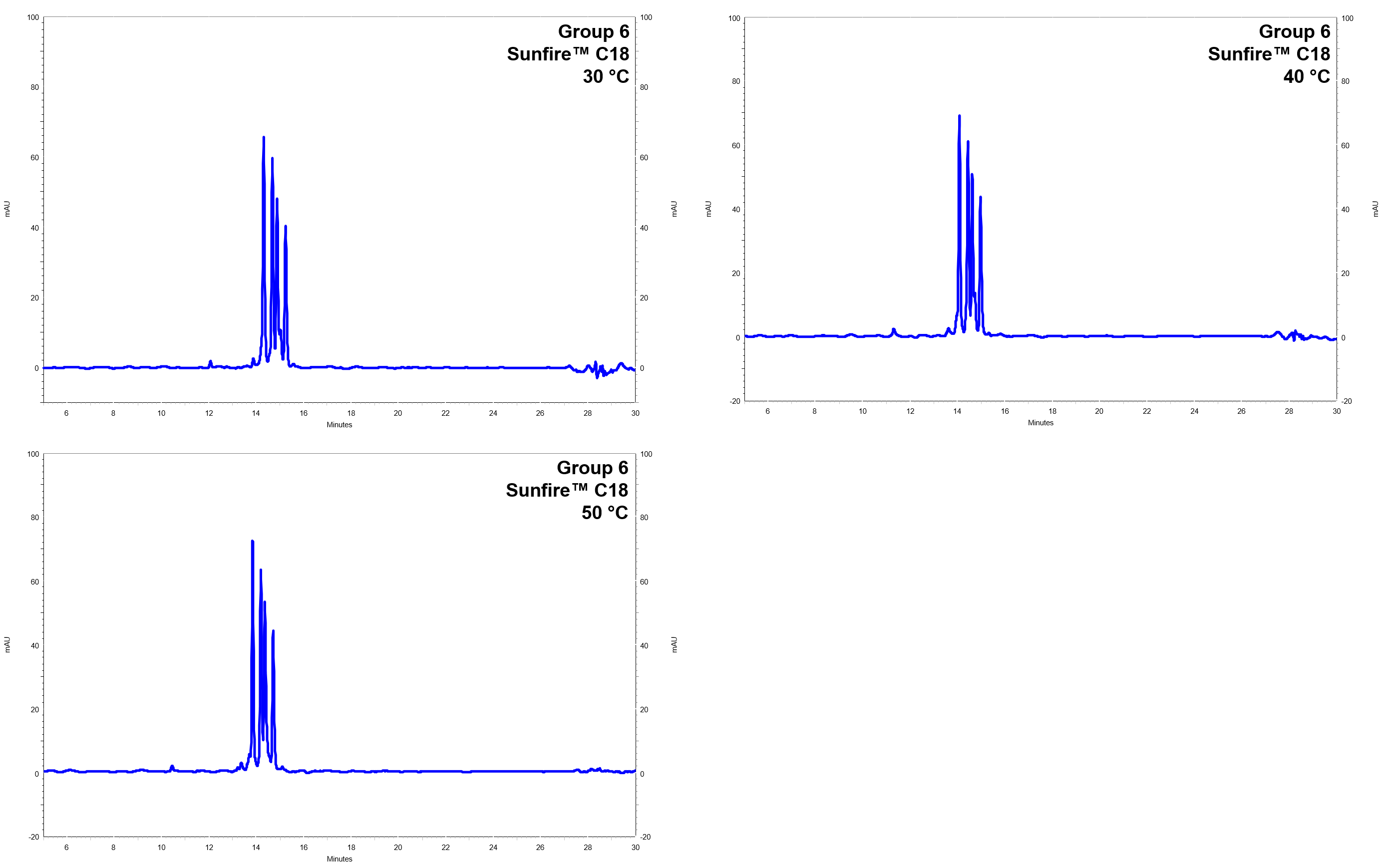


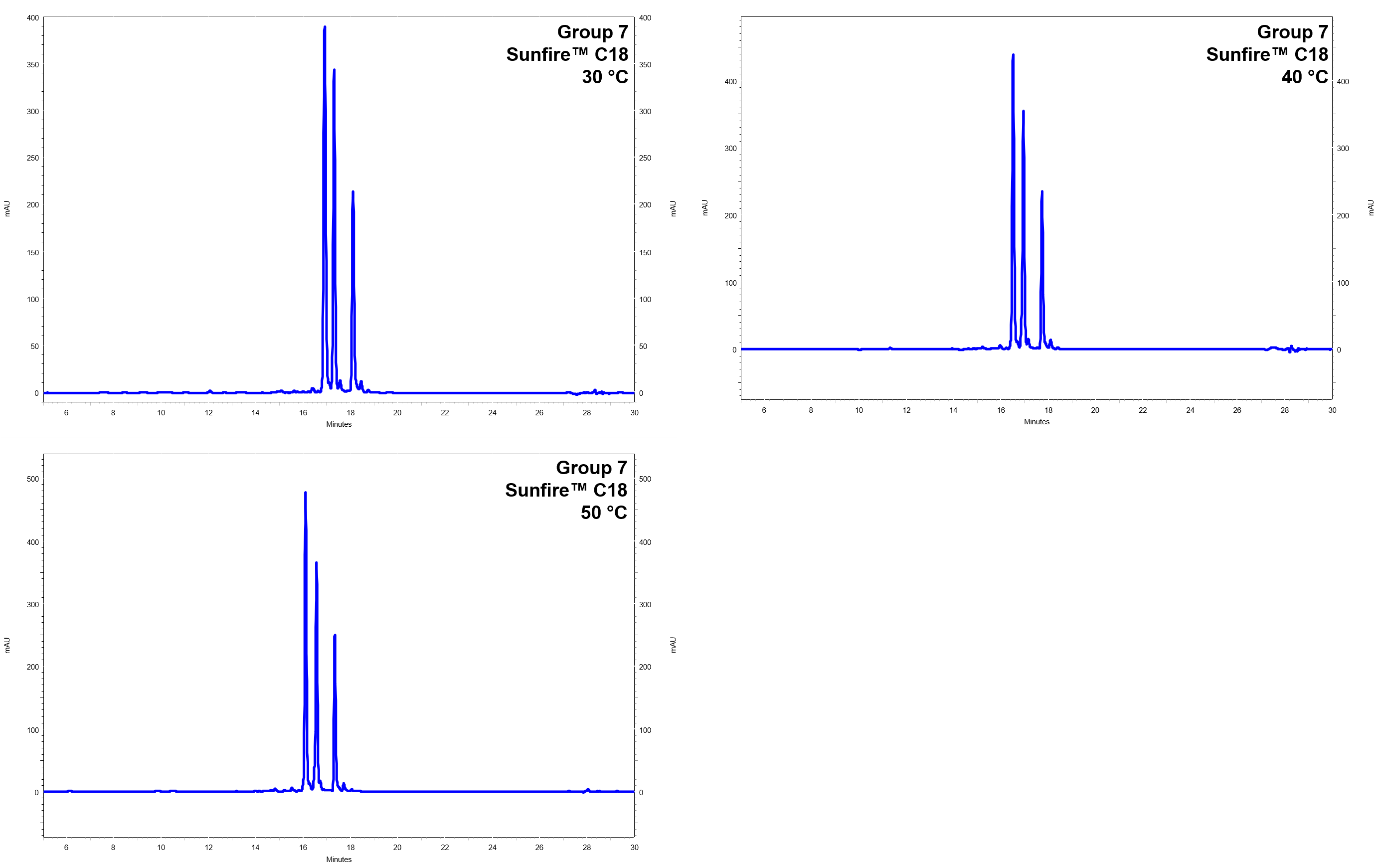


Figure SI-5.3: RP-HPLC-UV chromatograms of groups 1-7 with varying temperatures measured on the Sunfire™ C18.

# SI-6: Individual chromatograms to test the effect of the modifier on the separation efficacy


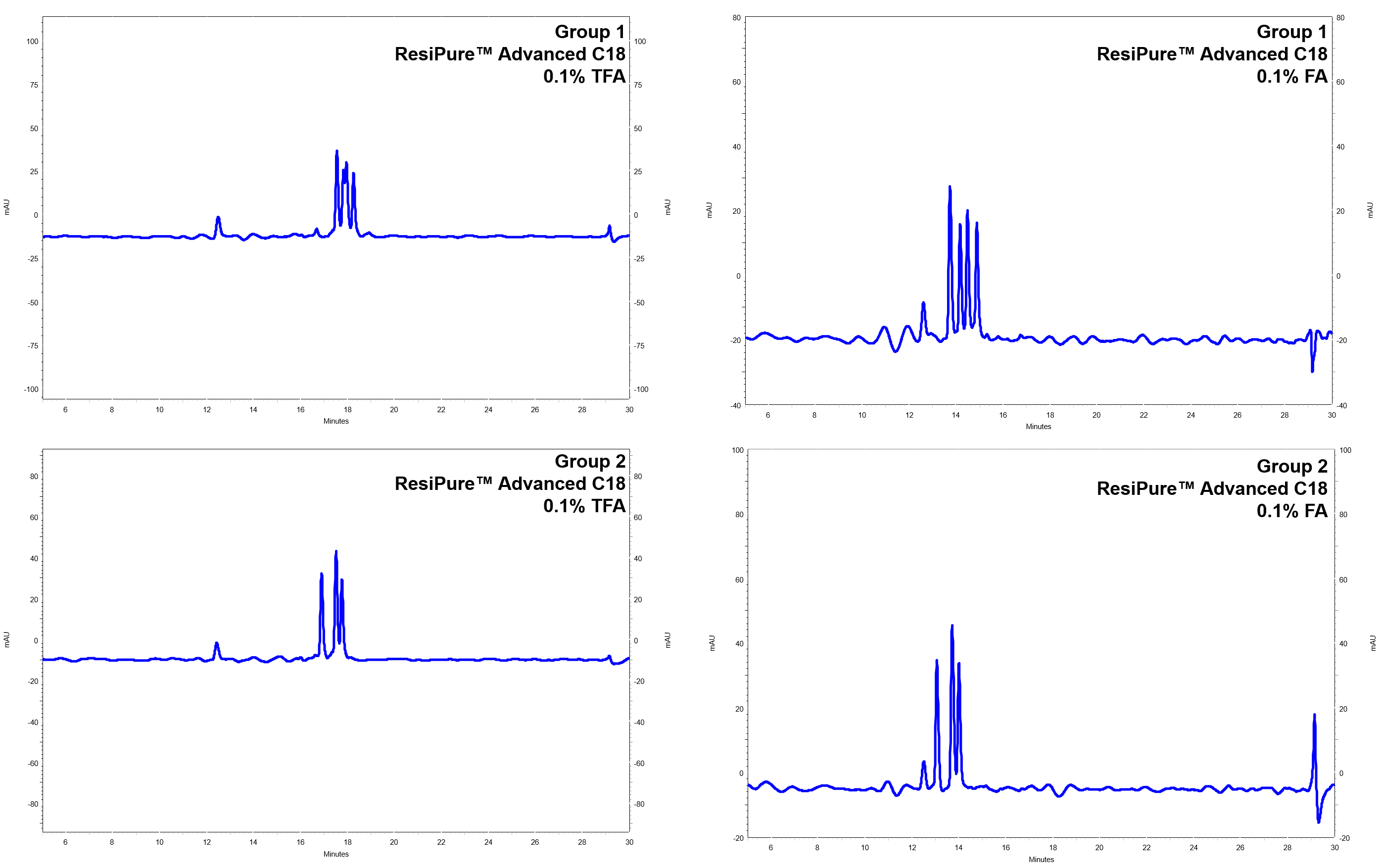


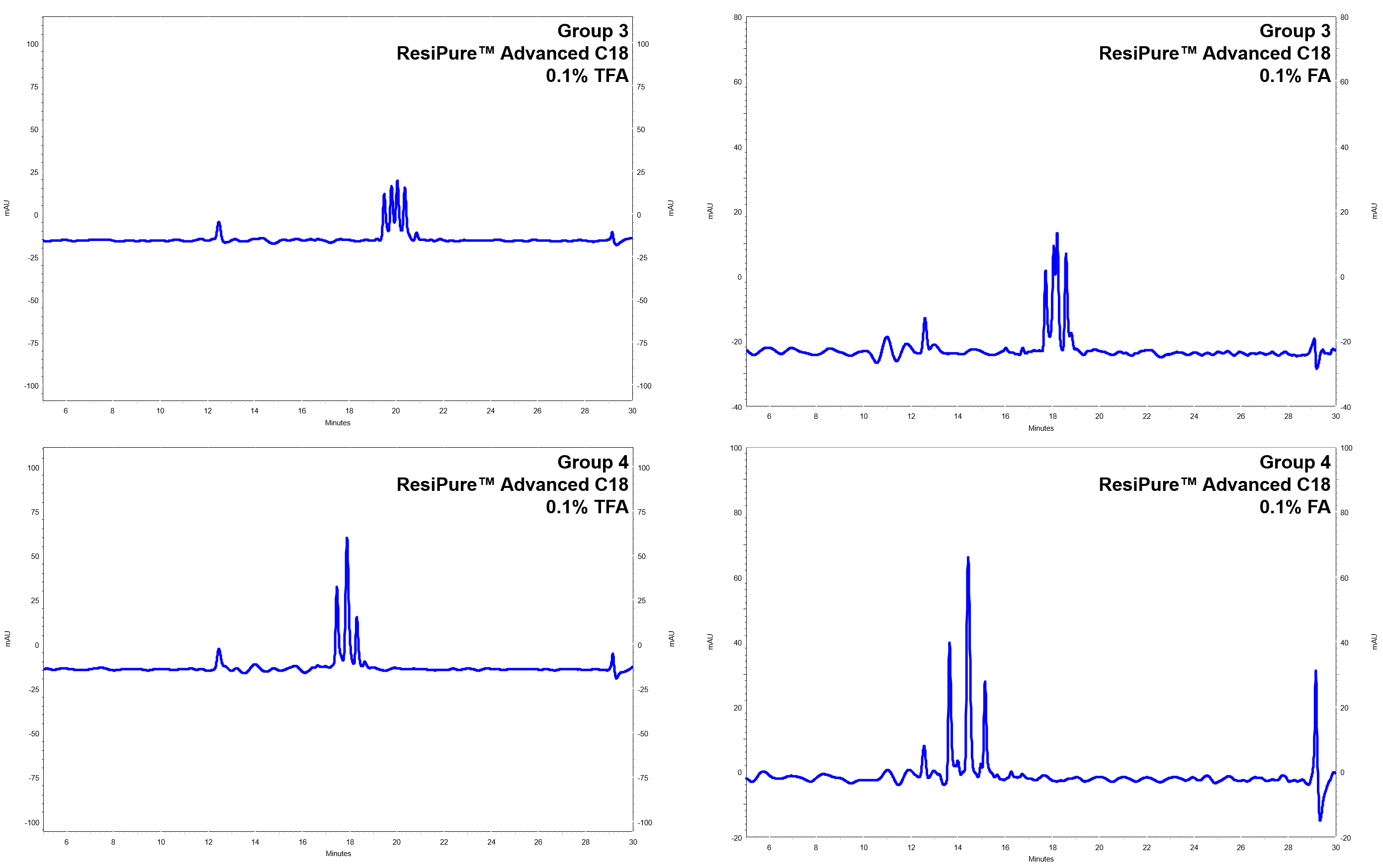


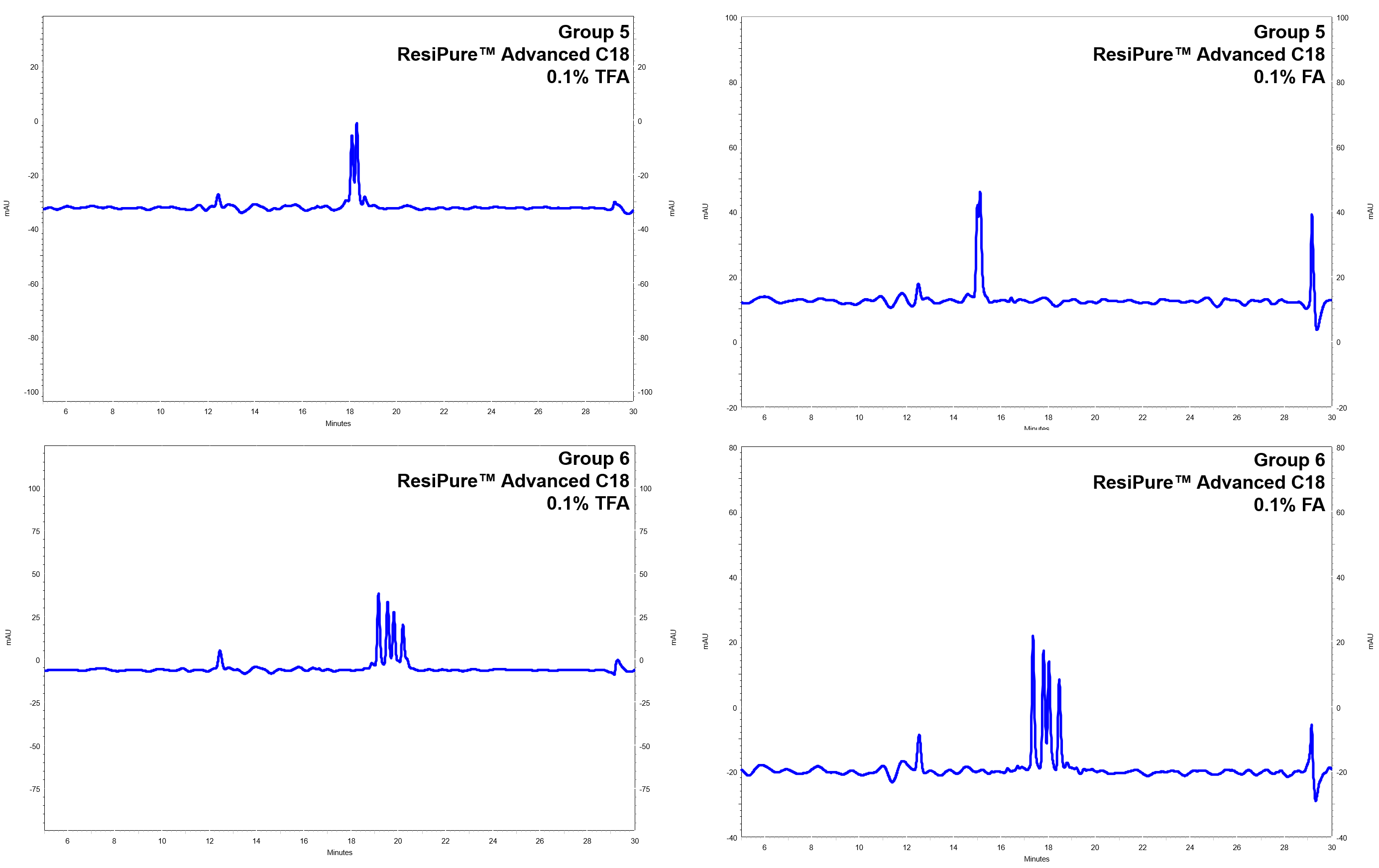


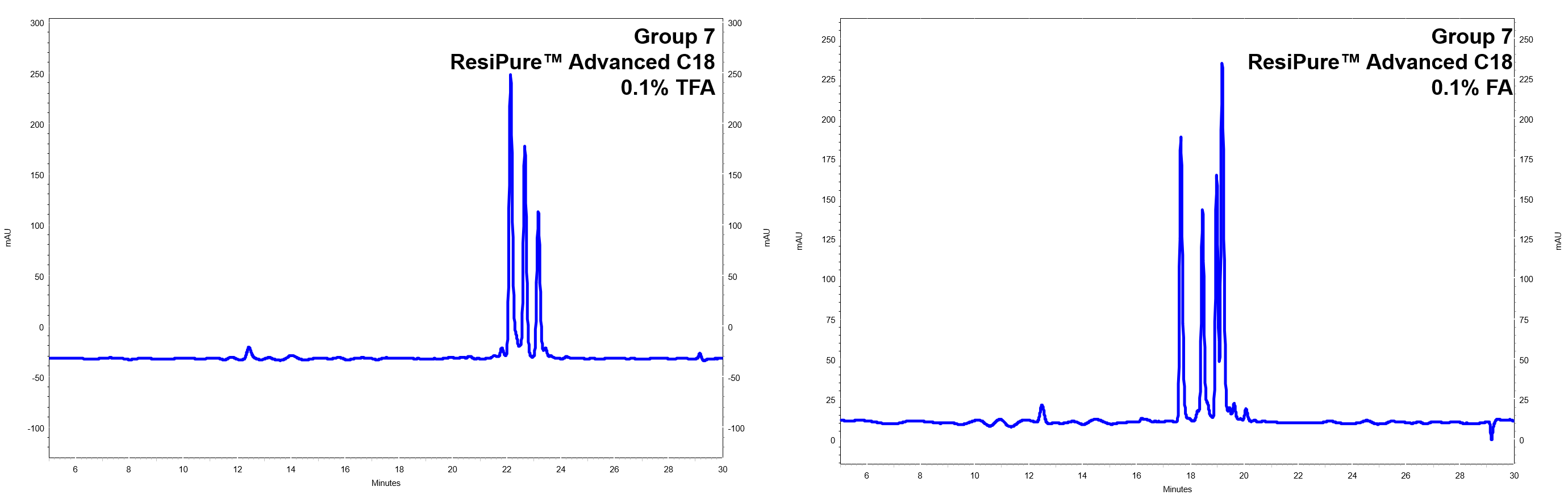


Figure SI-6.1: RP-HPLC-UV chromatograms of groups 1-7 with TFA and FA as modifiers measured on the ResiPure™ Advanced C18.


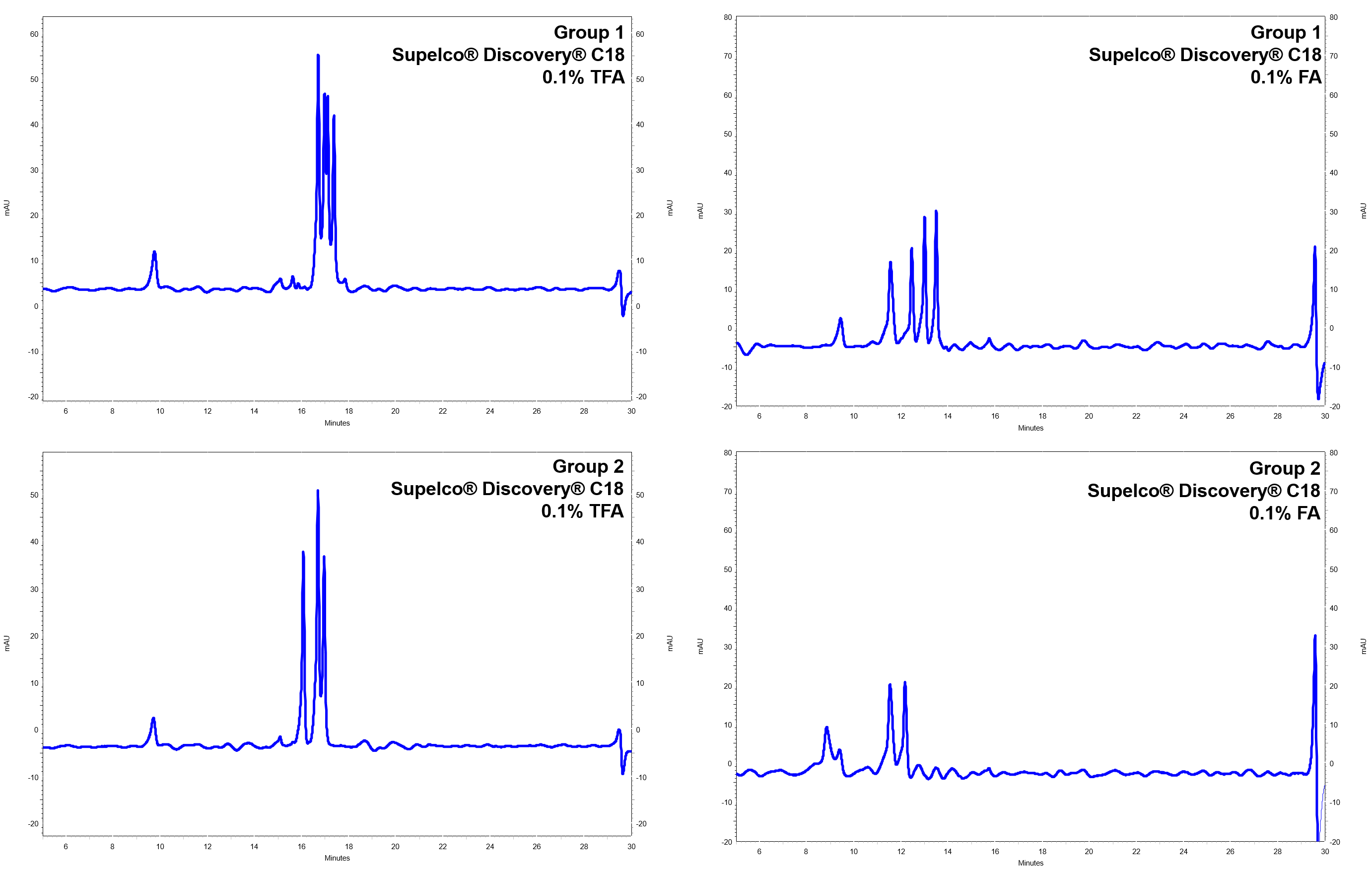


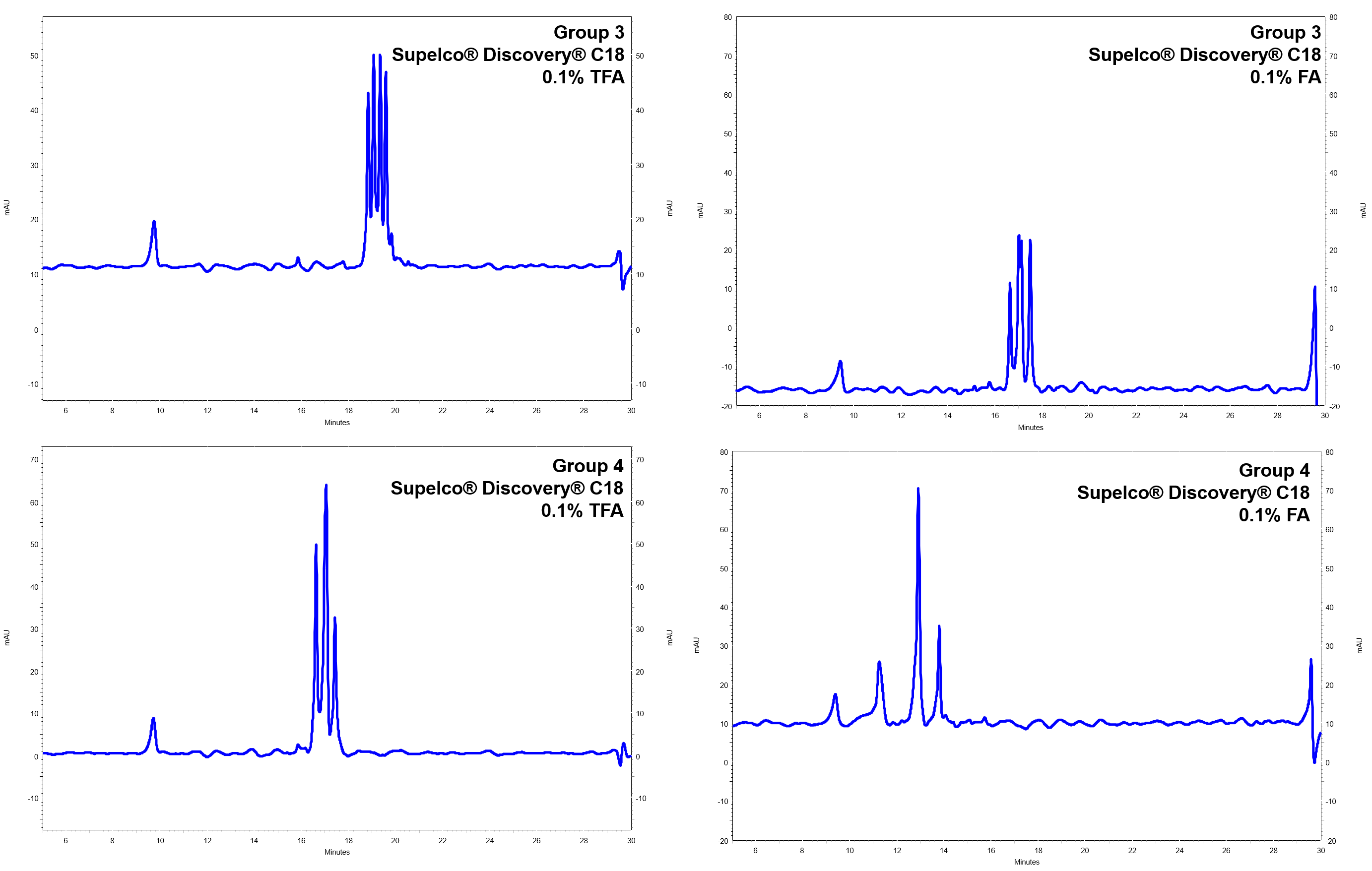


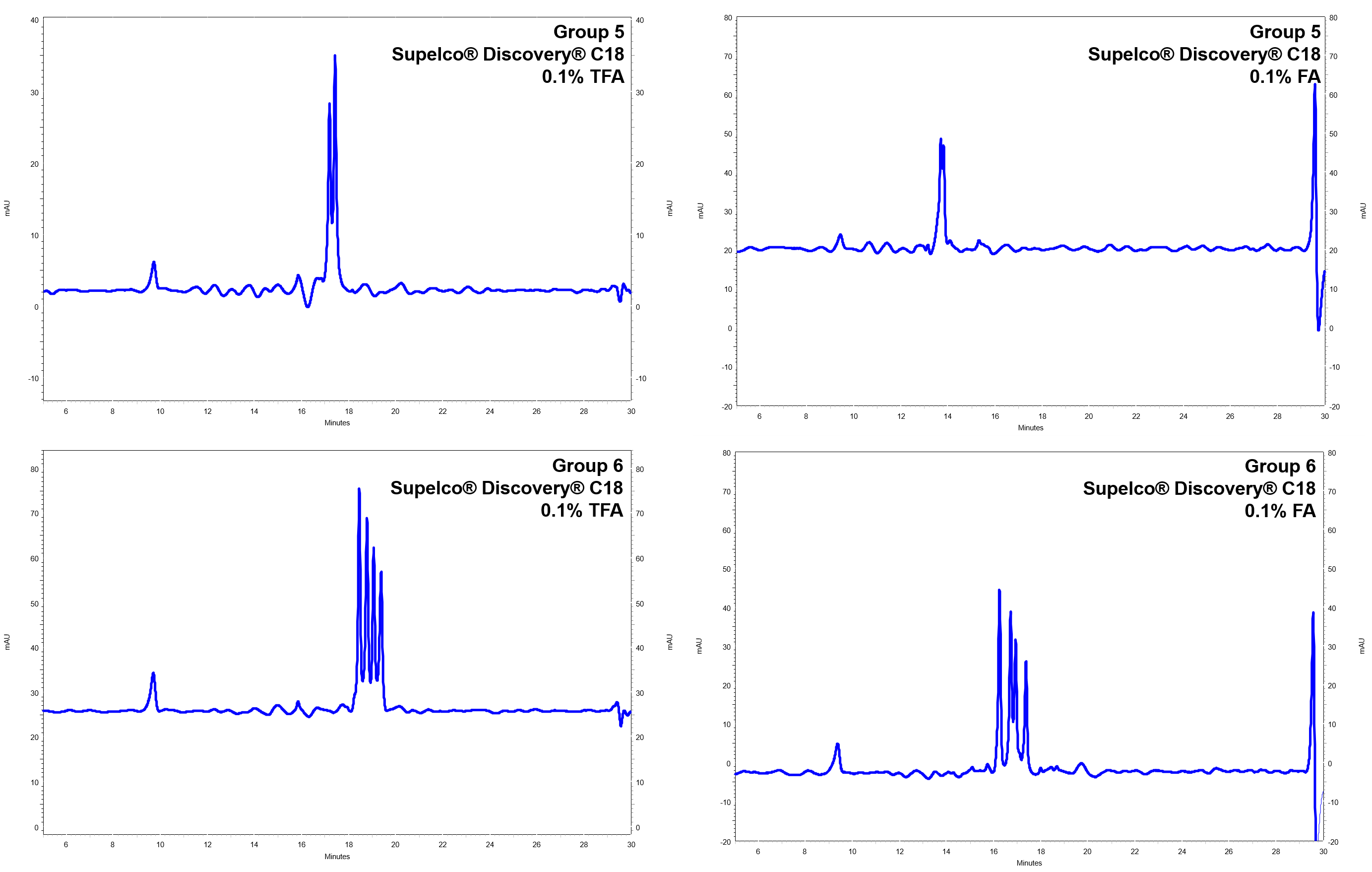


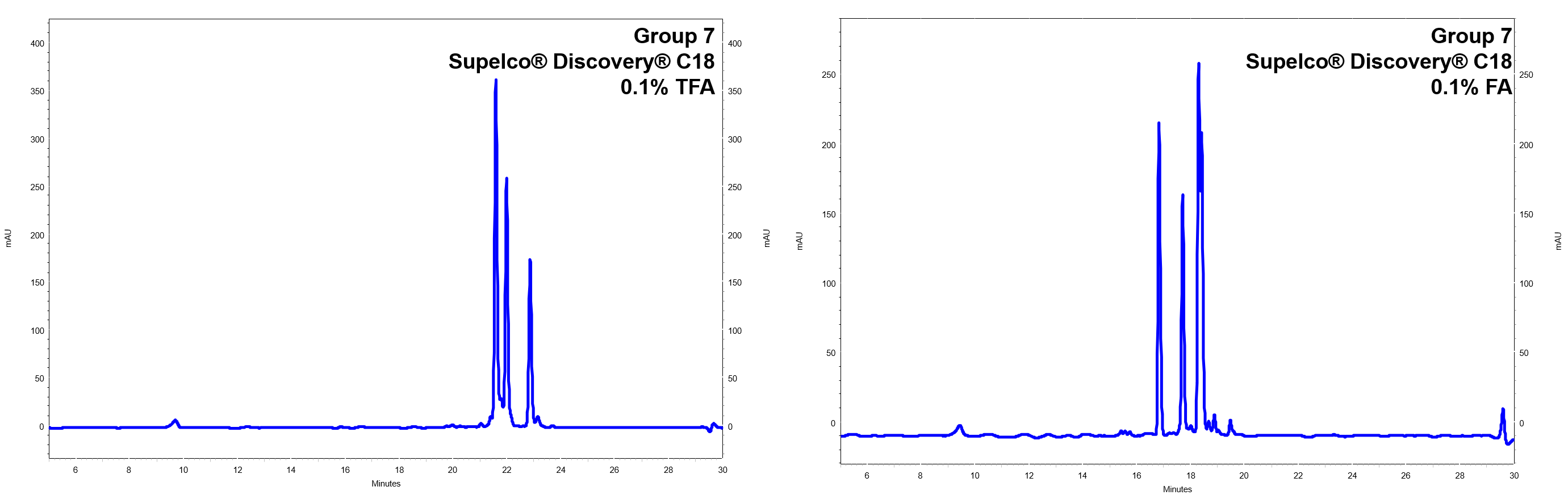


Figure SI-6.2: RP-HPLC-UV chromatograms of groups 1-7 with TFA and FA as modifiers measured on the Supelco® Discovery® C18.


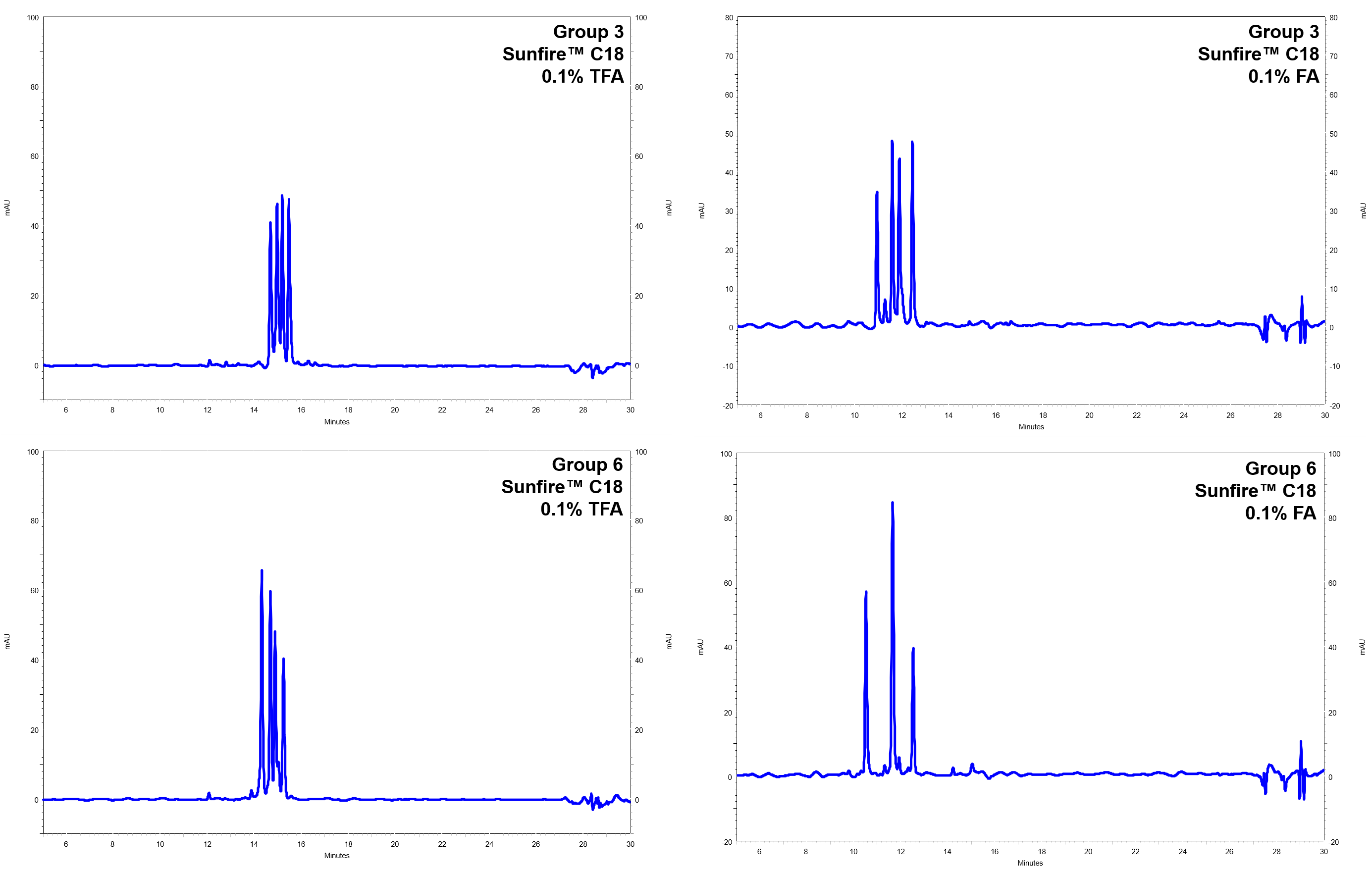


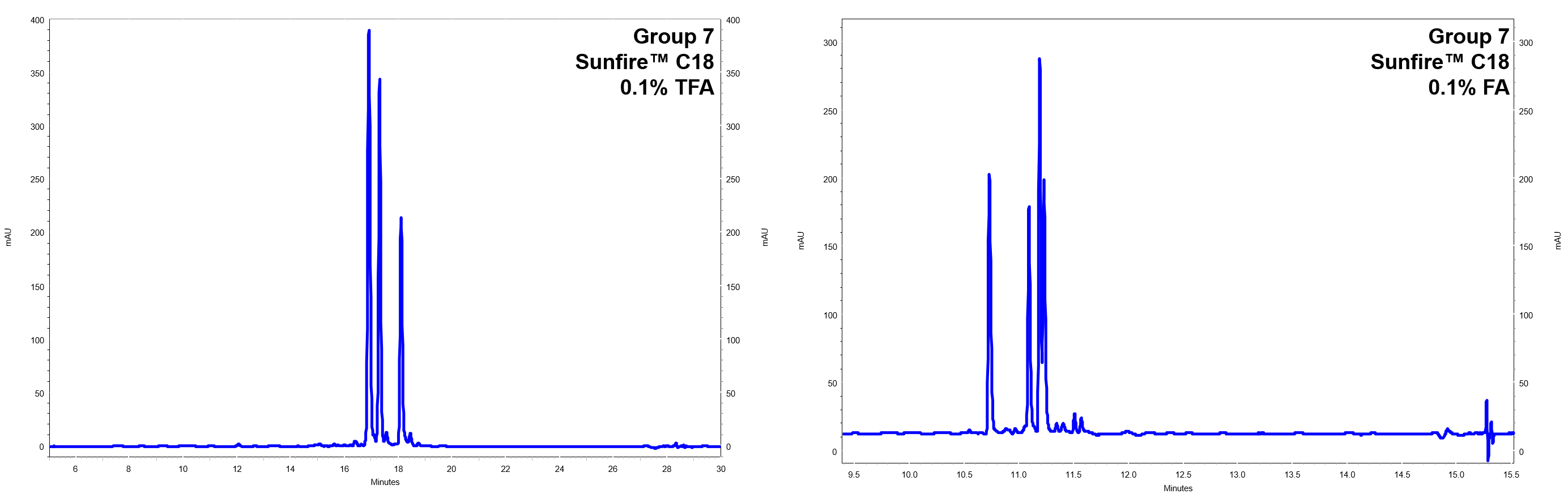


Figure SI-6.3: RP-HPLC-UV chromatograms of groups 1-7 with TFA and FA as modifiers measured on the Sunfire™ C18.

# SI-7: Improving transferability from HPLC to FPLC: Sunfire™ C18 and Discovery® C18

Table SI-7.1: Comparison of predicted, calculated, and experimentally determined ACN elution percentages for peptides P8, P9, P10, P17, P18 and P19 during HPLC-to-FPLC method transfer for the Supelco® Discovery® C18. Retention times were measured on the Supelco® Discovery® C18 at 1.0, 1.5, and 2.0 mL/min. “Pred.” refers to the direct transfer based solely on analytical elution percentages, and “Calc.” refers to the corrected values obtained using Formula 1. “Det.” indicates the experimentally observed FPLC elution percentage. ΔACN values denote the deviation between predicted or calculated percentages and the observed ACN at elution. Deviations for the direct transfer (Δ ACN Pred. [%]) is indicated in orange, deviations for the transfer with calculated values are indicated in green (Δ ACN Calc. [%]). The data illustrate the reduction of systematic transfer error achieved through application of the correction model.

| **Flash transfer from HPLC data of Supelco® Discovery® C18 (4.6 × 250 mm, 5 µm)** | | | | | | | | | | | | | | | | |
| --- | --- | --- | --- | --- | --- | --- | --- | --- | --- | --- | --- | --- | --- | --- | --- | --- |
|  | **HPLC Rt**  **[min]** | | | **FPLC ACN Perc. Det. [%]** | **FPLC ACN Perc. Pred.**  **[%]** | | | **Δ ACN Pred.**  **[%]** | | | **FPLC ACN Perc. Calc.**  **[%]** | | | **Δ ACN Calc.**  **[%]** | | |
| **Sample** | **1.0 mL/min** | **1.5 mL/min** | **2.0 mL/min** | **30.0**  **mL/min** | **1.0 mL/min** | **1.5 mL/min** | **2.0 mL/min** | **1.0 mL/min** | **1.5 mL/min** | **2.0 mL/min** | **1.0 mL/min** | **1.5 mL/min** | **2.0 mL/min** | **1.0 mL/min** | **1.5 mL/min** | **2.0 mL/min** |
| P8 | 19.35 | 16.59 | 15.13 | 17.0 | 33.7 | 28.2 | 25.3 | -16.7 | -11.2 | -8.3 | 21.9 | 20.3 | 19.3 | -4.9 | -3.3 | -2.3 |
| P9 | 19.07 | 16.32 | 14.87 | 19.2 | 33.1 | 27.6 | 24.7 | -13.9 | -8.4 | -5.5 | 21.3 | 19.7 | 18.8 | -2.1 | -0.5 | 0.4 |
| P10 | 19.59 | 16.82 | 15.36 | 19.2 | 34.2 | 28.6 | 25.7 | -15.0 | -9.4 | -6.5 | 22.3 | 20.7 | 19.8 | -3.1 | -1.5 | -0.6 |
| P17 | 19.07 | 16.32 | 14.89 | 17.6 | 33.1 | 27.6 | 24.8 | -15.5 | -10.0 | -7.2 | 21.3 | 19.7 | 18.9 | -3.7 | -2.1 | -1.3 |
| P18 | 18.77 | 16.05 | 14.57 | 16.3 | 32.5 | 27.1 | 24.1 | -16.2 | -10.8 | -7.8 | 20.7 | 19.2 | 18.2 | -4.4 | -2.9 | -1.9 |
| P19 | 19.38 | 16.63 | 15.15 | 19.1 | 33.8 | 28.3 | 25.3 | -14.7 | -9.2 | -6.2 | 21.9 | 20.4 | 19.4 | -2.8 | -1.3 | -0.3 |

Table SI-7.2: Comparison of predicted, calculated, and experimentally determined ACN elution percentages for peptides P8, P9, P10, P17, P18 and P19 during HPLC-to-FPLC method transfer for the Supelco® Discovery® C18. Retention times were measured on the Sunfire™ C18 at 1.0 mL/min. “Pred.” refers to the direct transfer based solely on analytical elution percentages, and “Calc.” refers to the corrected values obtained using Formula 1. “Det.” indicates the experimentally observed FPLC elution percentage. ΔACN values denote the deviation between predicted or calculated percentages and the observed ACN at elution. Deviations for the direct transfer (Δ ACN Pred. [%]) is indicated in orange, deviations for the transfer with calculated values are indicated in green (Δ ACN Calc. [%]). The data illustrate the reduction of systematic transfer error achieved through application of the correction model.

| **Flash transfer from HPLC data of Sunfire™ C18 (3.0 × 150 mm, 3.5 µm)** | | | | | | |
| --- | --- | --- | --- | --- | --- | --- |
|  | **HPLC Rt [min]** | **FPLC ACN Perc. Det. [%]** | **FPLC ACN Perc. Pred. [%]** | **Δ ACN Pred. [%]** | **FPLC ACN Perc. Calc. [%]** | **Δ ACN Calc. [%]** |
| **Sample** | **1.0 mL/min** | **30.0 mL/min** | **1.0 mL/min** | **1.0 mL/min** | **1.0 mL/min** | **1.0 mL/min** |
| P8 | 15.11 | 17.0 | 25.2 | -8.2 | 19.6 | -2.56 |
| P9 | 14.88 | 19.2 | 24.8 | -5.6 | 19.1 | 0.10 |
| P10 | 15.39 | 19.2 | 25.8 | -6.6 | 20.1 | -0.92 |
| P17 | 14.82 | 17.6 | 24.6 | -7.0 | 19.0 | -1.38 |
| P18 | 14.61 | 16.3 | 24.2 | -7.9 | 18.6 | -2.26 |
| P19 | 15.19 | 19.1 | 25.4 | -6.3 | 19.7 | -0.62 |
